# Validation and standardization of DNA extraction and library construction methods for metagenomics-based human fecal microbiome measurements

**DOI:** 10.1186/s40168-021-01048-3

**Published:** 2021-04-29

**Authors:** Dieter M. Tourlousse, Koji Narita, Takamasa Miura, Mitsuo Sakamoto, Akiko Ohashi, Keita Shiina, Masami Matsuda, Daisuke Miura, Mamiko Shimamura, Yoshifumi Ohyama, Atsushi Yamazoe, Yoshihito Uchino, Keishi Kameyama, Shingo Arioka, Jiro Kataoka, Takayoshi Hisada, Kazuyuki Fujii, Shunsuke Takahashi, Miho Kuroiwa, Masatomo Rokushima, Mitsue Nishiyama, Yoshiki Tanaka, Takuya Fuchikami, Hitomi Aoki, Satoshi Kira, Ryo Koyanagi, Takeshi Naito, Morie Nishiwaki, Hirotaka Kumagai, Mikiko Konda, Ken Kasahara, Moriya Ohkuma, Hiroko Kawasaki, Yuji Sekiguchi, Jun Terauchi

**Affiliations:** 1 Biomedical Research Institute, National Institute of Advanced Industrial Science and Technology (AIST), Tsukuba, Ibaraki 305-8566 Japan; 2 Japan Microbiome Consortium (JMBC), Osaka, Osaka 530-0011 Japan; 3 Chitose Laboratory Corp., Kawasaki, Kanagawa 216-0041 Japan; 4 Biological Resource Center, National Institute of Technology and Evaluation (NITE), Kisarazu, Chiba, 292-0818 Japan; 5 Microbe Division/Japan Collection of Microorganisms, RIKEN BioResource Research Center, Tsukuba, Ibaraki 305-0074 Japan; 6 Institute of Food Sciences and Technologies, Ajinomoto Co., Inc., Kawasaki, Kanagawa 210-8681 Japan; 7 Laboratory for Innovative Therapy Research, Shionogi and Co., Ltd., Toyonaka, Osaka, 561-0825 Japan; 8 Japan Tobacco Inc., Minato, Tokyo, 105-6927 Japan; 9 TechnoSuruga Laboratory Co., Ltd., Shizuoka, Shizuoka 424-0065 Japan; 10 Infectious Diseases Unit, Department of Medical Innovations, New Drug Research Division, Otsuka Pharmaceutical Co., Ltd., Tokushima, Tokushima 771-0192 Japan; 11 Tsumura Kampo Research Laboratories, Tsumura & Co., Ami, Ibaraki 300-1192 Japan; 12 Biofermin Pharmaceutical Co., Ltd., Kobe, Hyogo 650-0021 Japan; 13 CDM Center Division 4, Takara Bio Inc., Kusatsu, Shiga 525-0058 Japan; 14 Molecular Genetic Research Department, Advanced Technology Center, LSI Medience Corporation, Chiyoda, Tokyo 101-8517 Japan; 15 H.U. Group Research Institute G.K., Hachioji, Tokyo 192-0031 Japan; 16 JSR-Keio University Medical and Chemical Innovation Center, Shinjuku, Tokyo 160-8582 Japan; 17 Ono Pharmaceutical Co., Ltd., Osaka, Osaka 541-8564 Japan

**Keywords:** Human microbiome, Metagenomics, Gut microbiota, Standardization, Accuracy, reproducibility, and comparability, Industrialization, DNA extraction, Library construction

## Abstract

**Background:**

Validation and standardization of methodologies for microbial community measurements by high-throughput sequencing are needed to support human microbiome research and its industrialization. This study set out to establish standards-based solutions to improve the accuracy and reproducibility of metagenomics-based microbiome profiling of human fecal samples.

**Results:**

In the first phase, we performed a head-to-head comparison of a wide range of protocols for DNA extraction and sequencing library construction using defined mock communities, to identify performant protocols and pinpoint sources of inaccuracy in quantification. In the second phase, we validated performant protocols with respect to their variability of measurement results within a single laboratory (that is, intermediate precision) as well as interlaboratory transferability and reproducibility through an industry-based collaborative study. We further ascertained the performance of our recommended protocols in the context of a community-wide interlaboratory study (that is, the MOSAIC Standards Challenge). Finally, we defined performance metrics to provide best practice guidance for improving measurement consistency across methods and laboratories.

**Conclusions:**

The validated protocols and methodological guidance for DNA extraction and library construction provided in this study expand current best practices for metagenomic analyses of human fecal microbiota. Uptake of our protocols and guidelines will improve the accuracy and comparability of metagenomics-based studies of the human microbiome, thereby facilitating development and commercialization of human microbiome-based products.

Video Abstract

**Supplementary Information:**

The online version contains supplementary material available at 10.1186/s40168-021-01048-3.

## Introduction

Our increased knowledge and mechanistic understanding of the role of human microbiota in health and disease [1–3] has created numerous opportunities for developing new strategies to improve human health through beneficial modulation of its microbiome [4, 5]. Correspondingly, global interest in the industrialization and commercialization of the therapeutic potential of the human microbiome has surged in sectors related to diagnostics, drug development, food, personnel care products, etc. In response, the Japan Microbiome Consortium (JMBC) was established by the industry in Japan to identify and address precompetitive needs to support and accelerate the development of commercially viable products for the microbiome market.

Analysis of microbiomes by metagenomics plays an indispensable role at many stages of pipelines for microbiome-based product development, from identification of microbial targets to clinical trials and product manufacturing. Workflows for metagenomics are however complex and prone to bias and errors at all steps, from sample collection and storage [6, 7] to DNA extraction [8, 9], sequencing and bioinformatics analysis [10, 11]. Methodological bias can lead to substantial differences in observed microbiota profiles, resulting in considerable variability in results across studies and laboratories using different protocols [12, 13]. To improve consistency and enhance confidence in the accuracy of measurement results, standardization of metagenomic analysis methods has thus been recognized as a pressing need by industrial and regulatory sectors [14].

Over the years, several efforts have been undertaken toward the standardization of metagenomics, including the development of microbial reference materials [15], consideration of best practices [16, 17], and proposal of “standard” methods for DNA extraction [9, 18]. To reach consensus and promote uptake of measurement standards by the industry, integrated studies that systematically compare the wide diversity of protocols available and validate their transferability and reproducibility across laboratories in the industry remain however necessary. Moreover, guidance for routine monitoring of analytical performance and testing of new methods need to be established, including target values for achievable performance, to ensure reproducibility and comparability of results across methods and laboratories/studies.

This study set out to establish standards-based solutions to support implementation of metagenomics by the microbiome industry. More specifically, in the first phase of this study (Fig. S1), we used newly developed mock communities for head-to-head comparison of a wide range of protocols for DNA extraction and sequencing library construction, to identify performant protocols and pinpoint important sources of measurement bias. In the second phase, we established standard operating procedures (SOPs) for selected protocols and evaluated their performance with respect to the variability of measurement results within a single laboratory (that is, intermediate precision) as well as interlaboratory transferability and reproducibility in a collaborative study involving nine industry-based laboratories. We further ascertained the performance of our recommended protocols in the context of an international collaborative study (that is, the MOSAIC Standards Challenge) [19]. Finally, we used defined performance metrics to set best practice target values for achievable analytical performance to guide validation of alternative protocols and routine quality management. Taken together, this study generated approximately 1 Tbp of sequencing data across more than 400 libraries, culminating in a set of validated protocols and methodological guidance for conducting metagenomic analysis of human fecal samples.

## Results

### Study considerations

To compare and validate protocols, the accuracy of measured taxonomic profiles was considered a key performance indicator. Following ISO Standard 5725, accuracy of analytical measurements and methods is defined as the closeness of agreement of results to the accepted reference value and consists of two components, namely trueness and precision [20]. Trueness reflects the closeness of agreement between the average of repeated measurements and the accepted reference value. Precision reflects the variability of repeated measurements and is typically evaluated at three levels, namely repeatability, intermediate precision, and interlaboratory reproducibility. As a measure of agreement, differences between measured and expected taxonomic compositions were quantified as the geometric mean of taxon-wise absolute fold-differences (denoted gmAFD), with trueness and accuracy being calculated based on the mean of replicated measurements and individual measurements, respectively. Precision of repeated measurements was expressed as the quadratic mean of taxon-wise coefficients of variation (denoted qmCV) of measured abundances, either calculated directly or based on the compositional metric variance (see Methods).

In this study, most experiments for benchmarking protocol performance employed mock communities with known compositions (“ground truth”) to allow evaluation of trueness/accuracy. Precision can be evaluated with complex biological samples without known composition. A strength of using actual biological samples, such as feces, is that they are fully commutable with samples of interest and could also reveal variability that may not be apparent with low-diversity mock communities [21]. As such, our study used DNA and cell mock communities for comparing methods, and mock communities plus human fecal samples for assessing intermediate precision and interlaboratory reproducibility (Fig. S1).

The mock communities developed in this study consisted of bacterial strains that are representative of human-associated microbiota of healthy Japanese individuals [22–25], mainly bacteria found in the gastrointestinal tract (Table S1). Strains covered a wide range of genomic guanine-cytosine (GC) contents (31.5 to 62.3%) and included multiple strains with reported Gram-positive-type cell walls (hereafter referred to as Gram-positives), to adequately challenge the library construction and DNA extraction protocols. The DNA mock community consisted of an equimolar amount of genomic DNA of 20 different strains, with values (“ground truth”) for their relative abundances assigned by fluorometric quantification of the concentrations of individual DNA stocks. The cell mock community contained the same bacteria, except for two strains (namely, *Megamonas funiformis* JCM 14723^T^ and *Megasphaera elsdenii* JCM 1772^T^) that proved difficult to enumerate by flow cytometry. Ground truth relative abundances for the cell mock community were assigned based on measurement of the total DNA content of individual cell stocks by quantification of adenine content directly from whole cells [26], as described in the Methods.

### Comparison of protocols for sequencing library construction

Using the DNA mock community, we compared the performance of eleven commercial kits for sequencing library construction (see Table S2 for a description of kits and their one-letter identifiers used below). Six of the kits employ physical DNA fragmentation by focused ultrasonication, four kits use specific nucleases for DNA digestion and one kit is based on a bead-based transposition reaction. Kits were evaluated in PCR-free format (denoted as X0, where X represents the one-letter kit identifier), if applicable, starting from 500 ng of input DNA and using PCR for library amplification, starting from 50 ng (low PCR cycles, XL) or 1 ng (high PCR cycles, XH) of input DNA. A total of 28 different conditions were evaluated and measurements performed in triplicate to assess technical repeatability. We note that for evaluation of protocols using ultrasonication, DNA fragmentation was performed at a high DNA concentration and varying amounts of fragmented and purified DNA then subjected to library construction. Following library preparation and sequencing on a NextSeq 500 instrument, relative abundances were estimated by pseudo-alignment of reads against the reference genome sequences using kallisto [27], based on its near-perfect accuracy using simulated sequencing reads (see Methods). For all protocols, repeatability was high, with a variability across technical replicates of 0.9 ± 0.5% (qmCV, mean and standard deviation across protocols). Protocols using PCR and starting from low DNA input amounts resulted in slightly poorer repeatability for some kits (Fig. S2).

**Fig. 1:**
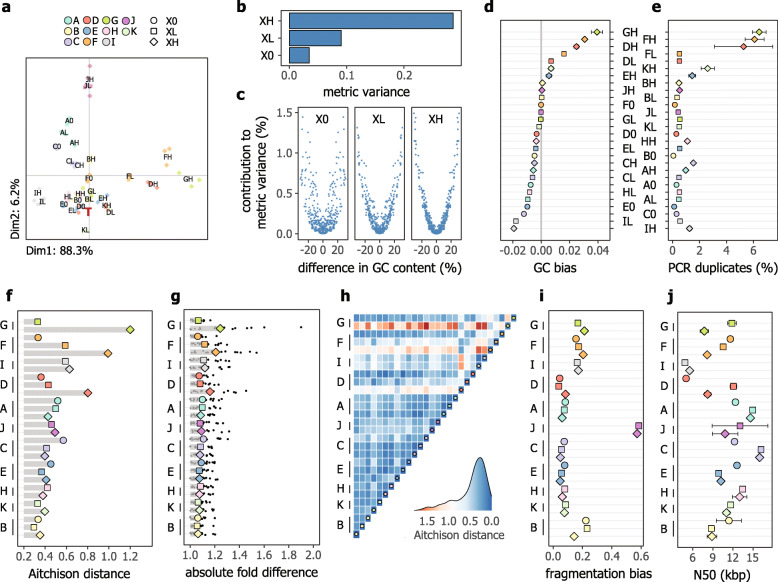
Comparison of protocols for sequencing library construction. **a** Compositional PCA ordination plot of measured DNA mock community compositions, based on clr (centered log ratio) transformed abundances. The red bold letter T depicts the expected composition (“ground truth”) projected onto the PCA ordination and symbols show individual replicates. Values in the axis labels represent the percentage of variance explained. Protocol identifiers were overlayed with jitter to prevent overlapping labels. **b** Dependence of the metric variance of measured compositions on DNA input amount and corresponding PCR conditions for library amplification (X0, XL, and XH). **c** Relationship between differences in genomic GC content of pairs of genomes/strains and their contribution to the metric variance shown in panel **b**. **d** Protocol-dependent variation in quantification bias due to genomic GC content. The GC bias metric represents the slope of the intercept-free linear regression line of log_2_-transformed abundance ratios for all possible pairs of strains to their differences in genomic GC content (see Fig. S3). **e** Variation in proportion of PCR duplicates. Protocols are ordered along the *y*-axis as in panel **d** and both panels share a common *y*-axis. **f**, **g** Closeness of agreement between the ground truth and measured compositions, expressed in terms of Aitchison distances (f) and absolute fold-differences (g). Kits are ranked along the *y*-axis based on Aitchison distances, averaged across DNA input amounts for each of the kits. For panel **g**, colored symbols show the geometric mean of strain-wise absolute fold-differences to the ground truth (that is, gmAFD) and black circles represent fold-differences for individual strains. **h** Heatmap of pairwise Aitchison distances showing quantitative consistency of measured compositions among protocols. **i** Variation in fragmentation bias, expressed as Aitchison distances between observed and expected base frequencies averaged across the first fifteen cycles of the forward read (see Fig. S4). **j** Variation in N50 values of the DNA mock community metagenome assemblies. For panels **g**–**j**, protocols are sorted as in panel **f**. For panels **b**, **c**, and **f**–**h**, values were computed based on the center (compositional mean) of three technical replicates. For panels **d**, **e**, **i**, and **j**, results are shown as the mean (symbols) and standard deviation (error bars), if visible, of three technical replicates. Across all panels, common symbol fill colors and shapes reflect kits and DNA input amounts, respectively, as shown in the legend of panel **a**

Consistent with their tight clustering along the first principal component in the PCA plot shown in Fig. 1a, variability of measured compositions, expressed as the metric variance (see Methods), was smallest for the subset of protocols evaluated in PCR-free format (Fig. 1b). Considering all protocols, library amplification by PCR led to higher variability, especially for low DNA inputs and associated higher number of PCR cycles for library amplification. Further, pairs of strains with larger differences in genomic GC content had higher logratio variances and thus contributed more to the metric variance (Fig. 1c).

To summarize quantification bias due to genomic GC content, log-transformed abundance ratios for all possible pairs of strains/genomes were regressed to the corresponding differences in genomic GC content (Fig. S3). The slope of the intercept-free linear regression model was then interpreted as an overall measure of GC bias, with negative slopes indicating bias against strains/genomes with higher GC contents for the even mock community. This analysis revealed that protocol I resulted in the largest overrepresentation of lower-GC genomes (Fig. 1d), with a 1.14-fold abundance ratio for genomes with a 10% difference in GC content, as estimated based on the slope of the linear fit. In comparison, higher GC genomes were overrepresented in libraries constructed with the related protocols D, F, and G when using low input DNA amounts, with an abundance ratio of 1.25-fold (averaged across protocols DH, FH, and GH) for strains with a 10% difference in GC content. Under the conditions tested, several of the latter protocols also resulted in libraries with a higher proportion of PCR duplicates (Fig. 1e).

Next, we ranked protocols according to the closeness of agreement between measured compositions, averaged across technical replicates, to the “ground truth.” Comparable and high agreement with the ground truth was observed for a range of protocols, including protocols using physical or enzymatic DNA fragmentation, and with or without library amplification by PCR (Fig. 1f). The geometric mean of strain-wise absolute fold-differences to the ground truth (that is, gmAFD) ranged from 1.06× for protocol BL to 1.24× for protocol GH (Fig. 1g). Further, protocols with high agreement to the ground truth generally showed excellent pairwise consistency, and several pairs of protocols showed high quantitative agreement but deviated more from the ground truth (Fig. 1h). Further, the low quantification bias of protocols using enzymatic DNA fragmentation also showed that increased non-random DNA fragmentation during library construction (Fig. 1h and Fig. S4) does not necessarily lead to poorer quantitative performance.

Based on the assembly of two million random read pairs, mimicking a moderately shallow sequencing depth, no major differences among library construction protocols were evident. Most assemblies had N50 values exceeding 8 kbp, except for protocols D0, GH, IH, and IL (Fig. 1j). Assemblies with lower N50 were typically derived from sequencing libraries with smaller fragment sizes (Fig. S5). Finally, base call error rates were largely comparable across protocols, although positional effects were observed in some cases (Fig. S6).

Based on their quantitative performance, cost, and hands-on time (see Table S2), we selected two kits as the basis for our SOPs for sequencing library construction, namely kits B and K. Unless stated otherwise, protocol BL (that is, kit B with 50 ng of input DNA) was used for library construction in all subsequent experiments.

By considering differences between the “ground truth” assigned by fluorometric DNA quantification and compositions measured with sequencing libraries constructed by PCR-free methods using physical DNA fragmentation, we finally determined an acceptable level of error associated with sequencing library construction (see Supplementary Methods). These values (namely 1.15 and 1.5 for the gmAFD and maximum AFD, respectively; see Table S3), are intended to be used for evaluating performance using our DNA mock community and could be considered best practice target values for achievable accuracy or trueness, with the recognition that the values may not directly be applicable to different mock communities.

### Comparison of protocols for DNA extraction

We used the cell mock community to evaluate nine different protocols for DNA extraction (Table S4), namely 7 commercial kits, an in-house phenol/chloroform-based protocol (protocol P), and the QIAamp DNA Stool Mini Kit based protocol recommended by the International Human Microbiome Standards (IHMS) consortium (protocol Q) [18]. All protocols involved cell lysis by bead-beating in individual tubes, except for protocol O, which used a deep-well plate format. By varying bead-beating regimes, a total of 21 conditions were evaluated. Following our SOP, libraries were prepared with protocol BL and, as for the DNA mock community, quantification of relative abundances was performed by kallisto. Technical replicates (*n* = 2 or 3) showed good to excellent repeatability, with a qmCV of 2.6 ± 1.8% across protocols (Fig. S7). For the cell mock community, all protocols produced comparable yields of high-molecular-weight DNA suitable for metagenome sequencing (Fig. S8).

**Fig. 2:**
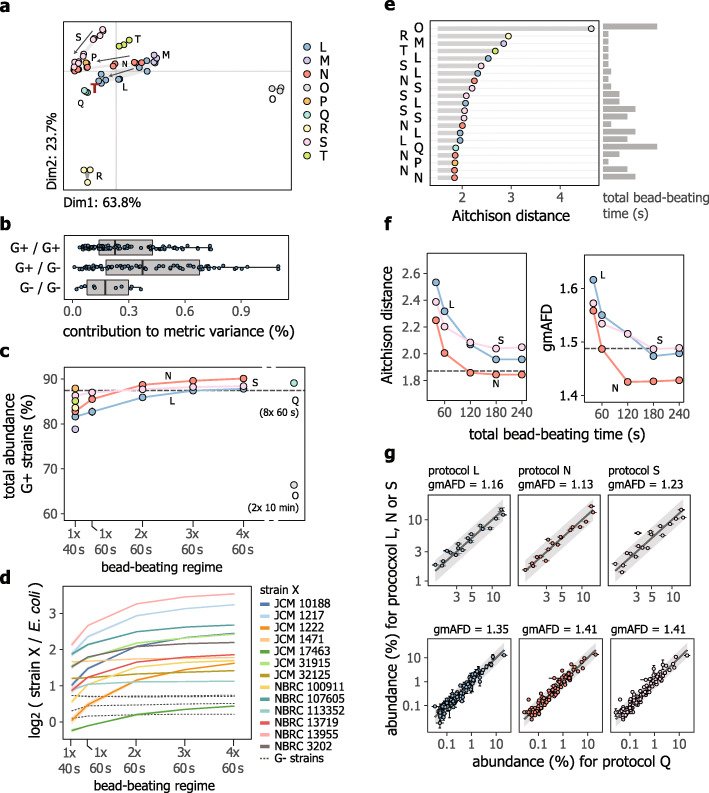
Comparison of protocols for DNA extraction. **a** Compositional PCA ordination plot of measured cell mock community compositions, based on clr (centered log ratio) transformed abundances. The red bold letter T shows the expected composition (“ground truth”) projected onto the PCA ordination and symbols represent individual replicates. Values in the axis labels represent the percentage of variance explained. For protocols N, L, and S, arrows show approximate trajectories of measurements for DNA extractions performed with increasing total bead-beating time. **b** Relationship between the Gram-type cell walls of pairs of strains and their contribution to the metric variance across protocols. **c** Cumulative relative abundance of Gram-positives (denoted as G+) as a function of bead-beating regime. The dashed horizontal line represents the expected proportion. **d** Measured abundances of different strains, relative to *E. coli* strain NBRC 3301, as a function of bead-beating regime for protocol N. Colors represent different Gram-positives and results for all Gram-negatives are shown as dotted gray lines. **e** Ranking of protocols based on the closeness of agreement between the ground truth and measured compositions, expressed in terms of Aitchison distances. **f** Effect of total bead-beating time on agreement between measured compositions and the ground truth, expressed in terms of Aitchison distances (left panel) and gmAFDs (right panel). Horizontal dashed lines represent corresponding values for protocol Q. **g** Scatter plots showing quantitative agreement between community profiles measured with protocol Q (*x*-axis) and protocols L, N, and S (*y*-axis) for the cell mock community (upper plots) and fecal sample S01 (lower plots). For the fecal sample, relative abundances were calculated as the percentage of reads assigned to a given species by kraken2. Gray areas represent up to 1.5- or 2-fold differences for the upper and lower plots, respectively. Data represent the mean and standard deviation of two or three technical replicates and corresponding gmAFDs calculated based on the means are indicated in the facet labels. For panels **c** and **d**, results are shown as the mean (symbols or lines) and the standard deviation (error bars or ribbons, if visible) of two or three technical replicates. For panels **e** and **f**, values were computed based on the center (compositional mean) of two or three technical replicates. Across all panels, common symbol and line colors reflect DNA extraction protocols/kits, as shown in the legend of panel **a**

Protocol O, which clearly separated along the first principal component in Fig. 2a, yielded a considerably lower proportion of total Gram-positives (Fig. S9), suggesting less efficient DNA release by bead-beating in multiwell plates as compared with in tubes. Further, considering all conditions tested, pairs of strains with different types of cell walls had, on average, a larger contribution to the metric variance whereas pairs of strains with Gram-negative-type cell walls (Gram-negatives) had the smallest contribution (Fig. 2b). Still, substantial variability was observed for some Gram-negatives. For example, protocol R, which separated along the second principal component Fig. 2a, led to a noticeably higher abundance of the high-GC bacterium *Pseudomonas putida* NBRC 14164^T^ (Fig. S9).

The total abundance of Gram-positives increased monotonically with total bead-beating time (Fig. 2c). Using at least two 1-min bead-beating cycles yielded total abundances of Gram-positives that were comparable with the expected amount and consistent with the results of protocol Q. Further, bead-beating time had no strong effect on library fragment size (Fig. S10), showing that the moderate increase in DNA shearing by extended beat-beating (Fig. S8) was largely inconsequential for short-read sequencing library construction and quantification of strain abundances. For protocol N, we further found increased abundance of most of the Gram-positives in the mock community whereas the abundance of Gram-negatives remained relatively constant (Fig. 2d), as compared with *Escherichia coli*, a Gram-negative bacterium that is typically considered easy-to-lyze and recover DNA from. A similar analysis for protocols L and S yielded more complex patterns, with variable responses among Gram-positives and Gram-negatives (Fig. S11).

As for the comparison of sequencing library construction protocols, we next evaluated protocol performance based on the closeness of measured compositions to the “ground truth,” as assigned based on total DNA content quantification. As shown in Fig. 2e, protocols that employed a single 40-s bead-beating cycle generally displayed the poorest agreement with the ground truth, with exception of our in-house protocol P. The latter achieved effective recovery of DNA from Gram-positives and high overall agreement to the ground truth with a bead-beating time of 40 s, presumably due to the presence of phenol/chloroform in the lysis buffer. Further, ranking of protocols based on their agreement with protocol Q was broadly consistent with ranking based on agreement to the ground truth (Spearman’s *r* of 0.93, Fig. S12). For protocols L, N, and S, longer total bead-beating times consistently improved agreement with the ground truth, reaching levels comparable with or slightly superior to that of protocol Q (Fig. 2f). For these protocols, measured abundances in the cell mock community differed approximately 1.2-fold (gmAFD) from abundances measured with protocol Q (Fig. 2g).

Based on its DNA yield, efficient recovery of DNA from Gram-positives and overall small bias, as well as its rapid turnaround time and low cost (see Table S4), we selected protocol N as the basis for our SOP for DNA extraction. Cross-validation of protocol N, with a bead-beating regime of 3 × 60 s, against protocol Q further showed good agreement of observed microbiota profiles for fecal sample S01 (Fig. 2g), with a gmAFD of species-wise abundances of approximately 1.4-fold when considering species with a mean abundance of at least 0.05% for either protocol Q or N.

Finally, as for the DNA mock community, we set an acceptable level of errors that may be considered best practice target values for performance as assessed using our cell mock community. By considering differences between the “ground truth” and values measured for DNA extracted with protocols P and Q, the gmAFD was set to 1.55 and the maximum AFD to 3.1 (Table S3). We recognize that these thresholds are dominated by methodological bias of HPLC- and metagenomics-based quantification and are thus expected to become narrower as techniques for preparing microbial cell-based reference materials improve.

### Intermediate precision and interlaboratory reproducibility

We next validated performant protocols identified in the first phase of this study by evaluating their intermediate precision (that is, within-laboratory variability of measurement results) and interlaboratory transferability and reproducibility. For sequencing library construction, kits B and K, both with varying DNA input amounts, and protocol BL (that is, kit B with 50 ng of input DNA) were evaluated with respect to intermediate precision and interlaboratory reproducibility, respectively. For DNA extraction, protocol N with a bead-beating regime of 3 × 60 s was evaluated for both intermediate precision and interlaboratory reproducibility.

For intermediate precision, we considered two variables that are commonly changed within a single laboratory, namely operator and reagent lot. Based on the relatively small number of operators and reagent lots evaluated (see Fig. S13), we combined both variables into a single factor and thus assessed the combined (operator+lot)-different intermediate precision.

For the interlaboratory study, frozen samples (that is, the two mock communities and five fecal samples) were shipped to external laboratories from a range of industries in Japan (Fig. S13). The participants received no dedicated training with the specific protocols and processed each of the samples in duplicate following the SOPs provided by the central laboratory that established the SOP and coordinated the interlaboratory study.

Assessment of the reproducibility of the SOP for sequencing library construction using the DNA mock community involved four laboratories (that is, three external industry-based laboratories and the central laboratory). Each participant constructed sequencing libraries and shipped frozen aliquots back to the central laboratory for sequencing (Fig. S13 and Table S5). In addition, each laboratory performed sequencing locally and shared raw sequencing reads with the central laboratory for processing and analysis. Data generated by central sequencing allowed us to estimate variation in measurement results due to library construction, without confounding variability that may be introduced by sequencing. Further, centrally generated sequencing data were compared with data produced locally by the participating laboratories to assess differences in strain abundances due to varying sequencing runs/instruments.

Assessment of the reproducibility of the SOP for DNA extraction involved ten laboratories (that is, the nine external industry-based laboratories and the central laboratory). Each laboratory extracted DNA from the cell mock community and a single fecal sample (that is, feces S01) and shipped frozen aliquots of extracted DNA back to the central laboratory for library construction, using protocol BL, and sequencing. This experiment allowed us to estimate variability associated with DNA extraction, without confounding interlaboratory variation due to library construction and sequencing. Further, three of the participating laboratories also provided DNA extracted using custom methods, which allowed us to compare realistic differences due to varying methods across laboratories to the interlaboratory variability observed for the SOP. All custom protocols involved cell lysis by bead-beating but employed varying lysis buffers/equipment and procedures for DNA purification. Finally, each of the four laboratories involved in the assessment of the reproducibility of library construction (see above) also processed and sequenced five fecal samples (that is, sample S01 and four additional samples, denoted S02, S03, S06, and S13) and provided raw sequencing reads to the central laboratory for processing and analysis. All sequencing at the central laboratory and participants’ facilities was performed with a NextSeq 500/550 instrument, except for a single industry-based laboratory that used the NovaSeq platform.

**Fig. 3:**
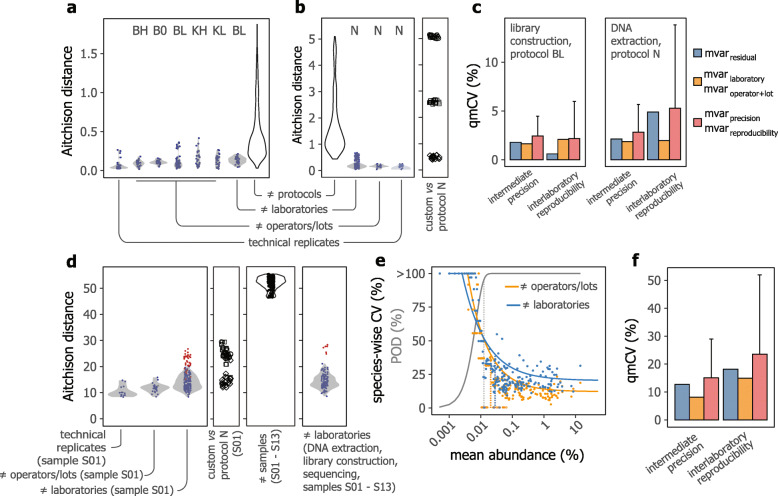
Evaluation of intermediate precision and interlaboratory reproducibility of SOPs for sequencing library construction and DNA extraction. **a**, **b** Distribution of pairwise Aitchison distances of replicated measurements associated with different operator+lot combinations and laboratories, for library construction (**a**) and DNA extraction (**b**), as evaluated using the DNA and cell mock community, respectively. Violin plots depict the distribution of all pairwise distances and symbols shown individual datapoints. If applicable, protocol identifiers are indicated. The subpanel in **b** shows distances between three custom DNA extraction protocols (shown as different shapes) and protocol N. **c** Bar charts of estimated qmCVs attributed to different components of variance. Error bars for the intermediate precision and interlaboratory reproducibility estimates represent one-sided 95% confidence intervals. **d** Similar to panel **b**, for fecal samples. The first two subpanels show results for fecal sample S01. The third subpanel shows distances between different samples (that is, feces from different donors, denoted as S01 to S13) based on measurements performed by the central laboratory, with DNA extraction and library construction performed using protocols N and BL, respectively. The fourth subpanel shows distances between replicated measurements performed by four laboratories for samples S01 to S13, with all steps (that is, DNA extraction, library construction and sequencing) performed by the participating laboratories. Data from laboratories for which at least one of the duplicate measurements was considered an outlier are shown as red symbols in the first and fourth subpanels (see Fig. S17). **e** Relationship between species-wise coefficients of variation (CVs) of measured relative abundances and mean relative abundances across replicated measurements to calculate the LOQ (fitted CV of 40%) under different levels of replication as indicated by the colors. Note that CV values exceeding 100% were set to 100% for visualization purposes only. The gray line represents the fit of a species’ probability of detection (POD) to its mean abundance to estimate the LOD (see Fig. S18). **f** Bar charts of estimated qmCVs attributed to different components of variance for fecal sample S01. Error bars for the precision and reproducibility estimates are one-sided 95% confidence intervals. For panels **c** and **f**, fill colors show the metric variance component based on which the corresponding qmCV values were calculated (see Supplementary Methods)

For the DNA and cell mock communities, all measurements performed following our SOPs had an agreement to the “ground truth” that was better than the best practice target values set in the first phase of this study (Fig. S14). Next, we focused on data generated by central library construction, if applicable, and sequencing, and summarized variability of measurement results as distributions of pairwise Aitchison distances. As shown in Fig. 3a for library construction and Fig. 3b for DNA extraction, distances between measurements performed by changed operators and/or using different reagents lots and by different laboratories were only slightly higher than distances between technical replicates, and well below overall differences observed for the range of protocols evaluated in the first phase of this study. For DNA extraction, two out of three custom protocols evaluated by participating laboratories resulted in more dissimilar compositions, as can be seen based on their higher distances to results generated with protocol N (Fig. 3b). As such, these two measurement results also failed to meet our target values for achievable accuracy for the cell mock community (Fig. S14).

We next performed decomposition of variance components by analysis of variance (ANOVA) based on Aitchison distances and summarized variability in terms of qmCV values estimated from resulting metric variance components (see Supplementary Methods). With respect to sequencing library construction, both intermediate precision and interlaboratory reproducibility were high, with a qmCV of approximately 2% for protocol BL when sequencing was performed at the central laboratory in a single sequencing run (Fig. 3c). All other evaluated protocols (BH, B0, KH, and KL) similarly had an intermediate precision of 3% or better, consistent with their comparable distribution of pairwise Aitchison distances shown in Fig. 3a. In comparison, interlaboratory reproducibility of DNA extraction was roughly twofold lower, with a qmCV of approximately 5% (Fig. 3c).

Comparison of centrally and locally (that is, by the external laboratories) generated sequencing data further revealed run and/or instrument-specific bias in strain-wise abundances, strongly associated with GC content (Fig. S15). Similarly, repeated sequencing of a single DNA mock community library, prepared using protocol C0 at the beginning of this study (that is, prior to the establishment of our SOP based on kit B), across four NextSeq 500 sequencing runs at the central laboratory showed a variability of 2.6% (qmCV), with the highest strain-wise variation attributed to strains/genomes with high and low GC contents (Fig. S15).

Intermediate precision and interlaboratory reproducibility of DNA extraction were further evaluated using fecal sample S01, with measured microbiota profiles expressed as the proportion of reads assigned to different species by kraken2. As shown in Fig. 3d, pairwise Aitchison distances between measurements performed by a different operator and/or reagent lots were largely comparable with distances between technical replicates (see Fig. S16 for Bray-Curtis dissimilarities). Variability due to changed laboratories was slightly higher but remained well below the biological variation between fecal samples from different individuals (Fig. 3d). As for the cell mock community, extraction of DNA using custom methods by the external laboratories led to larger differences for two of the protocols, as can be seen by their increased distances to compositions generated with protocol N (Fig. 3d). Of note here is that the custom DNA extraction protocol that was most comparable with protocol N for the cell mock community also provided the most consistent results for the fecal sample.

As shown in Fig. S17, data from one laboratory was considered an outlier in the above dataset (excluding custom DNA extraction protocols) to evaluate interlaboratory reproducibility of DNA extraction, using sample S01 and with central sequencing. In addition, data from another laboratory was scored as an outlier for fecal sample S03, for which all processing steps and sequencing were performed by the participating laboratory.

After exclusion of outliers, we next determined the limit-of-quantification (LOQ) by evaluating species-wise coefficients of variation of measured abundances as a function of their mean abundance, averaged across repeated measurements performed under different levels of replication (Fig. 3e; see Methods). The LOQ provides a valuable metric for evaluating protocol performance, with the recognition that LOQs are dependent on sequencing depth and bioinformatics tool used for taxonomic profiling. In addition, for species with an abundance below the LOQ, inferences about their (differential) abundance will not be reliable [28]. In a similar fashion, we defined the limit-of-detection (LOD) based on the probability of detection of a species in relation to its mean abundance (Fig. 3f and Fig. S18; see Methods). As for the LOQ, the LOD provides an informative metric for evaluating measurement and method performance and will also ensure meaningful interpretation of the presence or absence of species in fecal samples analyzed by different laboratories and/or operators.

Next, we considered species with a mean abundance exceeding the above-defined LOQs to quantify intermediate precision and interlaboratory reproducibility by distance-based ANOVA, as for the mock communities. As shown in Fig. 3f, interlaboratory reproducibility of DNA extraction was estimated to be 23% (qmCV as estimated based on the metric variance), compared with an intermediate precision of approximately 15%. We note however that these values may be moderately inflated due to within-sample heterogeneity that is challenging to fully eliminate for fecal samples.

Finally, similar reproducibility was found for five fecal samples, including sample S01, processed and sequenced across four different laboratories (Fig. S19), consistent with their comparable pairwise Aitchison distances shown in Fig. 3d. Comparison of reproducibility estimated from taxonomic profiles generated with kraken2 to that estimated based on taxonomic profiles produced by mOTUs2 further highlighted the impact of bioinformatics tools on perceived reproducibility (Fig. S19). While in-depth investigation of this observation was outside the scope of this study, this finding points to the need to benchmark the wide range of taxonomic profiling tools available with respect to their consistency with data generated by different laboratories.

Taken together, these data showed our SOPs for DNA extraction and sequencing library construction achieved high reproducibility both within a single laboratory and across laboratories. Based on our validation results, we further set a series of target values for repeatability, intermediate precision, and interlaboratory reproducibility (Table S6).

Differences in perceived reproducibility as assessed using the cell mock community and fecal sample(s) also underscored the importance of evaluating protocols with different types of samples to ensure generalizability of the results, as has been pointed out previously [12]. Here, we also recognize that our estimated reproducibility of DNA extraction across ten laboratories was based on a single fecal sample, in addition to the cell mock community. Although we found broadly consistent reproducibility of the complete workflow across five fecal samples (Fig. S19), it will thus be beneficial to evaluate additional samples from different donors to prevent potential bias due to sample-specific effects.

### Assessment of protocols using the MOSAIC Standards Challenge samples

To further evaluate our recommended protocols, we analyzed samples from the MOSAIC Standards Challenge and compared our results with publicly available data (*n* = 14 datasets; see Table S7). For DNA extraction, we used protocol N (bead-beating regime of 3 × 60 s) as well as protocols P and Q for cross-validation; sequencing libraries were generated with protocol BL. For fair comparison, public sequencing data were downloaded and processed using our default bioinformatics pipeline, including quality control, removal of human genomic reads, and taxonomic profiling using kraken2 or mOTUs2. If applicable, data were randomly downsampled to 5 million reads pairs prior to taxonomic profiling.

**Fig. 4:**
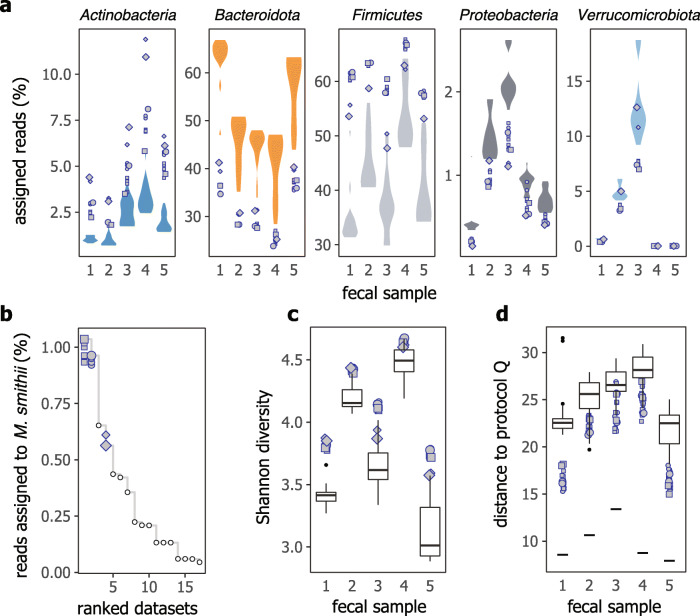
Assessment of the SOPs with the MOSAIC Standards Challenge samples. **a** Abundance of five major phyla across the five fecal samples (designated as 1 to 5 following the MOSAIC Standards Challenge naming), expressed as the proportion of reads assigned to each phylum. Violin plots show the distribution of publicly available data in the MOSAIC Standards Challenge database, and gray symbols show data for protocols N (squares), P (circles) and Q (diamonds), with individual measurements results shown for each protocol. **b** Proportion of reads assigned to the species *M. smithii* for fecal sample 2. Datasets are ranked by decreasing abundance, white circles are for public data, and gray symbols show individual results for protocols N, P and Q as in panel **a**. **c** Shannon diversity across fecal samples. **d** Distribution of Aitchison distances to protocol Q. The short black horizontal lines show the distance between two technical replicates for protocol Q. For panels **c** and **d**, gray symbols show individual results for protocols N, P and Q as in panel **a**, and boxplots show the distribution of public data in the MOSAIC Standards Challenge database. For the boxplots, the tick horizontal line represents the median, hinges show the 25th and 75th percentiles, whiskers extend to the largest and smallest value at most 1.5× the IQR (interquartile range) from the upper and lower hinges, respectively, and outlying datapoints beyond the end of the whiskers are shown as black circles. For all panels, larger symbols represent data deposited to the MOSAIC Standards Challenge website and smaller symbols are for additional replicates available in the SRA (see Table S8). Symbol shapes are common for all panels

In contrast to the observation that variability due to library construction protocols was generally relatively small, although several outliers were apparent (Fig. S20), taxonomic profiles for the fecal samples varied more substantially, even at the phylum level. For example, the abundance of the phylum *Firmicutes*, which generally consists of Gram-positives, was comparatively high for all protocols evaluated in this study (namely protocols N, P, and Q) whereas the abundance of the phylum *Bacteroidota* was relatively low, as determined by taxonomic profiling using kraken2 (Fig. 4a) and mOTUs2 (Fig. S21). These data showed that protocols N, P, and Q were effective in recovering DNA from difficult-to-lyze bacteria in fecal samples, in line with their excellent performance for the cell mock community. We also found that protocols P, N, and Q also yielded a high proportion of reads assigned to the species *Methanobrevibacter smithii* (Fig. 4b). This methanogenic archaeon is typically considered hard-to-lyze and differential abundance of this species has previously been reported in the gut microbiota of healthy Japanese individuals [22]. Consistent with the excellent recovery of DNA from Gram-positives, protocols P, N, and Q also resulted in communities with higher alpha diversity, in terms of the number of species (Fig. S22) and Shannon diversity (Fig. 4c). Further, alpha diversity was positively correlated with *Firmicutes*-to-*Bacteroidota* and *Actinobacteriota*-to-*Bacteroidota* abundance ratios (Fig. S23). It is here of note that all publicly available data were generated with DNA extraction protocols that employed mechanical disruption for cell lysis. Although the small number of available datasets precluded in-depth investigation of possible reasons for the observed differences between our protocols and public datasets, these findings underscore the importance of detailed SOPs, in addition to best practice guidelines, to achieve comparability across studies.

We further compared protocol N with protocol Q to cross-validate our recommended protocol to an alternative “standard” method. Protocol N generally showed the smallest differences in species-level taxonomic profiles to protocol Q as compared with other protocols (Fig. 4d), with the recognition that both datasets were generated in-house using a single protocol for library construction. In terms of the qmAFD metric, differences between protocols N and Q, calculated as the mean of all possible pairwise comparisons, ranged from 1.3-fold (for feces 5) to 1.7-fold (for feces 3), considering species with an abundance of at least 0.05% for either protocol N or Q in each pairwise comparison. Finally, inspection of species-wise variability in relative abundances showed that several Gram-positive species/genera tended to display the highest variation across measurements performed using varying protocols (Fig. S24).

## Discussion

Through evaluation of a wide range of protocols and assessment of intra- and interlaboratory reproducibility, we established validated protocols for DNA extraction from human fecal samples and sequencing library construction, identified important sources of bias, and provide guidance for achievable performance and routine quality management (Fig. S25).

By considering analytical performance as well as cost and hands-on time, we retained two recommended protocols as the basis for our SOPs for sequencing library construction. More specifically, we recommend protocol B (QIAseq FX DNA Library Kit) for taxonomic profiling based on its high accuracy of quantification of the DNA mock community, low GC bias, and excellent transferability across laboratories owing to the use of enzymatic DNA fragmentation. In addition, protocol B can be run with and without PCR across a wide range of DNA input amounts and, while not evaluated here, is also amenable to automation using robotic liquid handlers. Protocol K (SMARTer ThruPLEX DNA-seq Kit), which uses focused ultrasonication for DNA fragmentation, is our recommended protocol for applications where more precise control over library fragment size is beneficial, as is the case for metagenome assembly. Although bias in (meta)genomics analyses due to library preparation methods has been relatively well documented (e.g., [29, 30] and references below), recommendations of specific protocols and standardization of the library construction step in the (human) microbiome field has generally been limited. As such, our study and suggested protocols are expected to improve the reproducibility of microbiome measurements by expanding current standards and best practices that mainly focused on DNA extraction.

Based on its DNA yield, overall accuracy as evaluated using the cell mock community, and efficient recovery of DNA from Gram-positives, protocol N (ISOSPIN Fecal DNA kit, with a bead-beating time of 3 × 60 s) is recommended for DNA extraction from human fecal samples. Protocol N achieves an accuracy comparable with that of IMHS’ protocol Q for the cell mock community and comparable taxonomic profiles for fecal samples, with differences well below biological variation between individuals. Further, protocol N proved highly transferable across laboratories and required, in our hands, less time than protocol Q. In addition, manufacturing of the QIAamp DNA Stool Mini Kit, which is part of protocol Q, has been discontinued. We thus suggest that protocol N may serve as an alternative standard method for DNA extraction of human fecal samples.

Variations across protocols for library construction were relatively small, and a considerable range of protocols yielded an acceptable level of error (that is, differences to the “ground truth” for the DNA mock community). Poorer accuracy, as assessed using the DNA mock community, was generally associated with library amplification by PCR and strongly correlated with the genomic GC content of the strains. This is consistent with previous studies showing that PCR and GC content represent important causes of bias in (meta)genome sequencing workflows [31–33]. Use of PCR-free protocols or minimizing the number of PCR cycles should thus be considered best practices for library construction. The strong relationship between genomic GC content and bias also reinforced the importance of using microbial mock communities with a wide range of genomic GC contents, in addition to varying taxonomic affiliation, to sufficiently challenge protocol performance.

In comparison, protocols for DNA extraction displayed larger variability, even considering only methods using bead-beating as the main mechanism for cell lysis. Extended bead-beating was necessary for efficient recovery of DNA from Gram-positives and protocols that employed less vigorous bead-beating regimes failed to meet our recommended target values for achievable accuracy. This emphasizes previous recommendations to use mechanical lysis by bead-beating for effective recovery of DNA from Gram-positive microbes [8, 18, 34]. It also underscores the importance of evaluating DNA extraction protocols using mock communities that contain a wide range of microbes identified as sensitive to varying protocols (Fig. S24), such as for example the methanogen archaeon *M. smithii*.

In addition to pre-analytic procedures, we found that the sequencing step itself can lead to considerable variation in taxon abundances across laboratories and/or instrument types and that this bias was associated with genomic GC content. Current tools for metagenomic profiling typically do not account for GC content associated bias, thus warranting caution during data analysis and interpretation of estimated abundances [33]. Importantly, these findings also underscore the need for suitable controls at all steps of metagenomics workflows, including the sequencing step, to ensure that potential run- or instrument-dependent bias can be diagnosed and potentially corrected for.

We assigned values for relative abundances (“ground truth”) of the cell mock community by measuring the total DNA content of individual strains using non-enzymatic acid-catalyzed release of adenine directly from whole cells, a method that was previously developed for quantification of the efficiency of cell lysis and DNA release from bacterial cells [26]. Compared with cell abundances estimated by flow cytometry, abundances estimated based on total DNA content showed better concordance with metagenomics measurement results. This suggests that this technique may provide a valuable route for future development of more reliable cell-based microbiome reference materials, although more in-depth validation studies are warranted.

Finally, this study also proposes several metrics and recommended target values for evaluating the performance of methods and measurement results (Fig. S25). More specifically, we suggest thresholds for accuracy/trueness and precision that can be evaluated when developing and implementing new SOPs and routine quality management. Although more data and interlaboratory studies are needed, our study may thus serve as a starting point for future establishment of performance metrics and thresholds or acceptance criteria for microbiome community measurements. Widely accepted use of well-defined metrics will allow systematic comparison of method performance across studies, which is currently not feasible due to the widely varying metrics used to quantify performance [15].

## Conclusions

To conclude, we anticipate that the here recommended and validated protocols, as well as the proposed performance metrics, will contribute to and stimulate ongoing efforts to standardize and harmonize metagenomic analysis methods for the human microbiome. Looking ahead, this study can also promote more concerted efforts on a global scale that will be needed to reach consensus and establish widely used and accepted standards [15]. Adoption of such standards will advance microbiome research by ensuring reliability of the measurement results and thus facilitate commercialization of the therapeutic potential of the human microbiome in a range of industrial sectors.

## Methods

### Human stool samples

Human stool samples were collected from five healthy Japanese individuals; informed consent was obtained from all donors. Handling and processing of human-derived fecal samples was approved by the National Institute of Advanced Industrial Science and Technology (AIST, Japan), under number 71120030-A-20190201-001. Collected stool samples (denoted as S01, S02, S03, S06, and S13) were homogenized, distributed in single-use aliquots, and immediately stored at − 80 °C until use.

### MOSAIC Standards Challenge samples and public sequencing data

Samples from the MOSAIC Standards Challenge were obtained from The BioCollective (Denver, CO) and immediately stored at −80 °C until use. All samples were used as provided for DNA extraction, without any pretreatment, except for thawing of the sample, starting from approximately 20 mg (200 μl of sample) of biomass.

Publicly available sequencing data (January 2020 release) were downloaded from the MOSAIC Standards Challenge data repository (https://platform.mosaicbiome.com/workspaces/695/files, accessed on July 16, 2020; Table S7). Description of the protocols was extracted from the provided metadata (file “Standards_metadata_Jan_2020.tsv”).

### Preparation of the cell mock community

Bacterial cultures were obtained from the National Institute of Technology and Evaluation Biological Resource Center (NBRC) or Japan Collection of Microorganisms (JCM). Liquid cultures were prepared using the media and cultivation conditions shown in Table S1. Cells were collected during the late-log to stationary growth phase by centrifugation (15 min at 4,000×*g*), washed with phosphate-buffered saline (PBS, pH 7.4) and stored at −80 °C in PBS containing 15% glycerol as cryoprotectant.

Cell counts were determined by flow cytometry following staining of cells with SYTO 9 green fluorescent nucleic acid stain, using a CytoFLEX (Beckman Coulter) flow cytometer equipped with a 488-nm laser. Absolute cell concentrations were determined by adding 6-μm polystyrene microsphere counting beads (component of ThermoFisher Scientific’s Bacteria Counting Kit), using the CytExpert software (Beckman Coulter). All analyses were performed in triplicate and a minimum of 6000 bead signals were acquired for each measurement. A near-even cell mock community was then formulated by combining equal cell numbers of each of the strains. Single-use aliquots at a concentration of approximately 4 × 10^10^ cells/ml were prepared and stored at −80 °C until use.

Homogeneity of the material was evaluated by sequencing of DNA extracted from three aliquots, each in duplicate, using protocols N (bead-beating time of 3 × 60 s) and BL for DNA extraction and library construction, respectively. Distance-based analysis of variance (see details below) showed only minor variability among aliquots, with a qmCV of 1.1%, comparable with the technical variability of 1.2% (qmCV as estimated based on the residual variance).

### Determination of total DNA content for strains in the cell mock community

To assign reference values (“ground truth”) to the relative abundances of each strain in the cell mock community, we determined the DNA content, as mass per unit volume, of cell stocks of individual strains. Specifically, the adenine content of individual stocks was measured following the method of de Bruin and Birnboim [26], with minor modifications. Based on the known base composition and size of the genomes, this allowed us to calculate the total DNA content for each strain and subsequently their relative abundances in the cell mock community based on the mixing ratios calculated from the flow cytometric quantification of cell counts (see above).

Cell pellets were resuspended in distilled water and treated with HCl (200 mM final concentration in a total volume of 400 μl) for 60 min at 60 °C, with vigorous agitation in a temperature-controlled mixer (Eppendorf Thermo Mixer; 1,400 rpm). Subsequently, 133 μl of 1 N NaOH was added, and the solution incubated at 100 °C for 10 min, followed by centrifugation at 20,000×*g* for 5 min to remove cell debris. Recovered supernatants (400 μl) were then neutralized by addition of 40 μl of 1 N HCl and 160 μl of 400 mM ADA buffer (pH 6.6). After additional centrifugation at 20,000×*g* for 1 min, 40 μl of supernatant was subjected to high-performance liquid chromatography on an Alliance 2695 Separations Module with Photodiode Array Detector (Waters). Separation was performed using an Inertsil ODS-3 column (5 μm, 4.6 × 250 mm, GL Sciences) by isocratic elution at 25 °C with a mobile phase containing 2% of methanol, 30 mM of ammonium acetate, 1 mM of CDTA, and 10 mM of NaH_2_PO_4_ (pH 6.3). Using a flow rate of 0.5 ml/min, the retention time of adenine, measured at a wavelength of 260 nm, was around 50 min. Preparation of adenine standards for calibration and calculation of total DNA concentrations based on the known base composition of the genomic DNA of the strains were performed following de Bruin and Birnboim [26]. Finally, DNA concentrations were converted to genome copy numbers based on the known molecular weight of the genomes, calculated using the genome size and a molecular weight of 660 g per mole per base pair.

### Preparation of the DNA mock community

Extraction and quantification of genomic DNA was performed for each strain as follows. Cell pellets were suspended in 500 μl of buffer B1 (Qiagen) containing 10 mg/ml of RNase A (ThermoFisher Scientific), followed by lysis of the cells by bead-beating with a mixture of 5 mm, 0.2 mm and 0.1 mm Zirconia beads (Nikkato) for 2 × 10 s (speed of 4 m/s) using the FastPrep-24 instrument (MP Biomedicals). Additional enzymatic lysis was performed by addition of 50 μl of 100 mg/ml lysozyme (Sigma) and incubation at 37 °C for 1 h. Subsequently, 21 μl of proteinase K (Takara Bio) and 175 μl of buffer B2 (Qiagen) were added and the solution incubated at 55 °C for 1 h. The EZ1 DNA tissue kit (Qiagen) was then used for DNA purification. Total DNA concentrations were measured with the Quant-iT PicoGreen dsDNA Assay Kit (Invitrogen) and converted to genome copy numbers based on the known molecular weight of the genomes. Equimolar amounts of genomic DNA were then combined to obtain an even DNA mock community, containing a near-equal genome copy number of each strain. Aliquots at a concentration of 50 ng/μl were prepared and stored at −20 °C until use.

Homogeneity of the aliquots was evaluated by metagenome sequencing using protocol C0 for library construction, based on duplicate measurements of three aliquots. Analysis of variance showed that variability due to aliquots was smaller than technical variability, which had a coefficient of variation of 1% (qmCV as estimated based on the residual variance).

### Genome sequencing and assembly

Reference genome sequences for all strains in the mock communities were obtained from NCBI’s Genbank database or generated as part of this study for ten strains (see Table S1), as detailed in the Supplementary Methods.

### Protocols for sequencing library construction

To prepare DNA for evaluation of kits/protocols using physical fragmentation, 1 μg (per reaction) of DNA was fragmented by focused ultrasonication using the Covaris M220 instrument, with a target fragment size of 350 bp. Fragmented DNA was purified using the Agencourt AMPure XP PCR Purification system with a bead-to-sample ratio of 1.8:1 and eluted in low-EDTA Tris-HCl buffer (10 mM Tris-HCl, 0.1 mM EDTA, pH 8.0).

Purified DNA was subjected to PCR-free library construction starting from 500 ng of DNA for the following kits: Accel NGS 2S Plus DNA Library Kit (Swift Biosciences), TruSeq DNA PCR-Free Library Prep Kit (Illumina), KAPA HTP Library Preparation Kit (Roche), and KAPA HyperPrep Kit PCR-free (Roche). For lower DNA input amounts (50 ng and 1 ng of fragmented DNA), the following kits were evaluated using PCR for library amplification: Accel NGS 2S Plus DNA Library Kit, TruSeq Nano DNA Library Prep Kit, KAPA HTP Library Preparation Kit, KAPA HyperPrep Kit, NEBNext Ultra II DNA Library Prep Kit (New England Biolabs) and SMARTer ThruPLEX DNA-Seq Kit (Takara Bio).

Library construction with protocols using enzymatic DNA fragmentation was evaluated using the following all-in-one kits: QIAseq FX DNA Library Kit, NEBNext Ultra II FS DNA Library Prep Kit, and Nextera DNA Flex Library Prep Kit (Illumina). In addition, enzymatically fragmented DNA was generated using the KAPA Frag Kit for Enzymatic Fragmentation (Roche), followed by sequencing libraries constructions using the KAPA HyperPrep Kit and KAPA HTP Library Preparation Kit. Manufacturer recommended procedures were followed for all kits; the number of PCR cycles for each kit and DNA input amounts are provided in Table S2. If optional, size selection was performed using two sequential selections, with 0.6× and 0.8× bead volumes, using beads included in the kits or the AMPure XP PCR Purification system, as recommended in the manufacturer’s protocols.

### Protocols for DNA extraction

Extraction of genomic DNA was performed from 150 μl of cell mock community, approximately 200 mg (wet weight) of fecal material from donors in this study, or approximately 20 mg (200 μl) for samples from the MOSAIC Standards Challenge. Manufacturer recommended procedures were followed for each of the commercial kits evaluated, namely Extrap Soil DNA Kit Plus ver.2 (NIPPON STEEL Eco-Tech Corporation), FastDNA SPIN Kit for Feces (MP Biomedicals), ISOSPIN Fecal DNA Kit (Nippon Gene), MagAttract PowerMicrobiome RNA/DNA EP Kit (Qiagen), MORA-EXTRACT kit (Kyokuto Pharmaceutical), QIAmp PowerFecal Pro DNA kit (Qiagen), and Quick DNA Fecal/Soil microbe Miniprep Kit (Zymo Research). For protocol Q, IHMS’ SOP [18] was followed, using 300 mg of autoclaved 0.1-mm Zirconia beads and the QIAamp DNA Stool Mini Kit (Qiagen). For all protocols, except protocol O (see below), bead-beating was performed using the FastPrep-24 instrument (MP Biomedicals) at a speed of 6 m/s; bead-beating regimes evaluated for the different kits are provided in Table S4. If applicable, samples were kept for 5 min at room temperature between bead-beating cycles to prevent excessive heating of the sample. For protocol O, bead-beating was performed in 2-ml deep well plates using the TissueLyser II (Qiagen), for 2×10 min at a frequency of 20 Hz, followed by automated DNA purification using the EpMotion M5073 liquid handling system (Eppendorf). A detailed description of our in-house protocol P is provided in the Supplementary Methods.

### Sequencing, read processing and analysis

Unless stated otherwise, high-throughput sequencing was performed with a NextSeq 500 instrument using NextSeq 500/550 Mid Output Kit v2.5 (300 cycles, 2×151 bp reads). Base calling and demultiplexing were performed off-board using Ilumina’s bcl2fastq software v2.16.0.10 with default parameters; sequencing adapters were not removed at this stage. Following demultiplexing, reads were processed using BBMap’s v38.46 (available from https://sourceforge.net/projects/bbmap/) clumpify.sh script to remove optical duplicates (parameters dupedist=40 dedupe=t optical=t spany=t adjacent=t). Quality control of the reads was performed using fastp v0.20.0 [35] and included trimming of sequencing adapters and window-based quality trimming; parameters were as follows: --trim_front1 5 --trim_front2 5 --trim_tail1 1 --trim_tail2 1 --cut_right --cut_right_window_size 4 --cut_right_mean_quality 18 --trim_poly_x --poly_x_min_len 10 --n_base_limit 0 --low_complexity_filter --length_required 75. Relevant statistics, such as base content and PCR duplication levels, were extracted from fastp’s json output files. For fecal samples, human genomic reads were identified and removed with BMTagger v3.101 [36] using human genome assembly GRCh38 as reference. For the MOSAIC Standards Challenge, publicly available data in the January 2020 data release were downloaded (see “MOSAIC Standards Challenge samples and public sequencing data”) and processed as described above, unless stated otherwise in Table S7.

For the mock communities, quantification of relative abundances was performed by pseudo-mapping using kallisto v0.46.1 [27] with default settings, using the chromosomal sequences of each strain as references, based on its near-perfect accuracy as evaluated using in silico generated data (Fig. S26). Transcripts per million estimates generated by kallisto were used for downstream analysis. Reads were also aligned to the reference genome sequences using bowtie2 v2.4.1 [37], specifying options --no-unal --no-mixed --no-discordant. Generated SAM files were processed using samtools v1.10 [38], and fragment size distributions and base call error profiles generated using BBmaps’s reformat.sh script. Metagenome assembly was performed by MEGAHIT v1.2.9 [39] with default settings, and summary statistics for the assemblies generated using QUAST v5.0.0 [40].

For the fecal samples, reads were annotated by kraken2 v2.0.8 [41] against the GTDB_r89_54k_kraken2 database [42], specifying options --confidence 0.05 --paired. The proportion of reads assigned to each species was used as a proxy for taxonomic profiles. Alternatively, mOTUs2 v2.5.1 [43] was used to generate OTU-level taxonomic profiles, with default settings based on the default 10 single-copy marker genes. If necessary, zero relative abundances were set to 0.001% for compositional data analysis (see below).

### Data analysis

All data were imported into R v4.0.2 [44] for analysis and visualization, using dplyr v1.0.2 [45] for data handling and ggplot2 v3.3.2 [46] for visualization; other R packages used are referenced as appropriate below.

#### Calculation of compositional means, metric variances, and Aitchison distances

Microbiome community compositions are given by a vector ***x*** = [ *x*_*1*_, …, *x*_*D*_ ] of *D* strictly non-negative elements representing the abundances of each part (species, genes, …) in the community, subject to a total sum constraint.

Following standard concepts and definitions [47, 48], the central tendency (center or compositional mean) of a compositional data set **X** = [ ***x***_*1*_, …, ***x***_*n*_ ], where ***x***_*j*_ = [ *x*_*1,j*_, …, *x*_*D,j*_ ] represents one of *n* individual compositions, was calculated as the closed geometric mean:

$$ \mathrm{cen}\left(\mathbf{X}\right)=\mathrm{clo}\left[\ {g}_1,\dots, {g}_D\ \right] $$

where *g*_*i*_ is the geometric mean of the abundance of part *i* across the *n* compositions and clo represents the closure operation:

$$ \mathrm{clo}\left(\boldsymbol{x}\right)=\kappa \bullet \left[\ \frac{x_1}{\sum_{i=1}^D{x}_D},\dots, \frac{x_D}{\sum_{i=1}^D{x}_D}\ \right] $$

where κ is the closure constant, usually set to 1 or 100%.

Dispersion of a compositional data set **X** is known as the metric (or total) variance, denoted as mvar(**X**), and can be calculated based on the variation matrix, denoted as varmat(**X**), of all possible logratio variances:

$$ \mathrm{varmat}\left(\mathbf{X}\right)=\left(\begin{array}{ccc}\operatorname{var}\left(\ln \frac{x_1}{x_1}\right)& \cdots & \operatorname{var}\left(\ln \frac{x_1}{x_D}\right)\\ {}\vdots & \ddots & \vdots \\ {}\operatorname{var}\left(\ln \frac{x_D}{x_1}\right)& \cdots & \operatorname{var}\left(\ln \frac{x_D}{x_D}\right)\end{array}\right) $$

$$ \mathrm{mvar}\left(\mathbf{X}\right)=\frac{1}{2D}{\sum}_{i=1}^D{\sum}_{j=1}^D\operatorname{var}\left(\ln \frac{x_i}{x_j}\right) $$

For calculation, we used the functions *variation* and *mvar* in the R package compositions v2.0 [49] to obtain variation matrices and metric variances, respectively. Based on the variation matrix, we also calculated the contribution of each logratio variance to the metric variance.

The distance between two compositions ***x*** = [ *x*_*1*_, …, *x*_*D*_ ] and ***y*** = [ *y*_*1*_, …, *y*_*D*_ ] is known as the Aitchison distance (*d*_*A*_), calculated as:

$$ {\mathrm{d}}_{\mathrm{A}}\left(\boldsymbol{x},\boldsymbol{y}\right)=\kern0.5em \sqrt{\frac{1}{2D}\sum \limits_{i=1}^D\sum \limits_{j=1}^D{\left[\ln \frac{x_i}{x_j}-\ln \frac{y_i}{y_j}\right]}^2} $$

This is equivalent to the Euclidean distance after centered log ratio (clr) transformation:

$$ \mathrm{clr}\left(\boldsymbol{x}\right)=\kern0.5em \left(\ln \frac{x_1}{\mathrm{g}\left(\boldsymbol{x}\right)},\dots, \ln \frac{x_D}{\mathrm{g}\left(\boldsymbol{x}\right)}\right) $$

where g(***x***) is the geometric mean of the abundances across parts of ***x*.** Accordingly, compositional principal component analysis (PCA) was performed using R’s stats *prcomp* function, after clr transformation of the abundance data.

#### Performance metrics

Following ISO Standard 5725, accuracy of analytical measurements and methods is defined as the closeness of agreement of results to the accepted reference value and consists of two components, namely trueness and precision [20]. Trueness reflects the closeness of agreement between the average of a large series of measurements and the reference value. Precision reflects the variability of measurement results and is evaluated at three levels, namely repeatability, intermediate precision, and interlaboratory reproducibility.

No consensus currently exists on how to quantify the accuracy of microbiome community measurement results [15]. Recently, compositionality-aware methods have been advocated as the statistically valid approach for analysis of sequencing data [50]. In compositional data analysis, distances between compositions are expressed in terms of Aitchison distance (see definition above) and the metric variance (see definition above) captures total logratio variances. These metrics may however be difficult to interpret and communicate with intended users of our SOPs. Therefore, we defined additional metrics that may be more intuitive and easier to interpret.

More specifically, we computed the closeness of agreement between two compositions ***x*** and ***y*** (for example, measured and ground truth compositions) as the geometric mean of the part-wise absolute fold-differences, denoted as gmAFD:

$$ \mathrm{gmAFD}\left(\boldsymbol{x},\boldsymbol{y}\right)={\left(\prod \limits_{i=1}^D{e}^{\left|\ln \frac{x_i}{y_i}\right|}\right)}^{\frac{1}{D}} $$

Here, accuracy was determined as the gmAFD of individual measurement results to the ground truth and trueness as the gmAFD of the compositional mean of replicated measurements to the ground truth. We note that while the gmAFD is not a compositional metric, it is more intuitive and thus easier to interpret. Further, within the scope of our data, gmAFD values were strongly correlated with Aitchison distances (Fig. S27).

To express variability of measured compositions, we calculated the quadratic mean of the part-wise coefficients of variation of measured abundances, denoted as qmCV:

$$ \mathrm{qmCV}\left(\mathbf{X}\right)={\left(\frac{\sum_{i=1}^D{\left({CV}_i\right)}^2}{D}\right)}^{1/2} $$

where *CV*_*i*_ represents the coefficient of variation of the relative abundance of part *i*. As for the gmAFD, although qmCV does not consider compositionality of the data, we found that, for sufficiently small variances, qmCV was proportional to the square root of the metric variance, with a proportionality constant of *D*^−1/2^ (Fig. S27).

#### Summarizing quantification bias due to genomic GC content and fragmentation bias

To quantify GC bias, log_2_-transformed fold-differences in measured abundances for each pair of strains in the DNA mock community were regressed to their corresponding differences in genomic GC content, using an intercept-free linear model. The slope of the linear regression model was interpreted as an overall measure of GC bias, with negative slopes indicating overrepresentation of lower-GC genomes or strains. To summarize fragmentation bias, Aitchison distances between observed and expected base frequencies were calculated for individual positions in the reads and subsequently averaged. Expected base frequencies for the DNA mock community were calculated from the reference genome sequences.

#### Calculation of the limit of detection and limit of quantification

The limit of detection (LOD) was determined by binomial regression of species-wise probabilities of detection to the species-wise mean relative abundance (log-transformed) using a generalized linear model with complementary log-log (cloglog) link function. Fitting was performed using the function glm in R’s stats package. The LOD was defined as the fitted mean species relative abundance corresponding to a fitted POD of 95%. In a similar fashion, the limit of quantification (LOQ) was calculated by regressing species-wise coefficients of variation of relative abundances to the species-wise mean relative abundance with a negative exponential curve [28], with the modification that the mean coefficient of variation of the top 10% most abundant species was imposed as a lower plateau. Fitting was performed by nonlinear least squares regression using the function nls in R’s stats package. The LOQ was defined as the fitted mean species relative abundance corresponding to a fitted coefficient of variation of 40%.

#### Distance-based analysis of variance

For analysis of the intermediate precision and interlaboratory reproducibility studies, we used distance-based analysis of variance using a traditional one-way random effects model. To obtain metric variances, between- and within-group total sums of squares (TSS_b_ and TSS_w_, respectively) were calculated based on the squared Aitchison distances:

$$ {TSS}_b=n\bullet \sum \limits_{i=1}^p{\mathrm{d}}_{\mathrm{A}}^2\left(\mathrm{cen}\left({\mathbf{X}}_i\right),\mathrm{cen}\left({\mathbf{X}}_{ij}\right)\right) $$

$$ {TSS}_w=\sum \limits_{j=1}^n\sum \limits_{i=1}^p{\mathrm{d}}_{\mathrm{A}}^2\left({\mathbf{x}}_{ij},\mathrm{cen}\left({\mathbf{X}}_i\right)\right) $$

where *n* is the number of replicates within a group, *p* is the number of groups (that is, lot+operator combinations and laboratories for assessment of intermediate precision and interlaboratory reproducibility, respectively), cen(**X**_*i*_) and cen(**X**_*ij*_) are the group and grand means (centers), respectively, and **x**_*ij*_ is measurement *j* performed by group *i*. All subsequent calculations followed standard ANOVA procedures [51], as detailed in the Supplementary Methods. For simplicity, we used the function adonis in the R package vegan v2.5 [52] to calculate the required sums of squares, using the Euclidean distance based on clr transformed abundance data. Finally, obtained variance components were converted to approximate qmCVs based on the proportionality between metric variances and qmCVs (see above).

## Supplementary Information

**Additional file 1.**

## Data Availability

Genome sequences generated in this study are available in the DDBJ/EMBL/GenBank database (see Table S1 for accession numbers). All raw sequencing data have been deposited in NCBI’s Sequence Read Archive repository under BioProject PRJNA650228 (see Table S8 for accession numbers). Sequencing data generated as part of the MOSAIC Standards Challenge were also uploaded to the Standards Challenge website (URL: https://platform.mosaicbiome.com/challenges/8).

## References

[1] Gilbert JA, Blaser MJ, Caporaso JG, Jansson JK, Lynch SV, Knight R (2018). Current understanding of the human microbiome. Nat Med..

[2] Shreiner AB, Kao JY, Young VB (2015). The gut microbiome in health and in disease. Curr Opin Gastroenterol..

[3] Schmidt TSB, Raes J, Bork P (2018). The human gut microbiome: From association to modulation. Cell..

[4] Fong W, Li Q, Yu J (2020). Gut microbiota modulation: a novel strategy for prevention and treatment of colorectal cancer. Oncogene..

[5] Quigley EMM, Gajula P (2020). Recent advances in modulating the microbiome. F1000Res.

[6] Choo JM, Leong LE, Rogers GB (2015). Sample storage conditions significantly influence faecal microbiome profiles. Sci Rep..

[7] Watson EJ, Giles J, Scherer BL, Blatchford P (2019). Human faecal collection methods demonstrate a bias in microbiome composition by cell wall structure. Sci Rep..

[8] Lim MY, Song EJ, Kim SH, Lee J, Nam YD (2018). Comparison of DNA extraction methods for human gut microbial community profiling. Syst Appl Microbiol..

[9] Yang F, Sun J, Luo H, Ren H, Zhou H, Lin Y, Han M, Chen B, Liao H, Brix S, Li J, Yang H, Kristiansen K, Zhong H (2020). Assessment of fecal DNA extraction protocols for metagenomic studies. Gigascience.

[10] Clooney AG, Fouhy F, Sleator RD, O’ Driscoll A, Stanton C, Cotter PD, Claesson MJ (2016). Comparing apples and oranges?: Next generation sequencing and its impact on microbiome analysis. Plos One.

[11] Ye SH, Siddle KJ, Park DJ, Sabeti PC (2019). Benchmarking metagenomics tools for taxonomic classification. Cell..

[12] Sinha R, Abu-Ali G, Vogtmann E, Fodor AA, Ren B, Amir A, Schwager E, Crabtree J, Ma S, Abnet CC, Knight R, White O, Huttenhower C, Microbiome Quality Control Project Consortium (2017). Assessment of variation in microbial community amplicon sequencing by the Microbiome Quality Control (MBQC) project consortium. Nat Biotechnol..

[13] Han D, Gao P, Li R, Tan P, Xie J, Zhang R, Li J (2020). Multicenter assessment of microbial community profiling using 16S rRNA gene sequencing and shotgun metagenomic sequencing. J Adv Res..

[14] Stulberg E, Fravel D, Proctor LM, Murray DM, LoTempio J, Chrisey L, Garland J, Goodwin K, Graber J, Harris MC, Jackson S, Mishkind M, Porterfield DM, Records A (2016). An assessment of US microbiome research. Nat Microbiol..

[15] Amos GCA, Logan A, Anwar S, Fritzsche M, Mate R, Bleazard T, Rijpkema S (2020). Developing standards for the microbiome field. Microbiome..

[16] Kim D, Hofstaedter CE, Zhao C, Mattei L, Tanes C, Clarke E, Lauder A, Sherrill-Mix S, Chehoud C, Kelsen J, Conrad M, Collman RG, Baldassano R, Bushman FD, Bittinger K (2017). Optimizing methods and dodging pitfalls in microbiome research. Microbiome..

[17] Knight R, Vrbanac A, Taylor BC, Aksenov A, Callewaert C, Debelius J, Gonzalez A, Kosciolek T, McCall LI, McDonald D, Melnik AV, Morton JT, Navas J, Quinn RA, Sanders JG, Swafford AD, Thompson LR, Tripathi A, Xu ZZ, Zaneveld JR, Zhu Q, Caporaso JG, Dorrestein PC (2018). Best practices for analysing microbiomes. Nat Rev Microbiol..

[18] Costea PI, Zeller G, Sunagawa S, Pelletier E, Alberti A, Levenez F, Tramontano M, Driessen M, Hercog R, Jung FE, Kultima JR, Hayward MR, Coelho LP, Allen-Vercoe E, Bertrand L, Blaut M, JRM B, Carton T, Cools-Portier S, Daigneault M, Derrien M, Druesne A, de Vos WM, Finlay BB, Flint HJ, Guarner F, Hattori M, Heilig H, Luna RA, van Hylckama Vlieg J, Junick J, Klymiuk I, Langella P, Le Chatelier E, Mai V, Manichanh C, Martin JC, Mery C, Morita H, O’Toole PW, Orvain C, Patil KR, Penders J, Persson S, Pons N, Popova M, Salonen A, Saulnier D, Scott KP, Singh B, Slezak K, Veiga P, Versalovic J, Zhao L, Zoetendal EG, Ehrlich SD, Dore J, Bork P (2017). Towards standards for human fecal sample processing in metagenomic studies. Nat Biotechnol.

[19] Jackson SA (2019). The MOSAIC Standards Challenge: capturing the extent and sources of variability in NGS-based microbiome profiling protocols and analyses. J Biomol Tech..

[20] Menditto A, Patriarca P, Magnusson B (2017). Understanding the meaning of accuracy, trueness and precision. Accredit Qual Assur..

[21] Song Z, Schlatter D, Gohl DM, Kinkel LL (2018). Run-to-run sequencing variation can introduce taxon-specific bias in the evaluation of fungal microbiomes. Phytobiomes J..

[22] Nishijima S, Suda W, Oshima K, Kim SW, Hirose Y, Morita H, Hattori M (2016). The gut microbiome of healthy Japanese and its microbial and functional uniqueness. DNA Res..

[23] Sakon H, Nagai F, Morotomi M, Tanaka R (2008). *Sutterella parvirubra* sp. nov. and *Megamonas funiformis* sp. nov., isolated from human faeces. Int J Syst Evol Microbiol..

[24] Takagi T, Naito Y, Inoue R, Kashiwagi S, Uchiyama K, Mizushima K, Tsuchiya S, Dohi O, Yoshida N, Kamada K, Ishikawa T, Handa O, Konishi H, Okuda K, Tsujimoto Y, Ohnogi H, Itoh Y (2019). Differences in gut microbiota associated with age, sex, and stool consistency in healthy Japanese subjects. J Gastroenterol..

[25] Watanabe S, Kameoka S, Shinozaki NO, Kubo R, Nishida A, Kuriyama M, Takeda AK. A cross-sectional analysis from the Mykinso cohort study: establishing reference ranges for Japanese gut microbial indices. Bioscience of Microbiota, Food and Health. Advance publication available at doi: 10.12938/bmfh.2020-038, 2021. 10.12938/bmfh.2020-038 PMC8099632 33996369

[26] de Bruin OM, Birnboim HC (2016). A method for assessing efficiency of bacterial cell disruption and DNA release. BMC Microbiol..

[27] Bray NL, Pimentel H, Melsted P, Pachter L (2016). Near-optimal probabilistic RNA-seq quantification. Nat Biotechnol..

[28] Barlow JT, Bogatyrev SR, Ismagilov RF (2020). A quantitative sequencing framework for absolute abundance measurements of mucosal and lumenal microbial communities. Nat Commun..

[29] Poulsen CS, Pamp SJ, Ekstrøm CT, Aarestrup FM. Library preparation and sequencing platform introduce bias in metagenomics characterisation of microbial communities. Preprint available at https://www.biorxiv.org/content/10.1101/592154v1. Accessed 1 Mar 2021.

[30] Sato MP, Ogura Y, Nakamura K, Nishida R, Gotoh Y, Hayashi M, et al. Comparison of the sequencing bias of currently available library preparation kits for Illumina sequencing of bacterial genomes and metagenomes. DNA Res. 2019;26(5):391–8. 10.1093/dnares/dsz017. 10.1093/dnares/dsz017 PMC6796507 31364694

[31] Aird D, Ross MG, Chen WS, Danielsson M, Fennell T, Russ C, Jaffe DB, Nusbaum C, Gnirke A (2011). Analyzing and minimizing PCR amplification bias in Illumina sequencing libraries. Genome Biol..

[32] Jones MB, Highlander SK, Anderson EL, Li W, Dayrit M, Klitgord N, Fabani MM, Seguritan V, Green J, Pride DT, Yooseph S, Biggs W, Nelson KE, Venter JC (2015). Library preparation methodology can influence genomic and functional predictions in human microbiome research. Proc Natl Acad Sci USA..

[33] Browne PD, Nielsen TK, Kot W, Aggerholm A, Gilbert MTP, Puetz L, Rasmussen M, Zervas A, Hansen LH (2020). GC bias affects genomic and metagenomic reconstructions, underrepresenting GC-poor organisms. Gigascience.

[34] Zhang B, Brock M, Arana C, Dende C, Hooper L, Raj P. Impact of bead-beating intensity on microbiome recovery in mouse and human stool: Optimization of DNA extraction. Preprint available at https://www.biorxiv.org/content/10.1101/2020.06.15.151753v1. Accessed 1 Dec 2020.

[35] Chen S, Zhou Y, Chen Y, Gu J (2018). fastp: an ultra-fast all-in-one FASTQ preprocessor. Bioinformatics..

[36] Rotmistrovsky K, Agarwala R. 2011. BMTagger: best match tagger for removing human reads from metagenomics datasets. Available at ftp://ftp.ncbi.nlm.nih.gov/pub/agarwala/bmtagger/. Downloaded on March 24, 2020.

[37] Langmead B, Salzberg SL (2012). Fast gapped-read alignment with Bowtie 2. Nat Methods..

[38] Li H, Handsaker B, Wysoker A, Fennell T, Ruan J, Homer N, Marth G, Abecasis G, Durbin R (2009). 1000 genome project data processing subgroup. The sequence alignment/map format and SAMtools. Bioinformatics..

[39] Li D, Liu CM, Luo R, Sadakane K, Lam TW (2015). MEGAHIT: an ultra-fast single-node solution for large and complex metagenomics assembly via succinct de Bruijn graph. Bioinformatics..

[40] Gurevich A, Saveliev V, Vyahhi N, Tesler G (2013). QUAST: quality assessment tool for genome assemblies. Bioinformatics..

[41] Wood DE, Salzberg SL (2014). Kraken: ultrafast metagenomic sequence classification using exact alignments. Genome Biol..

[42] Méric G, Wick RR, Watts SC, Holt KE, Inouye M. Correcting index databases improves metagenomic studies. Preprint available at https://www.biorxiv.org/content/10.1101/712166v1. Accessed 1 Dec 2020.

[43] Milanese A, Mende DR, Paoli L, Salazar G, Ruscheweyh HJ, Cuenca M, Hingamp P, Alves R, Costea PI, Coelho LP, Schmidt TSB, Almeida A, Mitchell AL, Finn RD, Huerta-Cepas J, Bork P, Zeller G, Sunagawa S (2019). Microbial abundance, activity and population genomic profiling with mOTUs2. Nat Commun..

[44] Core Team R (2013). R: a language and environment for statistical computing.

[45] Wickham H, François R, Henry L, Müller K. 2020. dplyr: a grammar of data manipulation. R package version 1.0.2. URL: https://CRAN.R-project.org/package=dplyr. Accessed 19 Aug 2020.

[46] Wickham H (2016). ggplot2: Elegant graphics for data analysis.

[47] Pawlowsky-Glahn V, Egozcue JJ, Tolosano-Delgado R (2007). Lecture notes on compositional data analysis.

[48] Aitchison J (1986). The statistical analysis of compositional data.

[49] van den Boogaart KG, Tolosana-Delgado R, Bren M. 2020. compositions: compositional data analysis. R package version 2.0-0. URL: https://CRAN.R-project.org/package=compositions. Accessed 15 July 2020.

[50] Gloor GB, Macklaim JM, Pawlowsky-Glahn V, Egozcue JJ (2017). Microbiome datasets are compositional: and this is not optional. Front Microbiol..

[51] Kuttatharmmakul S, Massart DL, Smeyers-Verbeke J (1999). Comparison of alternative measurement methods. Anal Chim Acta..

[52] Oksanen J, Guillaume Blanchet F, Friendly M, Kindt R, Legendre P, McGlinn D, Minchin PR, O’Hara RB, Simpson GL, Solymos P, Stevens MHH, Szoecs E, Wagner H. 2020. vegan: community ecology package. R package version 2.5-7. URL: https://CRAN.R-project.org/package=vegan. Accessed 29 Nov 2020.

