# Supplementary material for: Validation and standardization of DNA extraction and library construction methods for metagenomics-based human fecal microbiome measurements
Source: Microbiome. 2021 Apr 29;9:95. doi: 10.1186/s40168-021-01048-3 (PMC8082873; doi:10.1186/s40168-021-01048-3)
Supplement: Supplementary file 2 — Additional file 1. [file 40168_2021_1048_MOESM1_ESM.pdf]

— Supplementary Information —

Validation and standardization of DNA extraction and library construction methods for metagenomics-based human fecal microbiome measurements

Content

Supplementary Methods — p. 2

Supplementary Figures — p. 8

Supplementary Tables — p. 38

Supplementary References — p. 54

Supplementary Methods

Genome sequencing and assembly

For *Escherichia coli* strain NBRC 3301, *Akkermansia muciniphila* strain JCM 30893 and *Faecalibacterium prausnitzii* strain JCM 31915, extraction of DNA, sequencing library construction, sequencing and genome assembly were performed as described by Tourlousse *et al.* (2020a, 2020b, 2020c, 2020d). Genome coverage of the short- and long-read libraries were in the range of 100–210× and 45–120×, respectively.

For *Cutibacterium acnes* subsp. *acnes* strain NBRC 107605^T, *Bacillus subtilis* subsp. *subtilis* strain NBRC 13719^T, *Streptococcus mutans* strain NBRC 13955^T, *Lactobacillus delbrueckii* subsp. *delbrueckii* strain NBRC 3202^T, short-read libraries were prepared using the TruSeq DNA PCR-Free kit and sequencing performed on a MiSeq instrument. For *Staphylococcus epidermidis* strain NBRC 100911^T, the TruSeq DNA Nano kit was used for library construction and sequencing performed using a HiSeq instrument. Quality control of Illumina short reads was performed using CLC Genomic Workbench v8.5.1 (Qiagen). For all above strains, long-read libraries were generated with a Ligation Sequencing Kit (Oxford Nanopore technologies) and a GridION device used for sequencing. Combined genome coverage of the short- and long-read libraries exceeded 500× for all strains. Genome assembly was performed using Unicycler v0.4.4 (Wick *et al.*, 2017).

For *Bacteroides uniformis* strain NBRC 113350 and *Enterocloster clostridioforme* strain NBRC 113352, short-read libraries were prepared using the TruSeq DNA PCR-Free kit and sequencing performed on a MiSeq instrument. For *Bacteroides uniformis* strain NBRC 113350, long-read libraries were prepared using the SMRTBell Template Prep Kit 1.0 (PacBio) and a PacBio SMRT machine used for sequencing. For *Enterocloster clostridioforme* strain NBRC 113352, long-read libraries were generated with a Ligation Sequencing Kit (Oxford Nanopore technologies) and a GridION instrument used for sequencing. Quality control of Illumina short reads was performed using CLC Genomic Workbench v8.5.1 (Qiagen). Genome coverages were 140× and 340× for strains NBRC 113350 and NBRC 113352, respectively. Genome assembly was performed using Canu v1.7.1 (Koren *et al.*, 2017) and Unicycler v0.4.4.

Quality of all genomes was confirmed by CheckM v1.0.18 (Parks *et al.*, 2015) based on genome completeness, contamination, and coding density.

Description of protocol P

Protocol P is an in-house phenol-chloroform based method for DNA extraction and was performed as follows. Fecal sample was resuspended in 300 μl of 100 mM NaH_2PO_4 (pH 8.0), 300 μl of lysis buffer (100 mM of NaCl, 500 mM of Tris-HCl, 10% of sodium dodecyl sulfate, pH 8.0) and 300 μl of phenol-chloroform-isoamyl alcohol

(PCI, 25:24:1, v:v:v), and 1.2 g of 0.1-mm autoclaved Zirconia beads added. Cell lysis was performed by bead-beating using the FastPrep-24 instrument for 40 s or 60 s at a speed of 6 m/s. Following incubation for 10 min at 60 °C, the sample was centrifuged for 5 min at 14,000 ×*g* and 600 μl of supernatant recovered. If applicable, 0.01 volumes of RNase A (10 mg/ml) were added, and RNA digested for 10 min at 37 °C. An equal volume of PCI was then added, the sample mixed by vortexing, centrifuged for 3 min and the supernatant recovered. These steps were repeated two times. Subsequently, an equal volume of chloroform-isoamyl alcohol (24:1, v/v) was added, the sample vigorously mixed by vortexing and the aqueous phase recovered after centrifugation. Precipitation of DNA was performed by addition of 0.1 volumes of 3 M sodium acetate (pH 5.2) and an equal volume of isopropanol, followed by centrifugation for 30 min at 4 °C. The DNA pellet was washed with 70% ethanol, air dried and dissolved in EB buffer (Qiagen).

Distance-based analysis of variance

To obtain estimates of intermediate precision and interlaboratory reproducibility, we performed distance-based analysis of variance (ANOVA), as outlined below. Note that in the following example, an interlaboratory study is considered but an identical approach applies to the assessment of intermediate precision with a single factor (as performed in this study).

Consider an interlaboratory study in which a single sample is analyzed by p laboratories, each performing n measurements under repeatability conditions. Note that in our study, each laboratory performed the same number of measurements such that the ANOVA model was balanced. As for traditional univariate ANOVA, variance components were obtained based on the within- and between- group sums of squares, with the difference that the Aitchison distance ($\mathrm{d_A}$) was used to obtain the required sum of squares.

More specifically, the within- and between-group total sums of squares (denoted by $\mathrm{TSS_w}$ and $\mathrm{TSS_b}$) were obtained as follows:

$$\mathrm{TSS_w} = \sum_{j=1}^{n}\sum_{i=1}^{p} \mathrm{d_A^2}\left(\mathrm{x_{ij}}, \mathrm{cen(X_i)}\right)$$

$$\mathrm{TSS_b} = \mathrm{n} \cdot \sum_{i=1}^{p} \mathrm{d_A^2}\left(\mathrm{cen(X_i)}, \mathrm{cen}\left(\mathrm{X_{ij}}\right)\right)$$

where $\mathrm{x_{ij}}$ denotes individual measurement result j of laboratory i, $\mathrm{cen(X_i)}$ is the compositional mean (group mean) of all measurements results of laboratory i, and $\mathrm{cen}\left(\mathrm{X_{ij}}\right)$ is the compositional mean (grand mean) of all measurement results.

Based on the degrees of freedom for each of the total sum of squares, the mean sums of squares (MSS) were then obtained as:

$$\mathrm{MSS_w} = \frac{\mathrm{TSS_w}}{\mathrm{p(n-1)}}$$

$$\mathrm{MSS_b} = \frac{\mathrm{TSS_b}}{\mathrm{p-1}}$$

with expected values of the mean sums of squares given by:

$$\mathrm{E(MSS_w)} = \sigma_r^2$$

$$\mathrm{E(MSS_b)} = \sigma_r^2 + \mathrm{n} \cdot \sigma_L^2$$

where σ_r^2 is the residual (that is, technical) population variance and σ_L^2 is the population variance due to varying laboratories. The repeatability sample variance s_r^2, laboratory sample variance s_L^2, and reproducibility sample variance s_R^2 were then obtained based on the mean sums of squares as follows:

$$s_r^2 = \mathrm{MSS_w}$$

$$s_L^2 = \left(\frac{1}{n}\right)\mathrm{MSS_b} - \left(\frac{1}{n}\right)\mathrm{MSS_w}$$

$$s_R^2 = s_r^2 + s_L^2 = \left(\frac{1}{n}\right)\mathrm{MSS_b} + \left(\frac{n-1}{n}\right)\mathrm{MSS_w}$$

or, in terms of metric variances:

$$\mathrm{mvar_r} = \mathrm{MSS_w}$$

$$\mathrm{mvar_L} = \left(\frac{1}{n}\right)\mathrm{MSS_b} - \left(\frac{1}{n}\right)\mathrm{MSS_w}$$

$$\mathrm{mvar_R} = \mathrm{mvar_r} + \mathrm{mvar_L} = \left(\frac{1}{n}\right)\mathrm{MSS_b} + \left(\frac{n-1}{n}\right)\mathrm{MSS_w}$$

Confidence intervals for the estimates of the variances were calculated using the critical values of the chi-square distribution χ_ν^2 with the appropriate degrees of freedom ν. For $\mathrm{mvar_R}$, effective degrees of freedom ν_R were obtained using the Welch-Satterthwaite equation (Satterthwaite, 1946; Welch, 1947):

$$\nu_R = \frac{(\mathrm{mvar}_R)^2}{\frac{(\mathrm{MSS}_b/n)^2}{p-1} + \frac{\left((n-1)\mathrm{MSS}_w/n\right)^2}{p(n-1)}}$$

Confidential intervals for the variances are then given by:

$$\mathrm{mvar}_r \frac{\nu}{\chi^2_{\nu_r,1-\alpha/2}} \le \sigma_r^2 \le \mathrm{mvar}_r \frac{\nu}{\chi^2_{\nu_r,\frac{\alpha}{2}}}$$

$$\mathrm{mvar}_R \frac{\nu}{\chi^2_{\nu_R,1-\alpha/2}} \le \sigma_R^2 \le \mathrm{mvar}_R \frac{\nu}{\chi^2_{\nu_R,\frac{\alpha}{2}}}$$

Setting acceptable levels of error for trueness/accuracy

To guide future development of SOPs and routine quality management, we set target values for the acceptable level of errors for metagenomics-based measurement of the mock community. Here, error refers to the differences between measured compositions and the "ground truth", determined based on DNA quantification by fluorometry for the DNA mock community and total DNA content quantification by acid-catalyzed depurination and quantification of released adenine content by HPLC, as described in the Methods, for the cell mock community. In short, we used simulations to account for the "uncertainty" in value assignments for the "ground truth" and metagenomics-based measurements. The acceptable level of error was then computed as the 95th percentile of the differences between both sets of simulated compositions, as detailed below:

DNA mock community. The acceptable level of error for the DNA mock community was set by considering differences between the "ground truth" and values determined by metagenome sequencing of libraries constructed by PCR-free methods using physical DNA fragmentation (protocols A0, C0, D0 and E0 in Table S2).

For the DNA fluorometric measurements, a type B uncertainty in the value assignments was considered, by assuming that measured DNA concentrations were normally distributed around the mean of replicated fluorometric measurements, with a standard deviation of 0.25 (equivalent to a coefficient of variation of 5%). Based on the "ground truth" value and type B uncertainty, 10,000 values (that is, DNA concentrations) were simulated for 20 strains using the function *rnorm* in R's stat package, simulated values combined and normalized to 100%.

For the metagenome sequencing based measurements, the closed geometric mean of measurements performed by each of the protocols (averaged across three technical replicates) was assigned as value. A type A uncertainty was then considered based on the variance matrix of the means across replicates for each protocol, computed using the

function *var.acomp* function in the R package compositions (van den Boogaart *et al.*, 2020). Based on the mean and variance matrix (considering only its diagonal), we then simulated 10,000 random compositions using *rnorm.acomp*.

We then calculated the geometric mean and maximum of absolute fold differences (AFD) between 10,000 pairs from the above two simulated compositions. Finally, the acceptable level of error was defined as the 95th percentile of the 10,000 mean and maximum values of the AFDs.

R code for these calculations is provided below:

```
# simulation of 10,000 compositions considering the type B uncertainty in the measured DNA concentrations #

simulatedB <- NULL

for(i in strains) {
  simulatedB <- rbind(simulatedB,
                  data.frame(strainID = i,  value = rnorm(10^4,5,0.25)) %>%
                    dplyr::mutate(id = paste0("sim", 1:n())))
}

simulatedB <- simulatedB %>%
  dplyr::group_by(id) %>%
  dplyr::mutate(value = value / sum(value)) %>%
  reshape2::dcast(id ~ strainID, value.var = "value") %>%
   dplyr::select(-id)

# simulation of 10,000 compositions considering the type A uncertainty in the measured compositions using protocols A0, C0, D0 and E0 #

## calculation of per-protocol closed geometric centres ##

centres <- df %>%
  dplyr::group_by(protocolID, strainID) %>%
  dplyr::summarise(abundanceMeasured = exp(mean(log(abundanceMeasured)))) %>%
  dplyr::ungroup() %>%
  dplyr::group_by(protocolID) %>%
  dplyr::mutate(abundanceMeasured = abundanceMeasured / sum(abundanceMeasured)) %>%
  reshape2::dcast(protocolID ~ strainID, value.var = "abundanceMeasured") %>%
   dplyr::select(-protocolID)

## calculation per-protocol compositional centres ##

m <- apply(centers, 2, function(x) exp(mean(log(x))))
m <- m / sum(m)

## calculation co-variance matrix of centres for simulations, only using diagonals ##

v <- diag(diag(var.acomp(acomp(centres))))

## simulation of 10,000 compositions ##

simulatedA <- compositions::rnorm.acomp(10^4, m, v)

## random selection of compositions from simulatedB and simulatedA and calculation of the geometric mean and maximum of absolute fold differences ##

dist1 <- vector()
dist2 <- vector()

for(i in 1:10^4) {
  tmp1 <- as.vector(sims[i])
  tmp2 <- unname(unlist(refs[i,]))
   dist1 <- append(dist1,PKNCA::geomean(exp(abs(log(tmp1/tmp2)))))
  dist2 <- append(dist2,max(exp(abs(log(tmp1/tmp2)))))
}

## setting acceptable levels based on 95% percentiles ##

round(quantile(dist1, probs = c(95/100)),2)
round(quantile(dist2, probs = c(95/100)),2)
```

Cell mock community. For the cell mock community, the acceptable level of error was set by considering differences between the "ground truth" and values determined by metagenome sequencing of libraries constructed

from DNA extracted by protocols P and Q, followed by library construction using protocol BL. For the HPLC measurements, a 10% type A uncertainty was considered.

Supplementary Figures

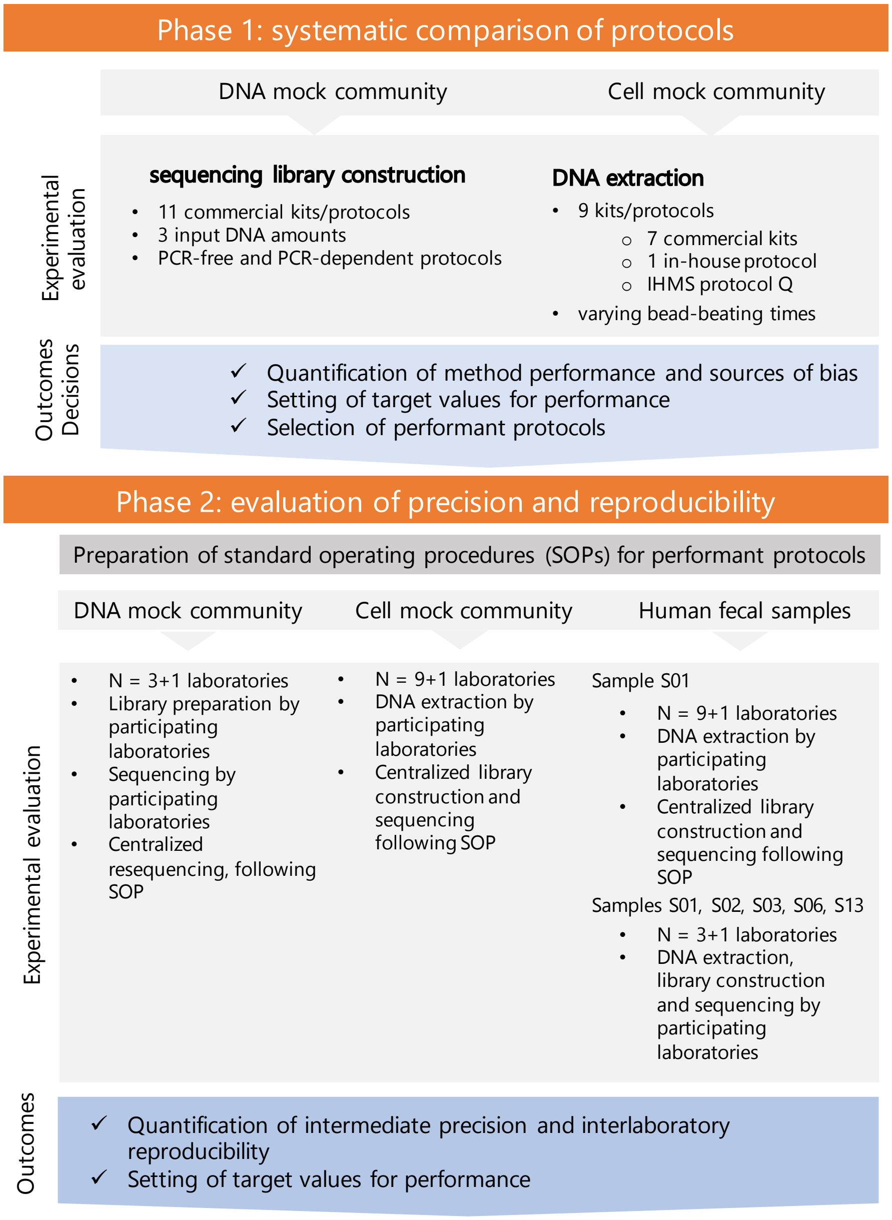

Supplementary Figure S1. Schematic of the design of this study. In the first phase, a wide range of protocols for DNA extraction and library construction were evaluated using defined mock communities, leading to: (i) ranking of protocols based on analytical performance (that is, agreement to the "ground truth" values) and considerations related to hands-on time, cost, *etc.*, (ii) identification of main sources of quantitative bias, and (iii) establishment of target values for key performance metrics. In the second phase, SOPs were established for highly performant protocols and evaluated with respect to variability of measurement results within a single laboratory (that is, intermediate precision) and across multiple laboratories (interlaboratory reproducibility), using mock communities and human fecal samples, leading to (i) quantification of measurement variability and (ii) establishment of target values for key performance metrics. Detailed experimental schemes for assessment of intermediate precision and interlaboratory reproducibility are provided in Fig. S13.

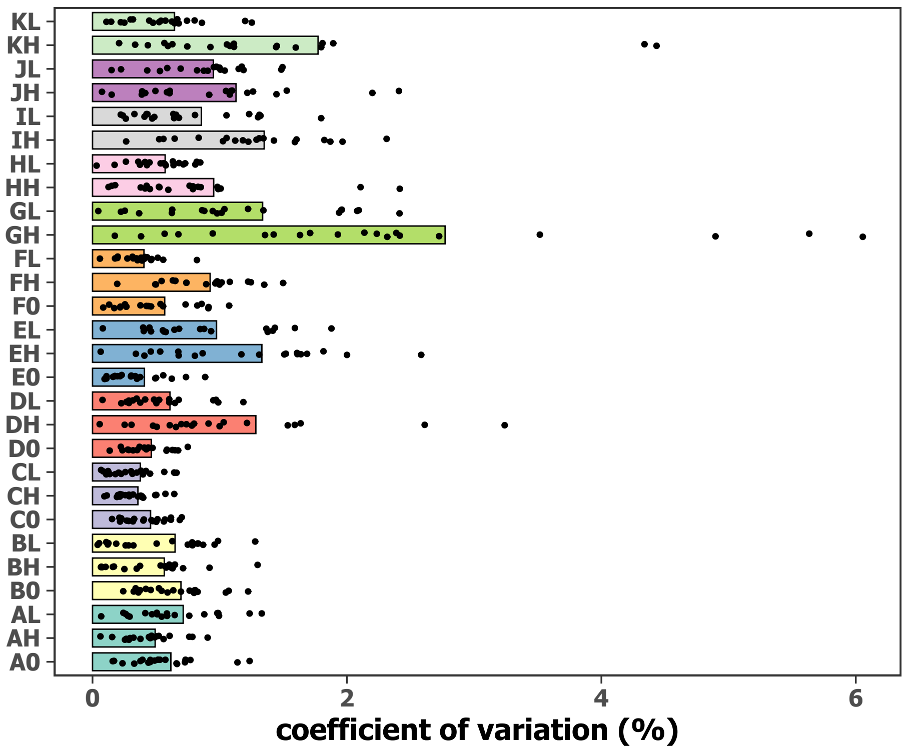

Supplementary Figure S2. Repeatability of protocols for sequencing library construction as evaluated using the DNA mock community. Bar heights represent the quadratic mean of strain-wise coefficients of variation (qmCV, see Methods for details) of measured relative abundances across three technical replicates for each protocol. Black symbols show coefficients of variation for individual strains.

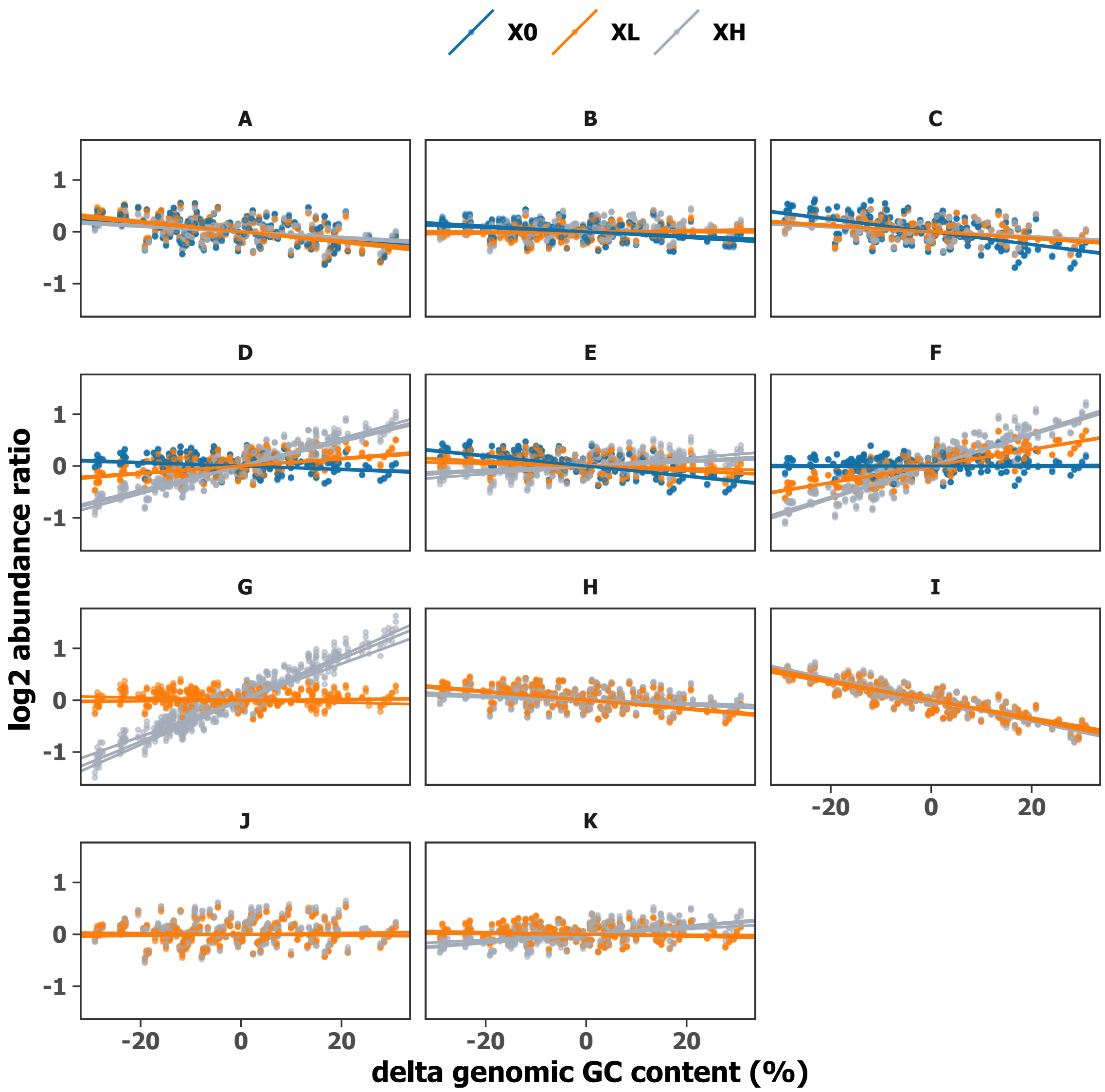

Supplementary Figure S3. Quantification of GC bias. Each data point represents a pair of strains/genomes, with their difference in genomic GC content plotted on the *x*-axis and $\log_2$-fold-difference in relative abundance plotted on the *y*-axis. Solid lines show the intercept-free linear regression fits for each of the three technical replicates per protocol, generated using the function lm in R's stats package. Facets represent different kits and colors reflect varying DNA input amounts and associated conditions for library amplification by PCR (X0, XL and XH). Calculated slopes were used as an overall measure of GC bias and shown in Fig. 1 in the main text.

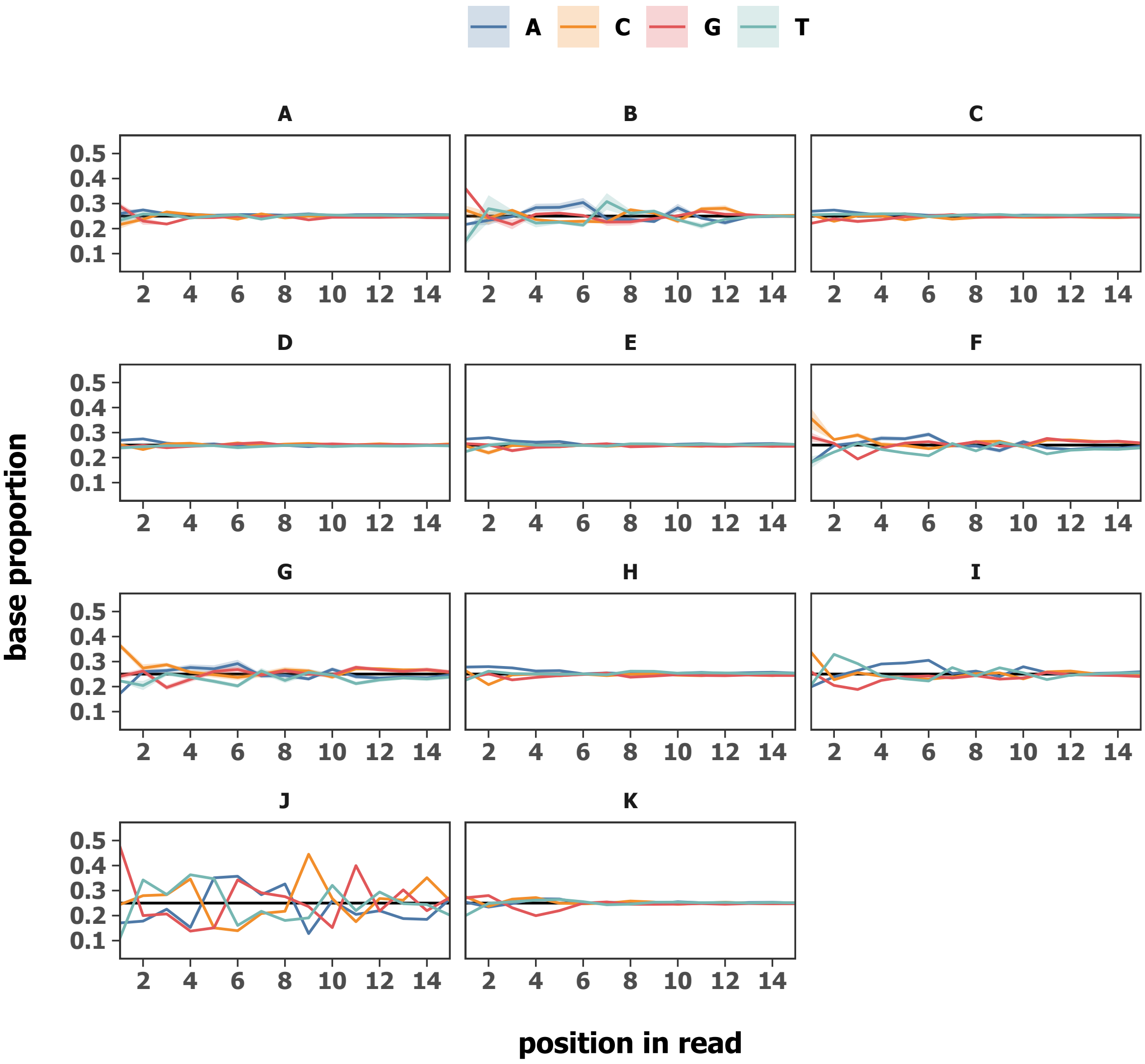

Supplementary Figure S4. Visualization of fragmentation bias introduced during sequencing library construction as evaluated using the DNA mock community. Plots show the base composition for the first 15 cycles of the forward sequencing read, prior to quality trimming. Data are shown as the mean (solid lines) and standard deviation (ribbons, if visible) across different DNA input amounts and three technical replicates for each kit. Per-position base contents were obtained from fastp's json output files. The black horizontal line shows the theoretically expected even base content calculated based on the reference genome sequences.

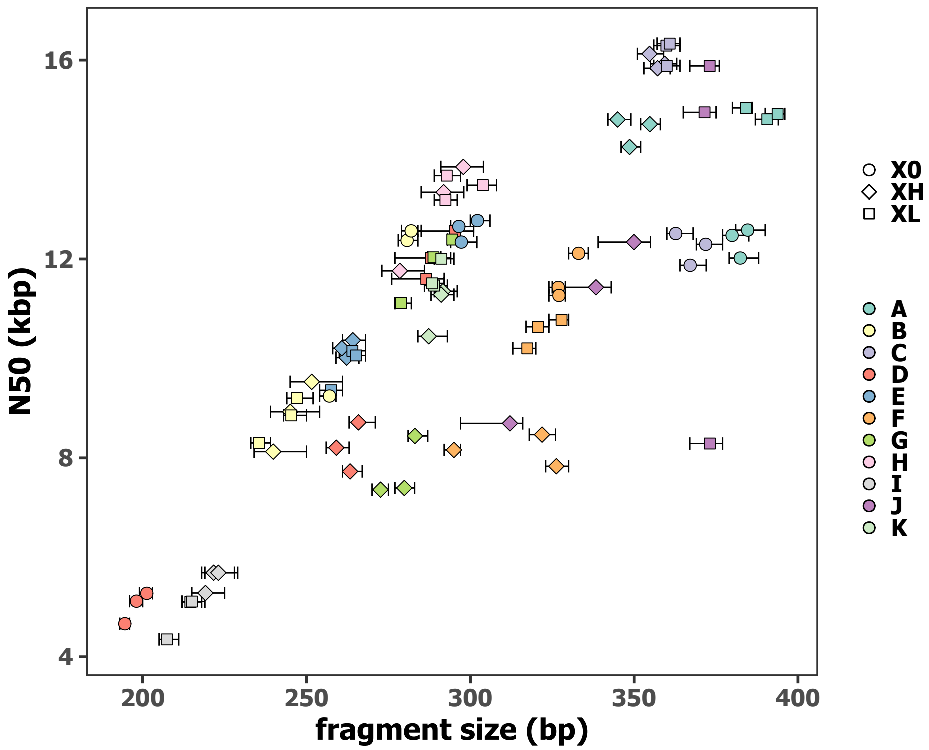

Supplementary Figure S5. Relationship between sequencing library fragment size and N50 values of metagenome assemblies as evaluated using the DNA mock community. For each library, a random set of two million quality-controlled reads pairs were assembled using MEGAHIT. Fragment sizes were estimated by mapping quality-controlled reads against the reference genome sequences using bowtie2 and parsing generated SAM files using samtools and BBMap's reformat.sh script. For the *x*-axis, symbols represent the mean of strain-wise median fragment sizes for individual sequencing library (three per protocol) and error bars represent the range of per-library strain-wise median fragment sizes.

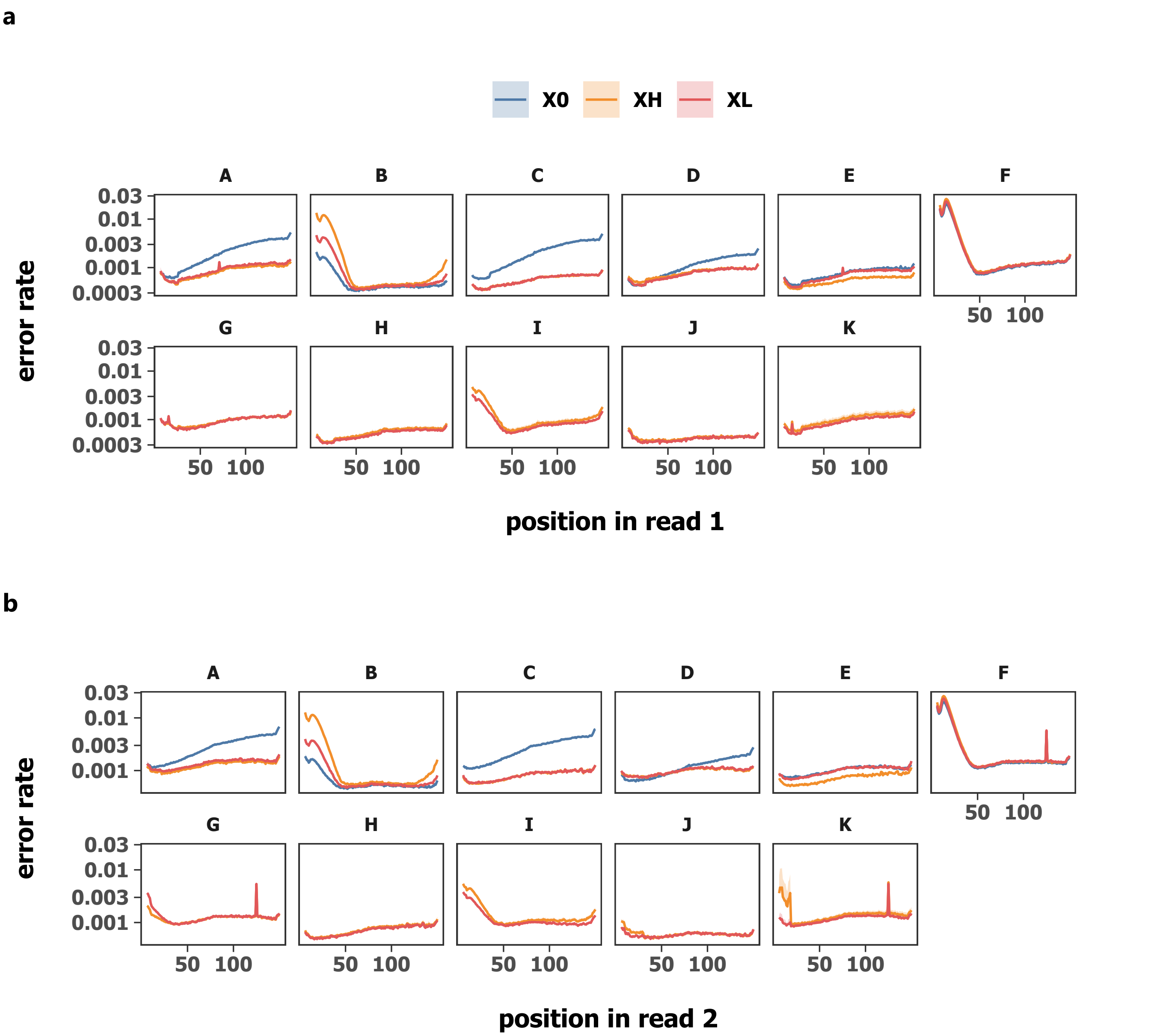

Supplementary Figure S6. Variation in base call error rates across protocols for sequencing library construction as evaluated using the DNA mock community. Error rates were estimated by mapping quality-controlled reads against the reference genome sequences using bowtie2 and parsing generated SAM files using samtools and BBMap's reformat.sh script. A total of two million read pairs per library were analyzed and data represent the mean (solid lines) and standard deviation (ribbons, if visible) of base cell error rates for technical replicates for each protocol. Per-position error rates were calculated as the mean across strains. Panels a and b show data for reads 1 (forward) and 2 (reverse), respectively. Facets represent different kits (A-K) and colors represent different DNA input amounts (X0, XL and XH).

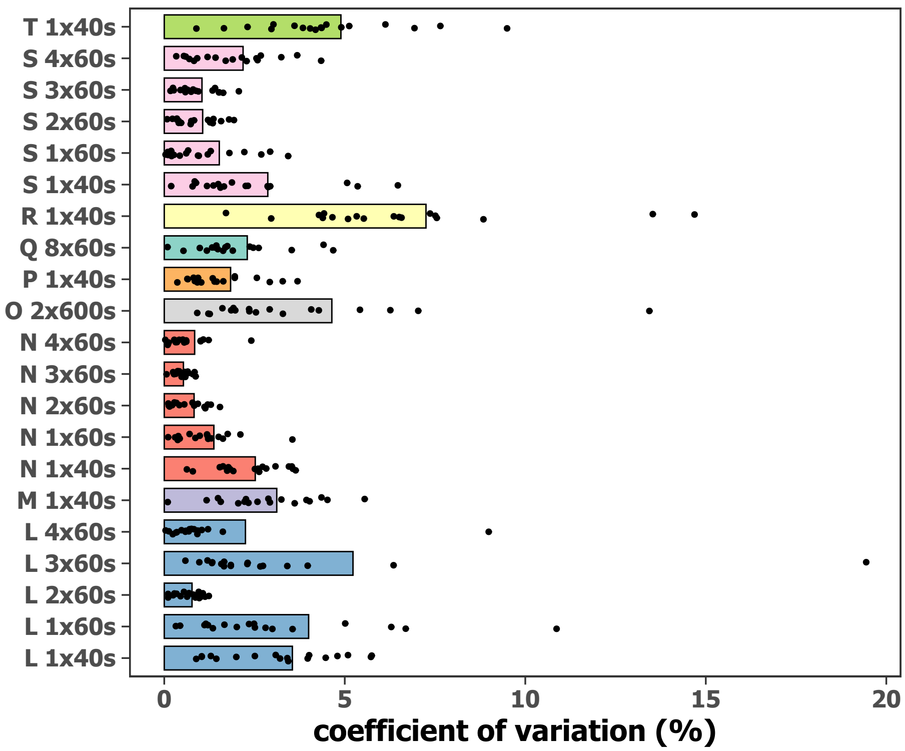

Supplementary Figure S7. Repeatability of protocols for DNA extraction, as evaluated using the cell mock community. Bar heights represent the quadratic mean of strain-wise coefficients of variation (that is, qmCV) of measured relative abundances across two or three technical replicates. Black symbols show coefficients of variation for individual strains. Labels on the *y*-axis consist of kit identifiers and bead-beating regimes (cycles x seconds per cycle), separated by a space.

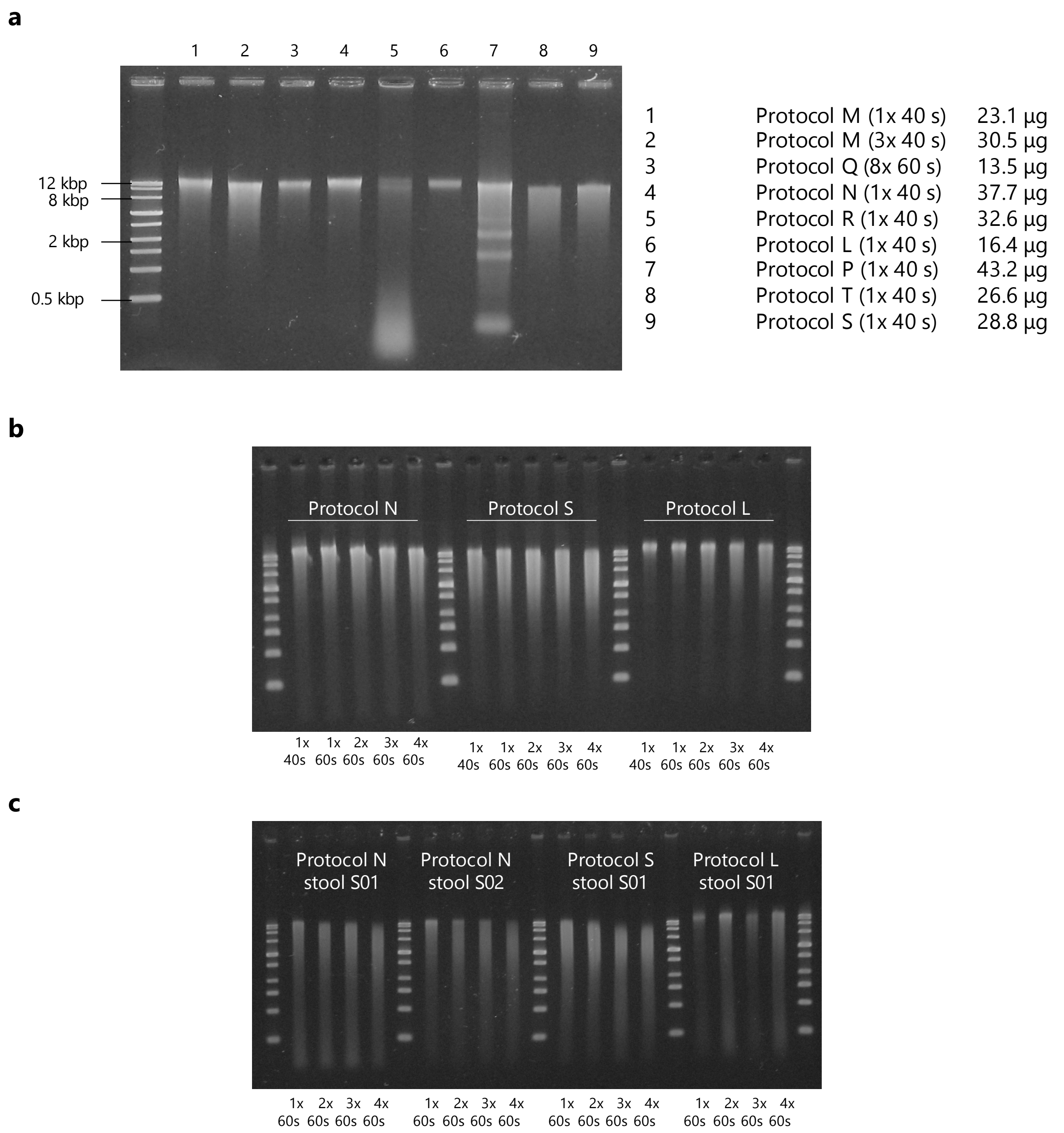

Supplementary Figure S8. (a) Inspection of DNA extracted from the cell mock community using different kits and bead-beating regimes (indicated between brackets in the accompanying table). DNA yields were measured using the Qubit dsDNA Assay Kit and Qubit 3 fluorometer. Note that for protocol P, RNase treatment was not performed, leading to visible rRNA/mRNA bands. (b, c) Evaluation of the effect of bead-beating regime on the size distribution of extracted DNA for the cell mock community (b) and fecal sample(s) (c). In all cases, extracted DNA was run on 1% agarose gels, stained with GelRed nucleic acid stain and a Perfect DNA Markers, 0.5-12 kbp (NovaGen) used as size marker for all gels.

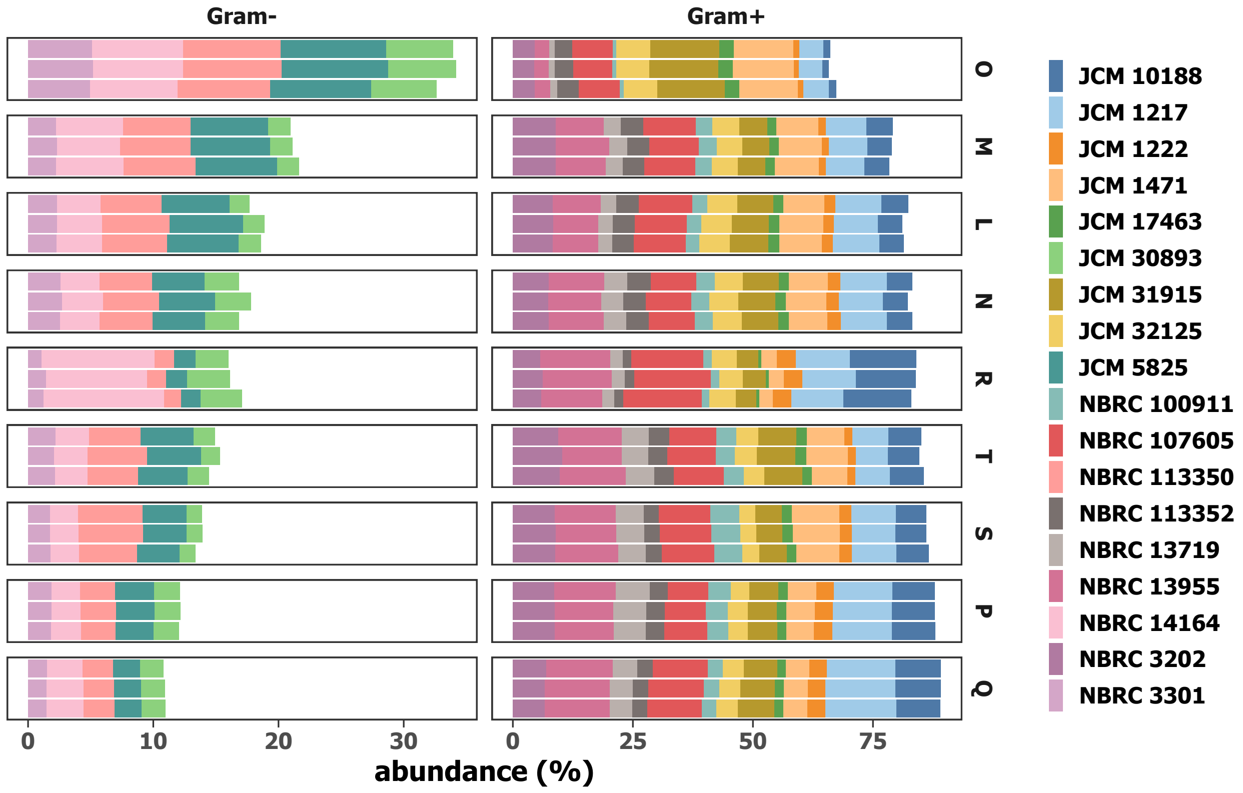

Supplementary Figure S9. Strain-wise relative abundances for the cell mock community processed using different protocols for DNA extraction. Each facet shows the results of three technical replicates. A constant bead-beating regime of 1×40 s was used, except for protocols Q (8×60 s) and protocol O (2×600 s). Facets are sorted row-wise according to the total proportion of Gram-positives and Gram-negatives.

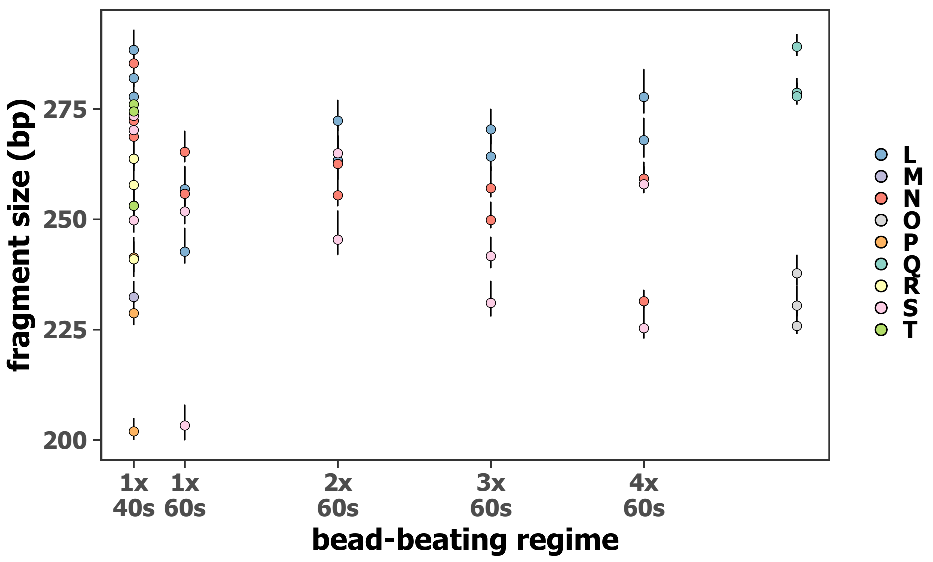

Supplementary Figure S10. Effect of bead-beating regime on library fragment size for the cell mock community. Fragment sizes were estimated by mapping of quality-controlled reads against the reference genome sequences using bowtie2 and parsing generated SAM files using samtools and BBMap's reformat.sh script. For the *y*-axis, symbols represent the mean of strain-wise median fragment sizes for each library and error bars represent the range of strain-wise median fragment sizes for each library.

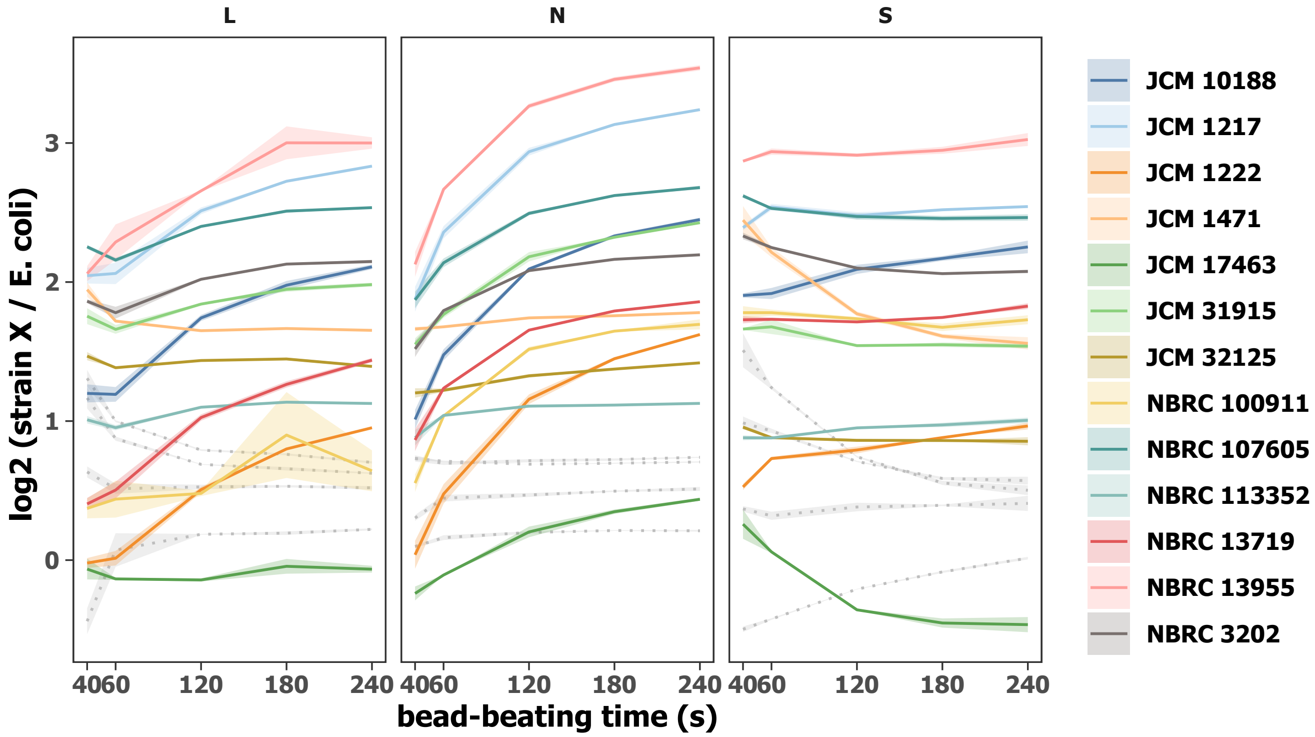

Supplementary Figure S11. Effect of bead-beating regime on the observed abundance of individual strains, relative to *E. coli*. All Gram-negatives are shown as dotted grey lines. Data represent the mean (lines) and standard deviation (ribbons, if visible) of two or three technical replicates. Note that data for protocol N are also shown in Fig. 2 in the main text.

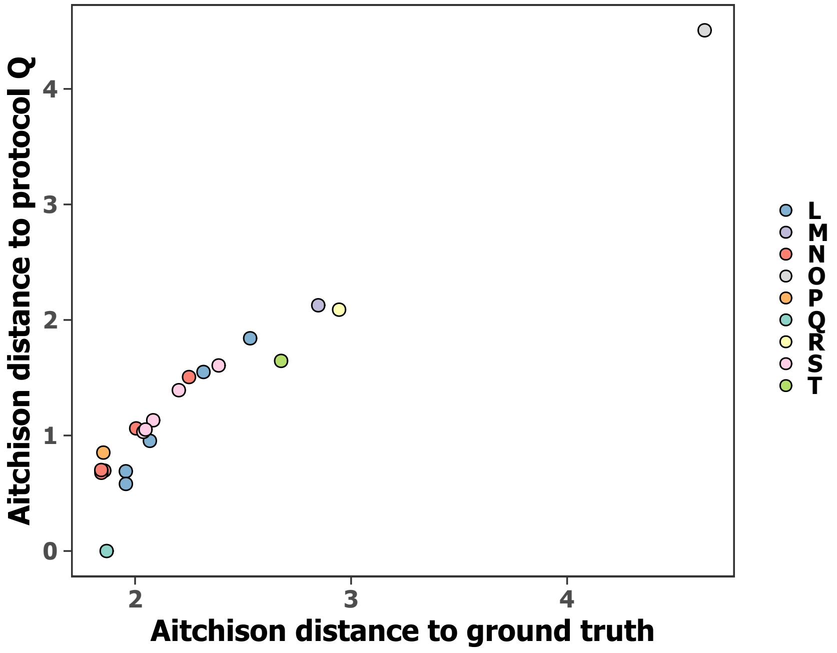

Supplementary Figure S12. Relationship between Aitchison distance to the "ground truth" as determined based on total DNA content (*x*-axis) and to protocol Q (*y*-axis) for ranking of protocols for DNA extractions. The Spearman's rank correlation coefficient is 0.93, showing consistent ranking of protocols. Values for protocol Q were calculated as the closed geometric mean of three technical replicates.

a

b

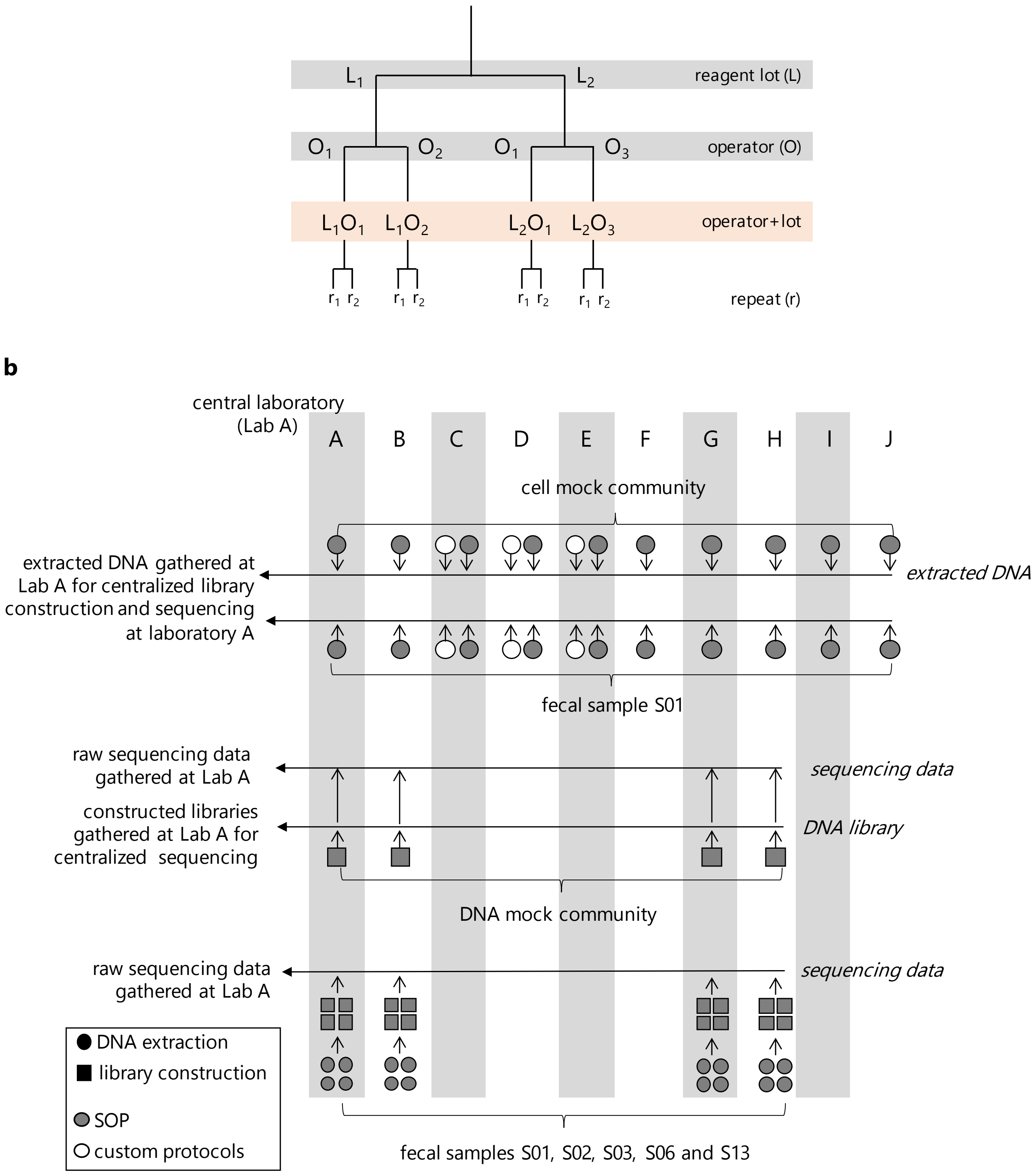

Supplementary Figure S13. (a) Schematic of the experimental design for assessment of intermediate precision associated with varying operators and reagents lots. Based on the experimental design with limited number of operators and reagent lots, we evaluated intermediate precision of operator+lot combinations (*n*=4), each with two measurements performed under repeatability conditions. (b) Schematic of the experimental design for assessment of interlaboratory reproducibility of DNA extraction (circles) and sequencing library construction (squares). All laboratories analyzed each of the samples in duplicate. To assess reproducibility of DNA extraction, DNA was

extracted by ten laboratories (that is, central laboratory Lab A and nine industry-based laboratories, designated B - J) from the cell mock community and fecal sample S01, and DNA gathered at the Lab A for centralized library preparation and sequencing. Three of the above nine industry-based laboratories also performed DNA extraction using a custom protocol. To assess reproducibility of library construction, sequencing libraries were constructed by four laboratories (that is, the central laboratory Lab A and three industry-based laboratories, designated B, G and H) from the DNA mock community, and DNA gathered at the Lab A for centralized sequencing. Laboratories B, G and H also performed sequencing at their own facilities and shared raw sequencing data with Lab A. Finally, Labs A, B, G and H also extracted DNA, prepared libraries, and generated sequencing data for fecal samples S01, S02, S03, S06 and S13; these data were analyzed at Lab A.

a

b

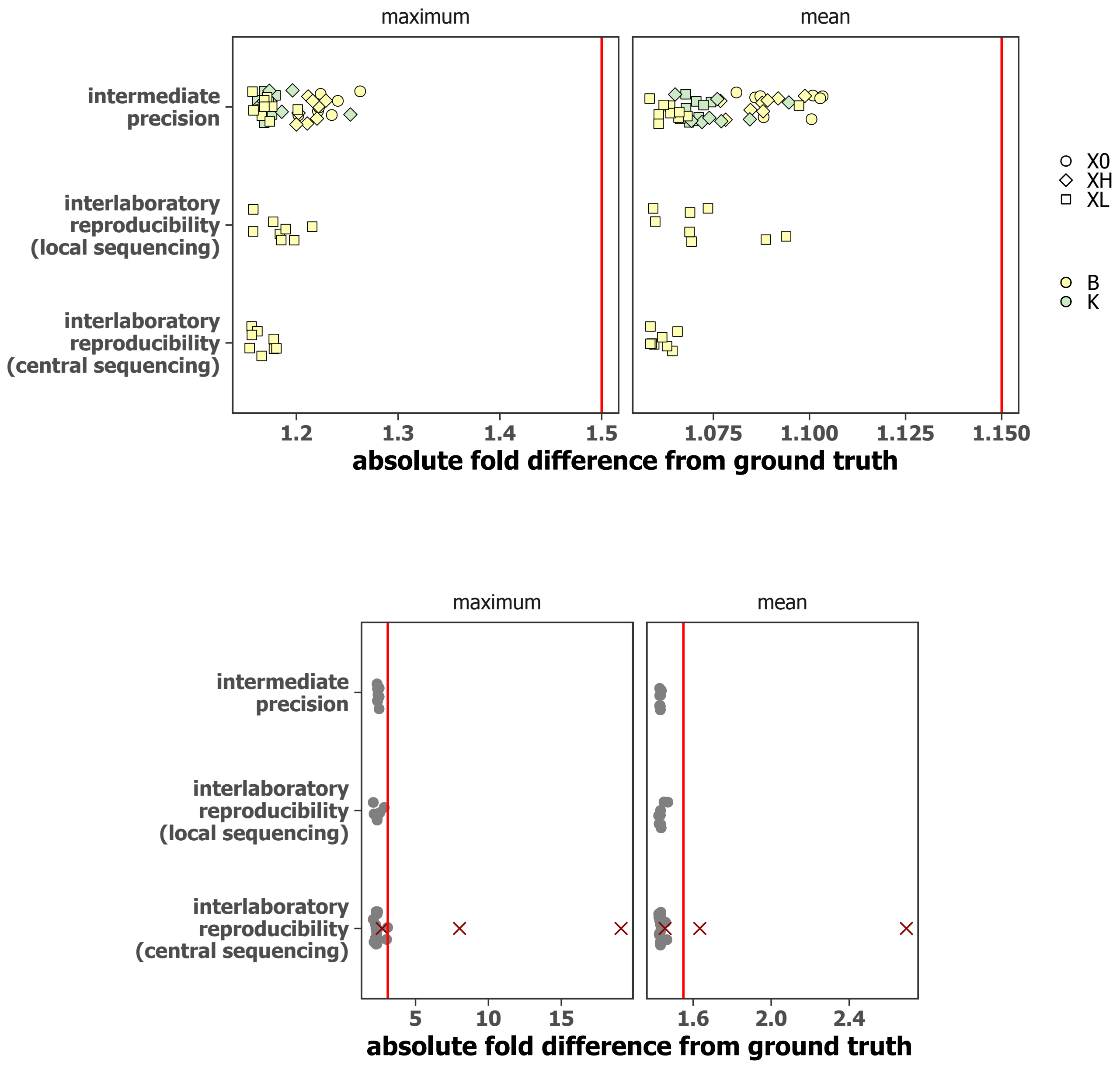

Supplementary Figure S14. Evaluation of accuracy (agreement of individual measurement results to the ground truth) generated as part of the intermediate precision and interlaboratory reproducibility studies. The vertical red lines show the thresholds for accuracy, in terms of geometric mean of strain-wise absolute fold errors and maximum of strain-wise fold errors to the ground truth (see Table S3). Panels a and b show data for the DNA and cell mock community, respectively. Custom protocols for DNA extraction in panel b are shown as red x symbols.

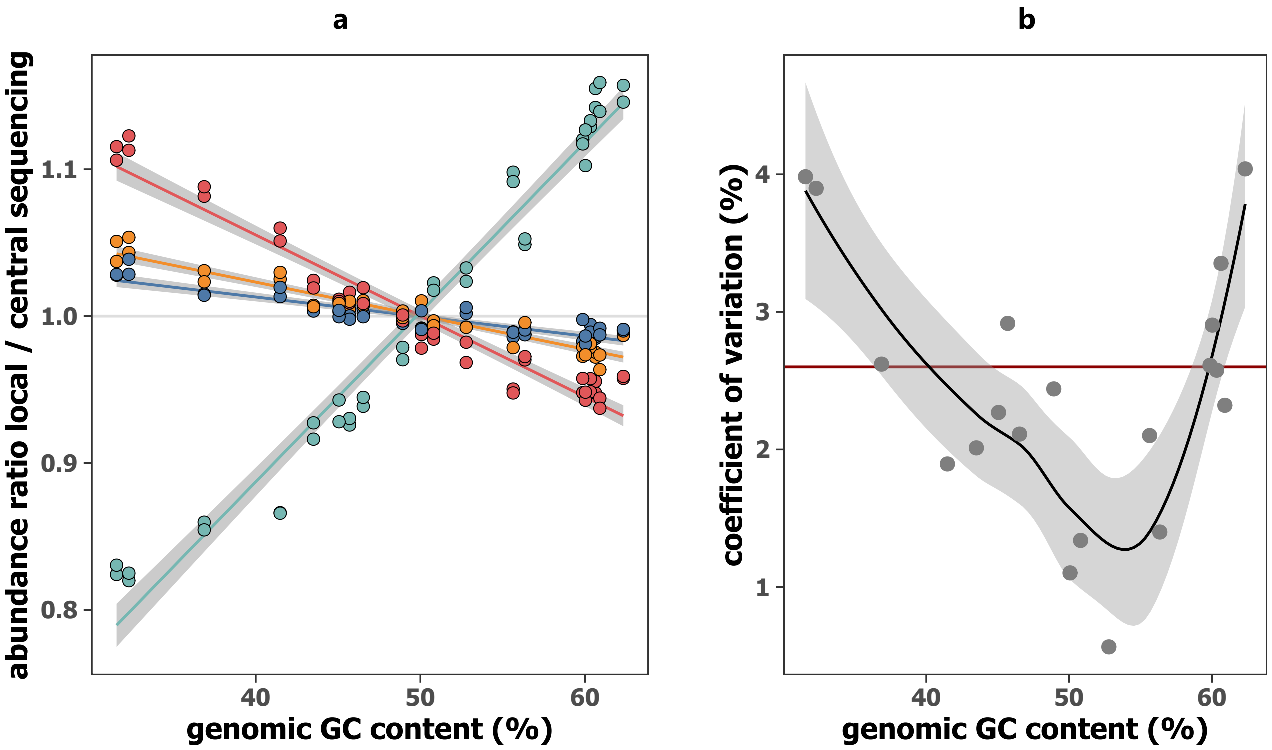

Supplementary Figure S15. (a) Illustration of GC-dependent bias associated with sequencing runs/platforms as observed in the interlaboratory study. The *y*-axis represents the ratio of the observed strain-wise abundances in sequencing data generated by the central laboratory and external laboratories (local). Data are for the DNA mock community, with sequencing libraries constructed using protocol BL. Colors represent four participating laboratories (that is, the central laboratory Lab A and three industry-based laboratories). Note that the laboratory with the highest bias used a NovaSeq platform (greenish color) while all other laboratories used a NextSeq 500/550 instrument. Data from the central laboratory Lab A shown in bluish color represent technical sequencing replicates. Symbols represent data from individual replicates (*n*=2). The linear regression lines and confidence intervals were generated using ggplot2's geom_smooth function. (b) Evaluation of repeatability of sequencing by repeated sequencing (*n*=4) of a single DNA mock community library (generated using protocol C0) in four different NextSeq 500 sequencing runs and its relationship to GC content. The red horizontal line represents the quadratic mean of strain-wise coefficients of variation (that is, qmCV). The smoothed trend line (generated using ggplot2's geom_smooth function) was added to visualize the U-shaped relationship between GC-content and between-sequencing-run variability in strain-wise abundances.

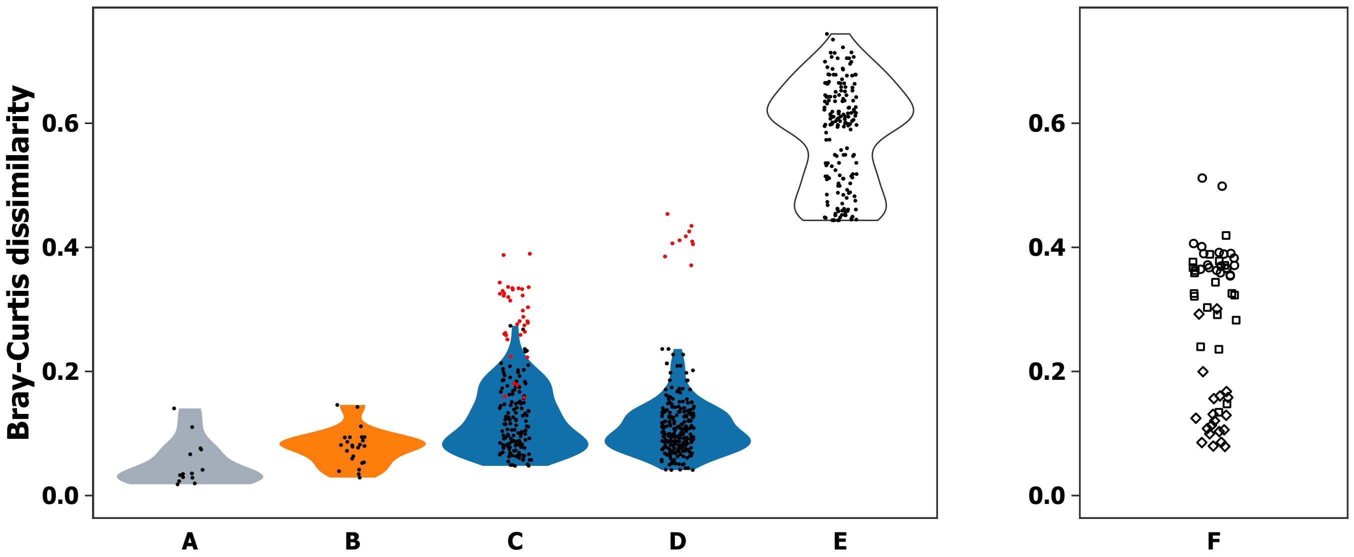

Supplementary Figure S16. Same as Fig. 3d in the main text, based on Bray-Curtis dissimilarities. Pairwise distances are calculated according to the categories on the *x*-axis: A: dissimilarities between replicated DNA extractions for the intermediate precision and interlaboratory reproducibility assessments; B: dissimilarities between (operator+lot)-different DNA extractions for the intermediate precision study; C: dissimilarities between (laboratory)-different DNA extractions for the interlaboratory reproducibility study; D: dissimilarities between (laboratory)-different data generation for the interlaboratory reproducibility study for samples S01-S16, with all steps from DNA extraction to sequencing performed by the participating laboratories; E: within-laboratory dissimilarities between different fecal samples; F: dissimilarities between custom DNA extraction protocols and protocol N for sample S01. Data from laboratories for which at least one the replicated measurements was considered as an outlier are shown in red (see Fig. S17).

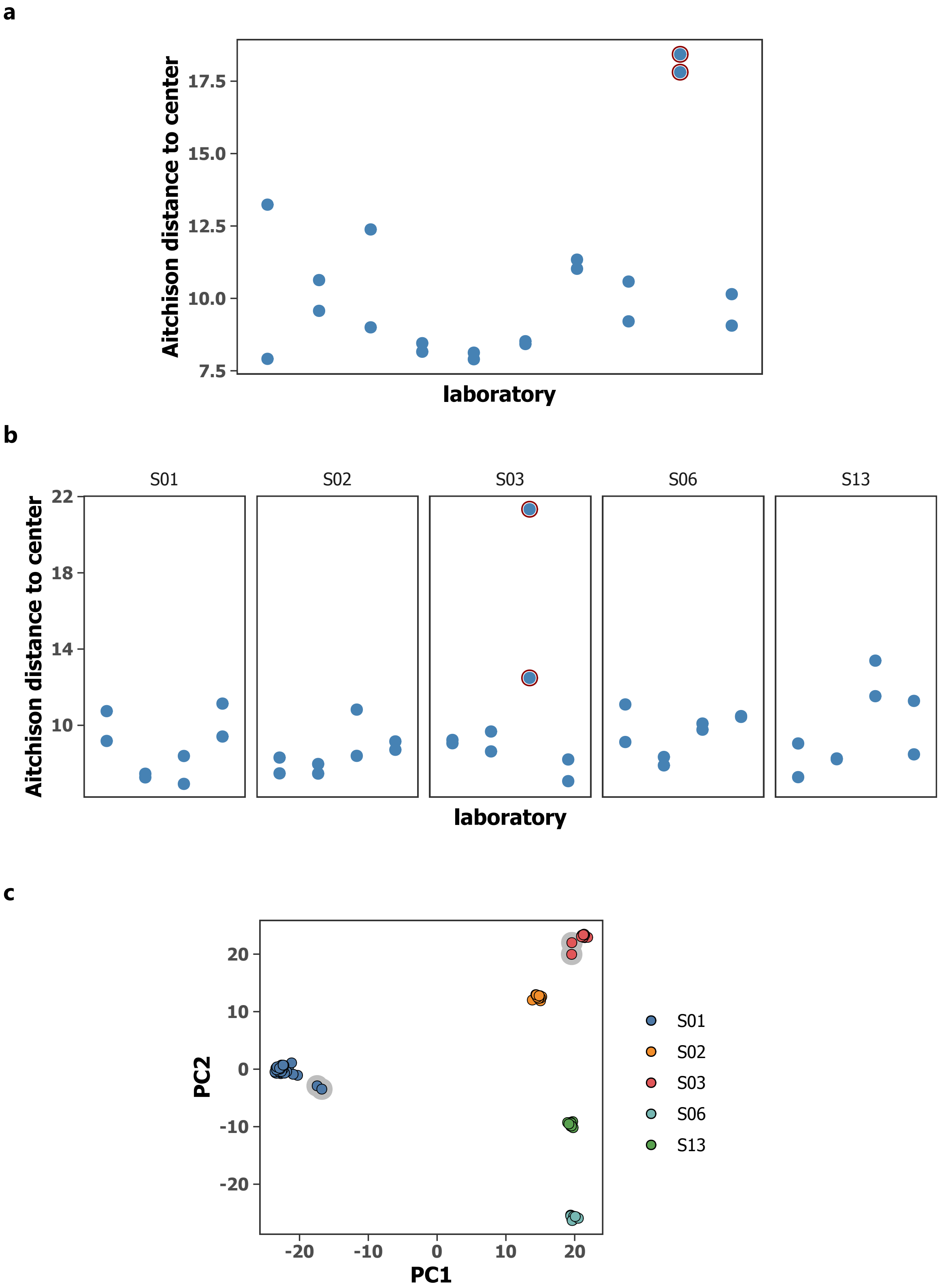

Supplementary Figure S17. Evaluation of outliers for assessment of interlaboratory reproducibility of DNA extraction from fecal sample S01 (panel a, *n*=10 laboratories) and reproducibility of the full metagenomic analysis pipeline (that is, including DNA extraction, sequencing library construction and sequencing) for five fecal samples (panel b, *n*=4 laboratories). Datapoints represent Aitchison distances to the geometric centre of

each dataset. Laboratories yielding outlying measurement results are marked by a red circle. Panel c shows a compositional PCA ordination plot (that is, based on Euclidean distances after clr transformation of relative abundances) with measurement results from outlying laboratories highlighted with a filled grey circle.

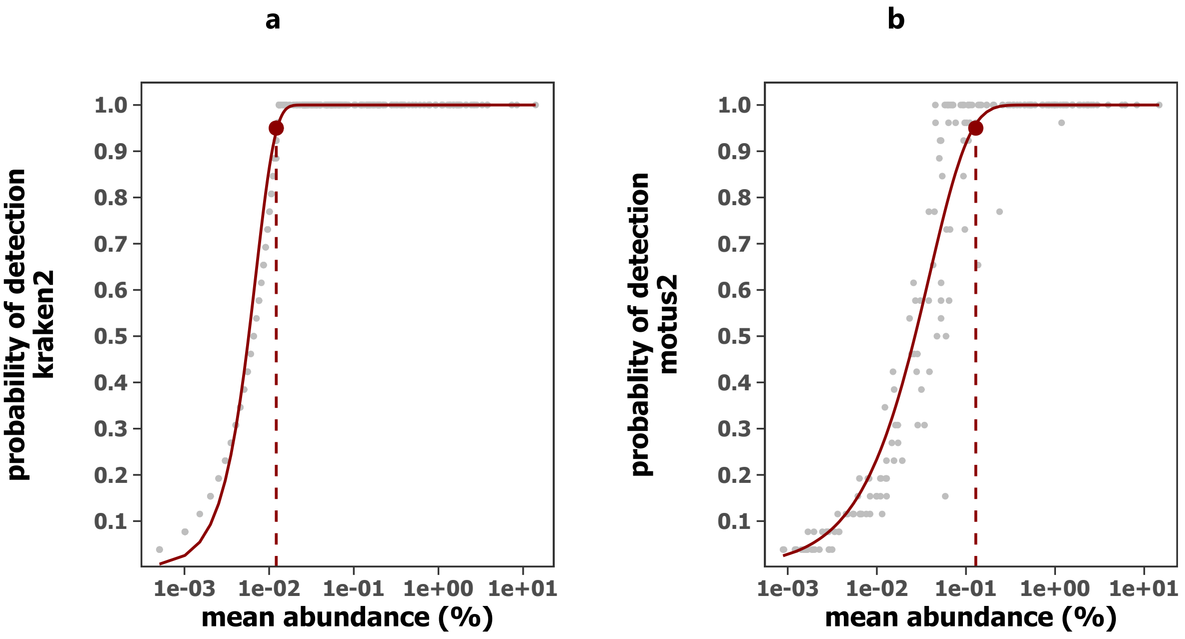

Supplementary Figure S18. Determination of the limit-of-detection (LOD) through regressing the probability of detection (POD) of each species/mOTU (*y*-axis) to its mean abundance (*x*-axis) across available measurement results for sample S01. A total of 26 datasets were included in the analysis, namely 8 data sets generated as part of the assessment of intermediate precision of DNA extraction, and 18 data sets generated as part of the assessment of interlaboratory reproducibility of DNA extraction after exclusion of outlying data (see Fig. S17). Panels a and b show data for kraken2 (species-level) and mOTUs2 (OTU level), respectively. The model was generated by regressing the POD to the mean abundance (log-transformed), using a generalized linear model with family binomial and complementary log-log (cloglog) link function using R stats' glm function. The vertical dashed red lines represent the estimated LODs, with a fitted POD of 95%.

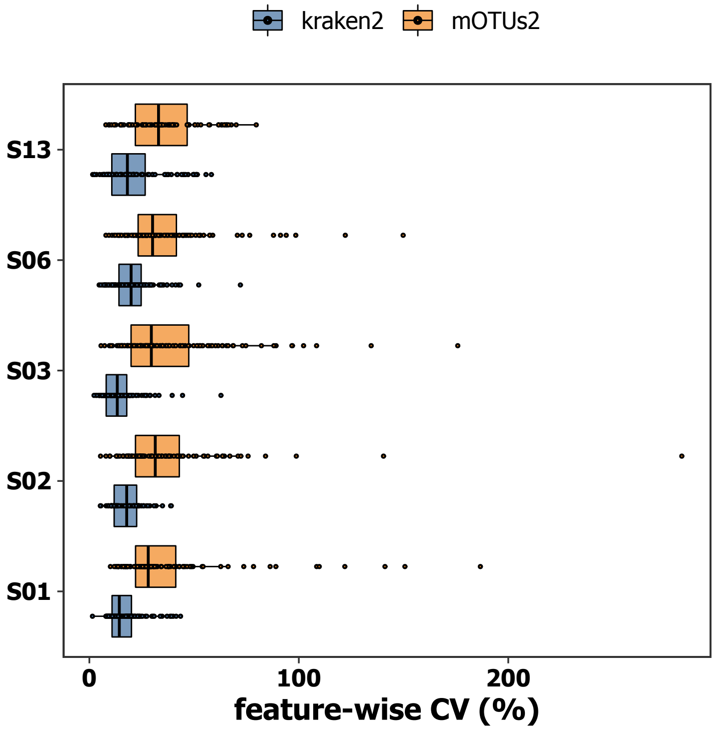

Supplementary Figure S19. Interlaboratory variation in species/mOTU abundances (feature-wise coefficient of variation, CV, %) across the entire measurement workflow, from DNA extraction to sequencing, based on data from four laboratories. Only features with a mean abundance of >0.05% were considered. Boxplots show the distribution of feature -wise coefficients of variation in observed abundances and were generated using ggplot2's geom_boxplot function. Fill colors represent taxonomic profilers.

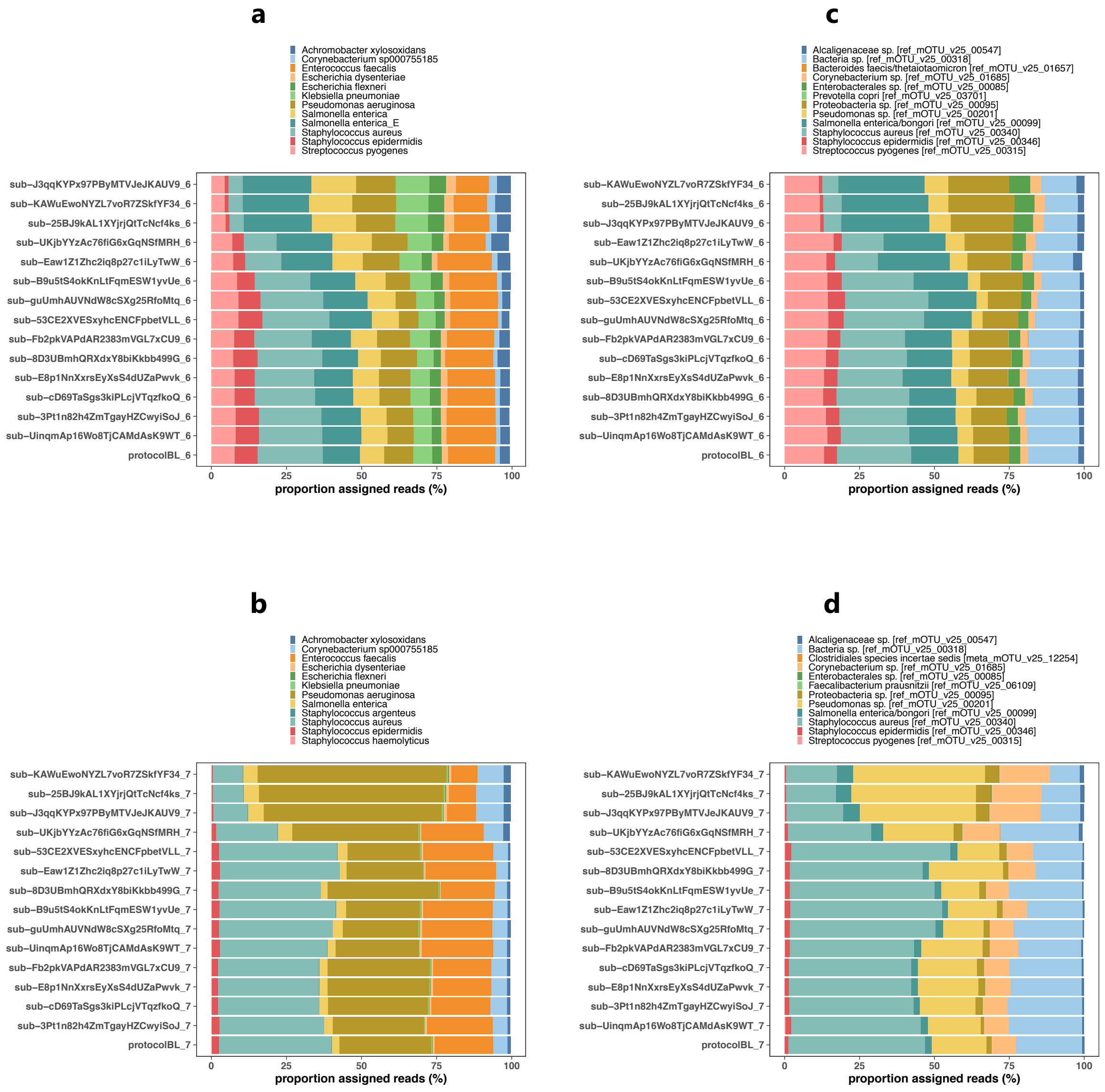

Supplementary Figure S20. Results of the MOSAIC Standards Challenge samples for the DNA mock communities (sample 6, NIST Mix A, in panel a and c; sample 7, NIST Mix B, in panel b and d). Panels a and c and panels b and d show results based on taxonomic profiling using kraken2 (species-level) and mOTUs2 (mOTU-level), respectively. Data generated using our recommended protocol BL are shown at the bottom of each chart. Samples are ordered along the *y*-axis based on their Bray-Curtis dissimilarity to protocol BL, independently in panels a and b. Note that only the top-12 most abundant species/mOTUs (based on feature-wise maximum abundance across all samples) are plotted.

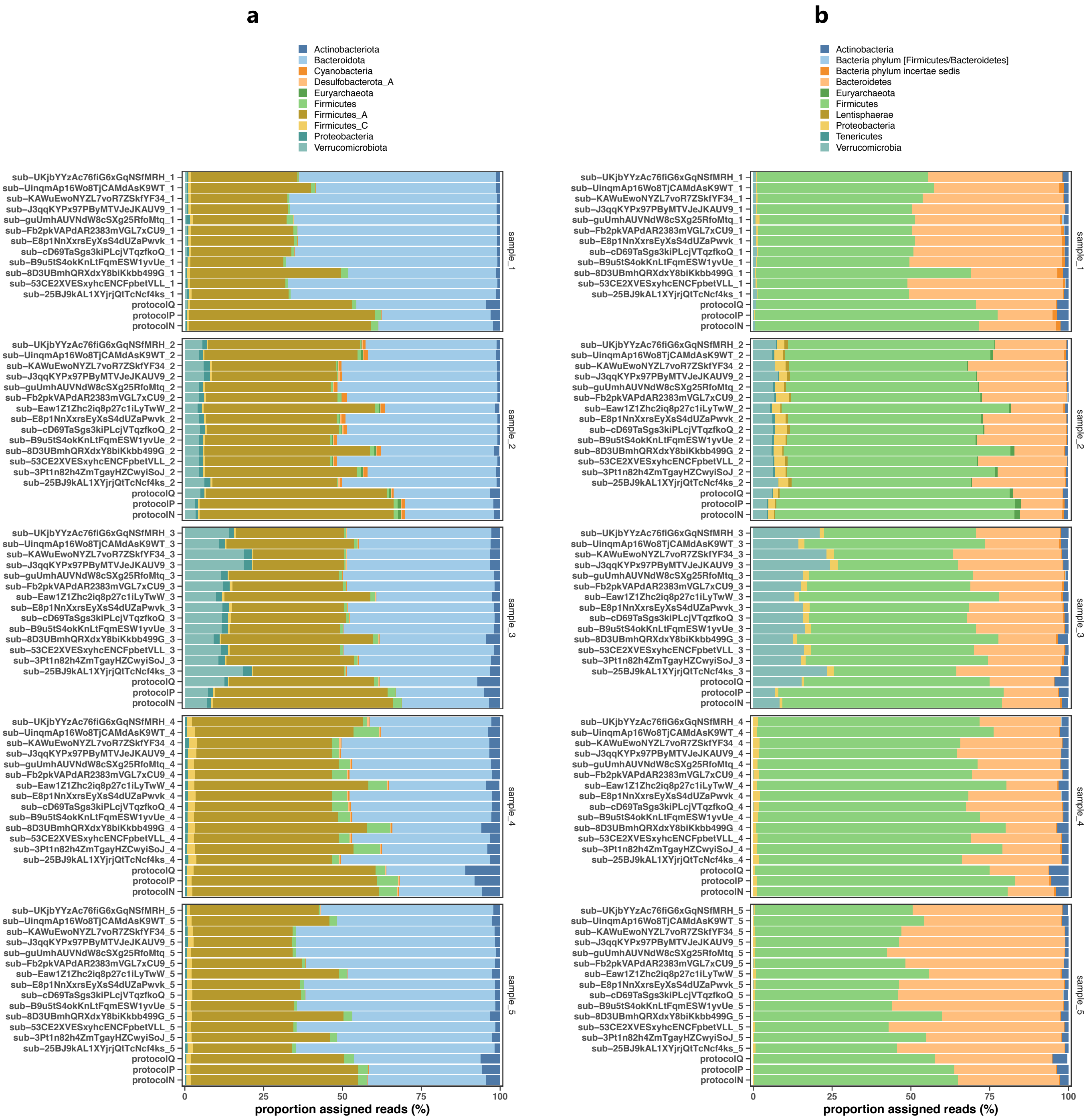

Supplementary Figure S21. Results of the MOSAIC Standards Challenge samples for fecal samples 1 – 5. Bar charts show the proportion of assigned reads at the phylum level using kraken2 (a) or mOTUs2 (b). Data for protocols P, N and Q were generated in this study.

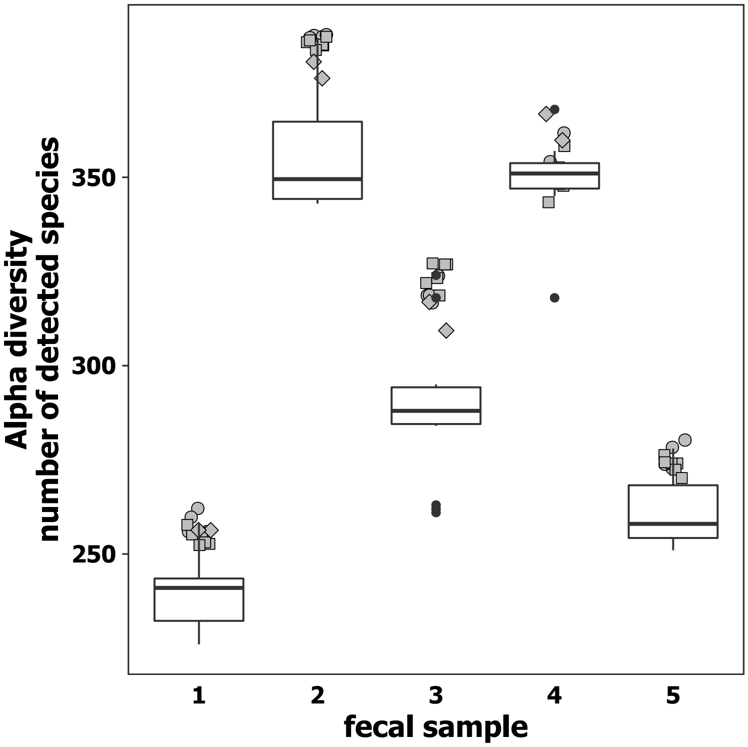

Supplementary Figure S22. Alpha diversity of the MOSAIC Standards Challenge samples for fecal samples 1 – 5: comparison of species richness (number of species with non-zero assigned reads) among protocols. Boxplots represent the distribution of species richness observed for public data sets and colored symbols show data for protocols P (circles), N (squares) and Q (diamonds) as generated in this work. Boxplots were generated using ggplot2's geom_box function.

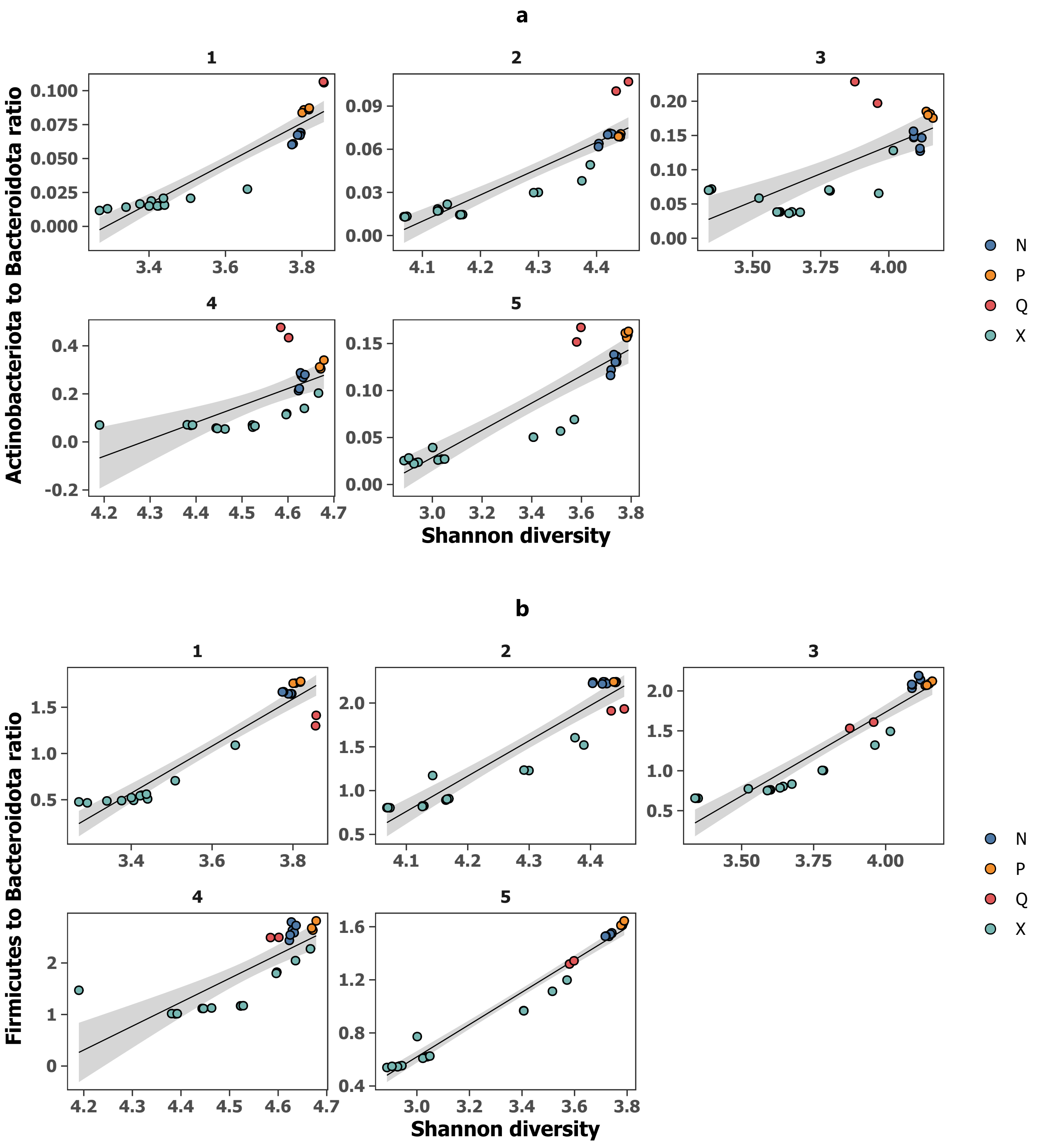

Supplementary Figure S23. Relationship between relative recovery of typical Gram-positives and Gram-negatives and Shannon diversity, based on data from the MOSAIC Standards Challenge. Shannon diversity was estimated based on species-level taxonomic profiles generated using kraken2. Protocol X represents publicly available data. The linear regression line and confidence intervals were generated using ggplot2's geom_smooth function. Subpanels labeled 1 through 5 represent the different fecal samples included in the MOSAIC Standards Challenge.

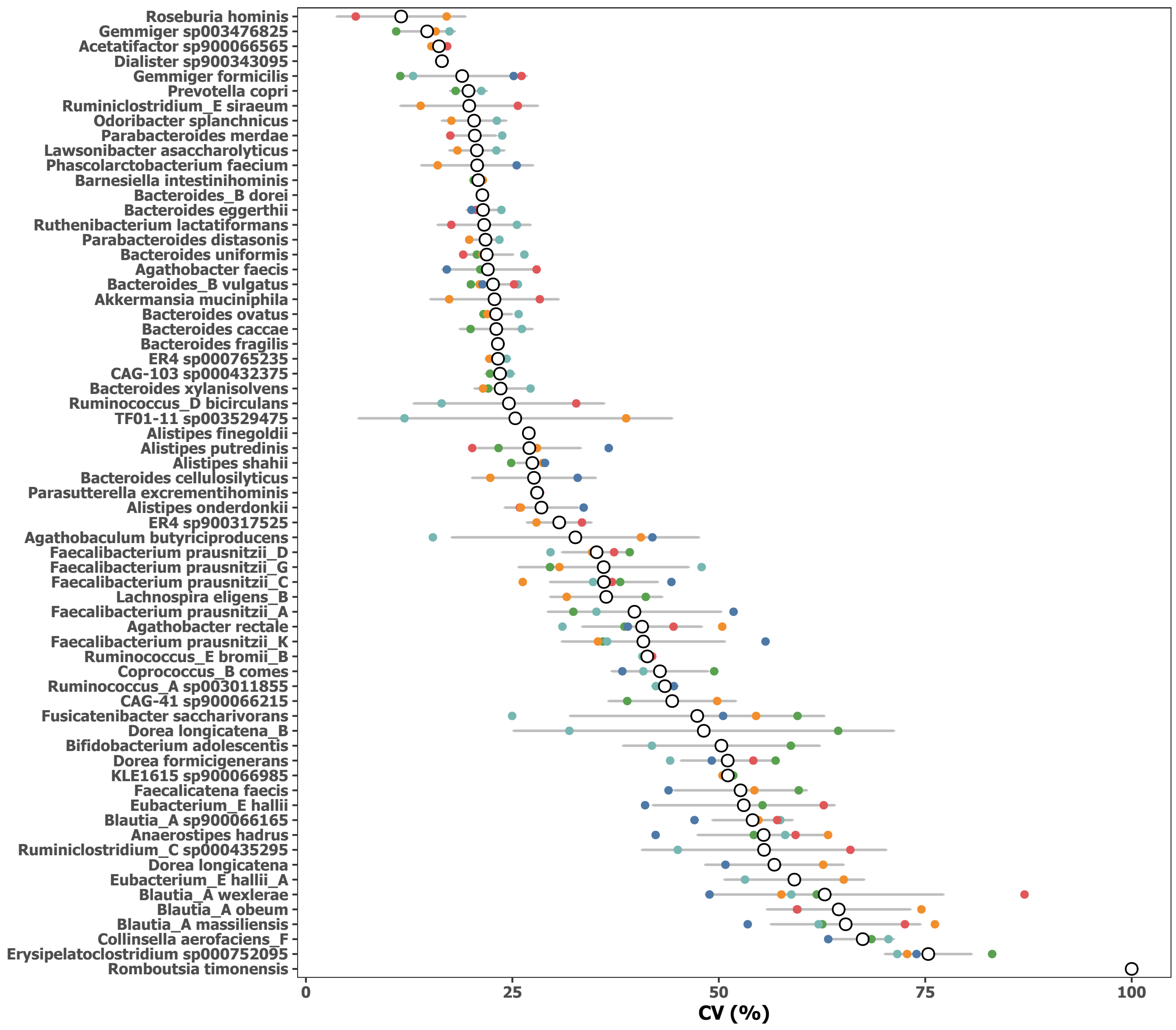

Supplementary Figure S24. Species-wise coefficient of variation (CV) for the MOSAIC Standards Challenge data. Colored symbols represent data for individual fecal samples (*n*=5), white circles and lines represent the mean and standard deviation across fecal samples. Symbol colors represent the five different fecal samples included in the MOSAIC Standards Challenge. Only species with a mean abundance of >0.5% in at least two samples were considered in the analysis. Species abundances were estimated using kraken2.

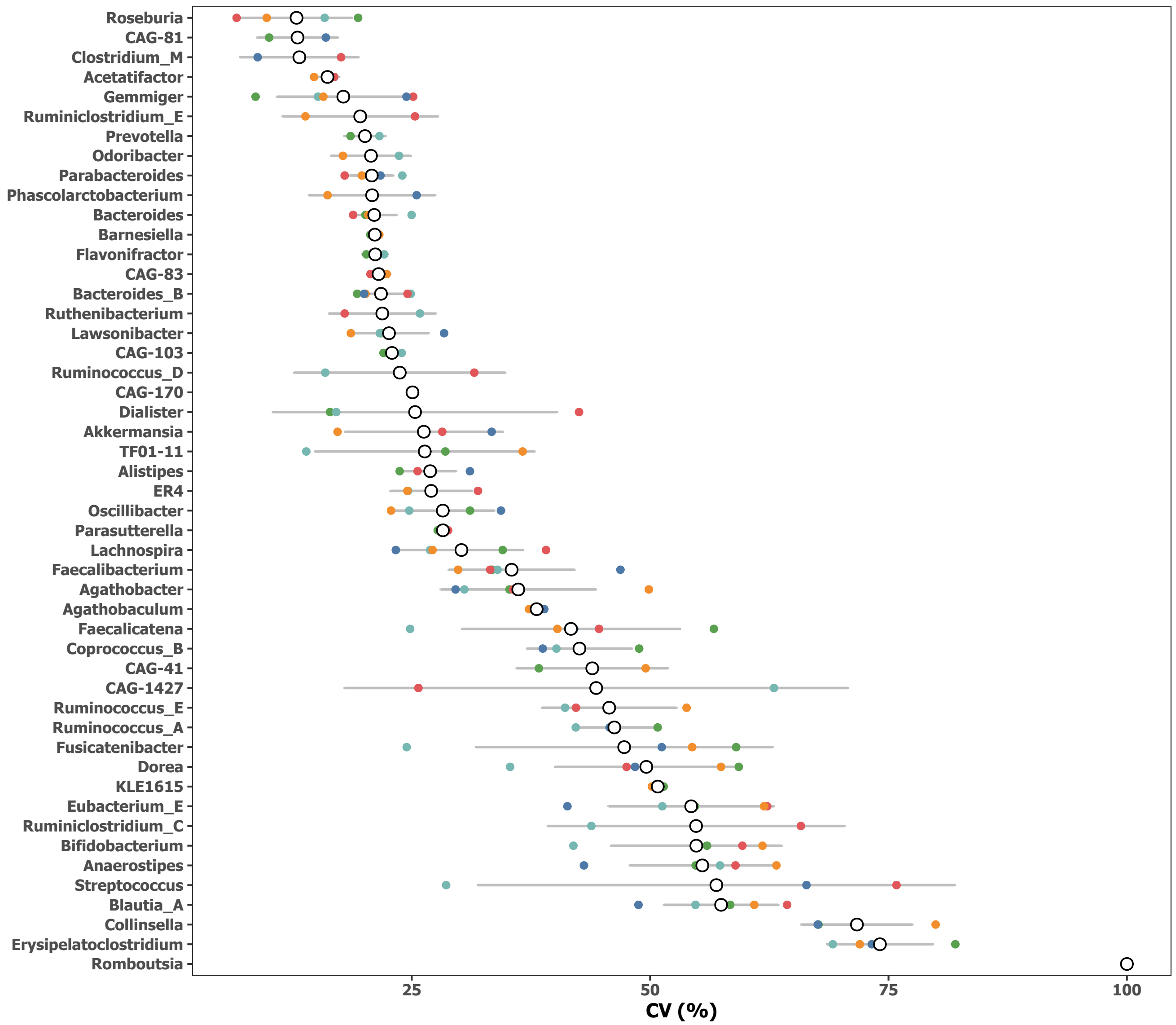

Supplementary Figure S24, cont'd. Genus-wise coefficient of variation (CV) for the MOSAIC Standards Challenge data.

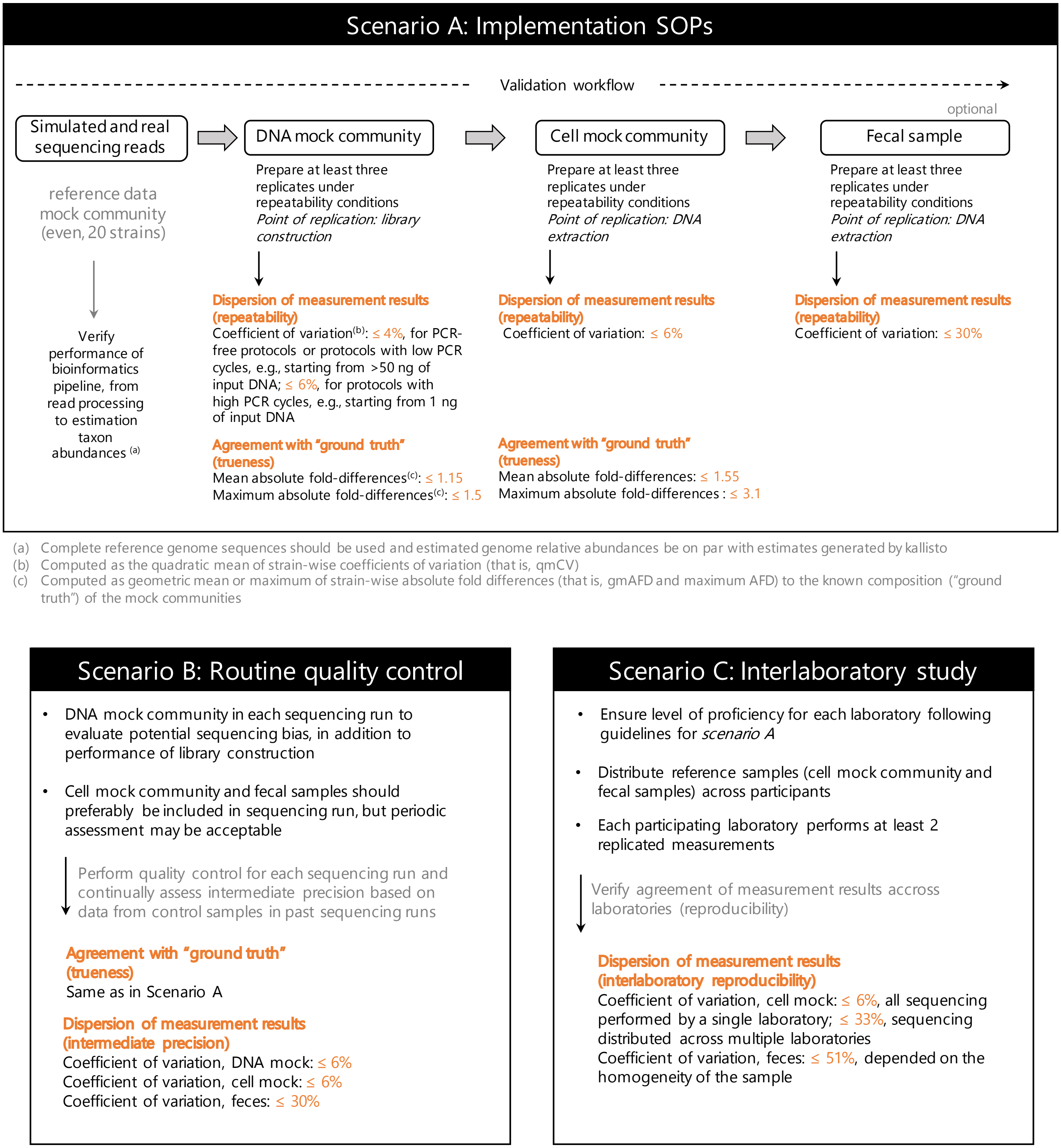

Supplementary Figure S25. Illustration of guidelines and best practices in three case scenarios. Note that proposed values may not fully apply to other mock communities and/or fecal samples with different composition due to sample-specific effects, different homogeneity, different degrees of "uncertainty" in reference value assignments ("ground truth"), *etc*.

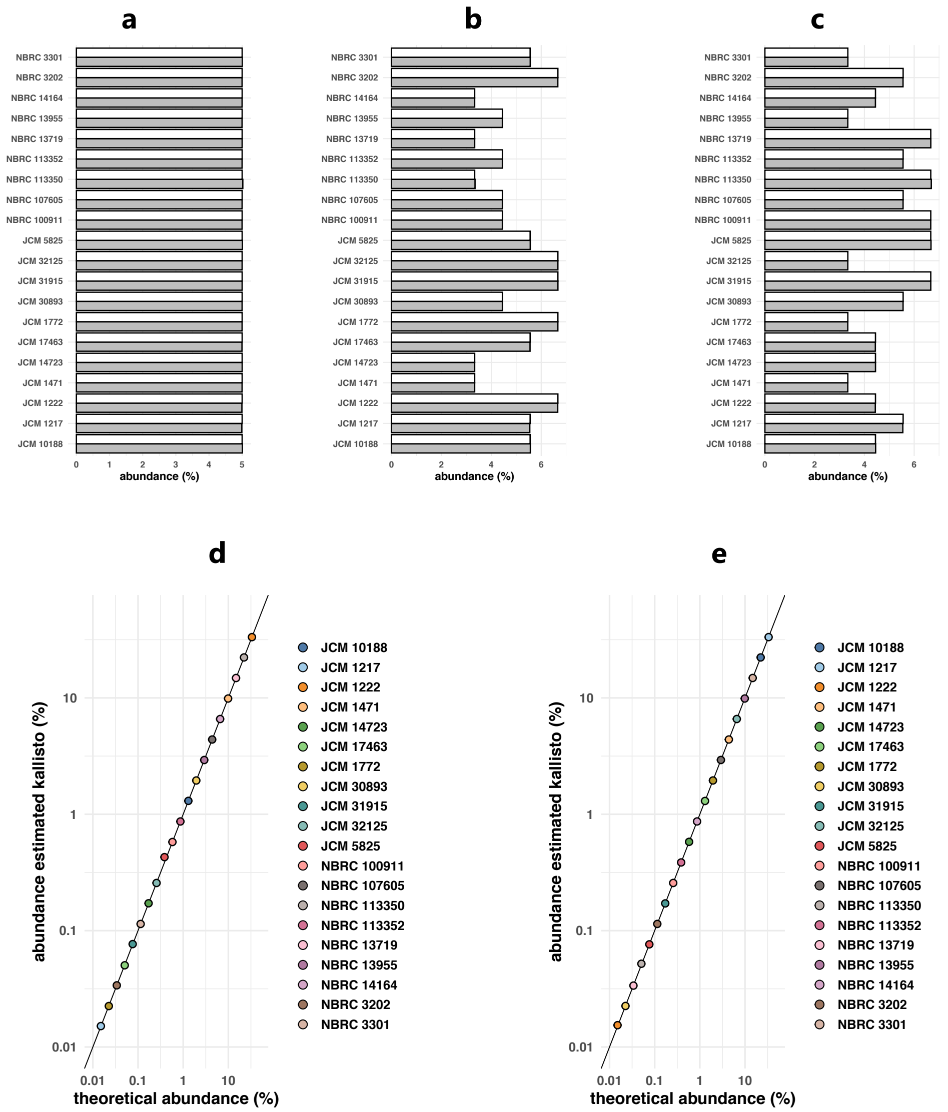

Supplementary Figure S26. Performance of kallisto for alignment-free quantification of genome relative abundances (GRAs), as evaluated using simulated sequencing data. For the upper three plots (a – c), the theoretical and estimated abundances are shown in grey and white, respectively. Panel a shows the results for an even composition and panels b and c show results for moderately staggered compositions, with ranges in strain-wise abundances comparable to the observed ranges for the DNA mock community measured with different protocols for sequencing library construction. The bottom panels (d – e) show the results for highly staggered compositions to highlight the accuracy of quantification of the two subspecies of *Bifidobacterium longum*, namely *B. longum* subsp. *infantis* strain JCM 1222 and *B. longum* subsp. *longum* strain JCM 1217. Simulated sequencing reads (1 million read pairs per simulated read data set for a given mock community) were generated using BBMap's randomreads.sh command, with options paired=t gaussian=t minlength=151 maxlength=151 mininsert=0 maxinsert=400 maxq=36 midq=28 minq=20 adderrors=t. For read pairs with short insert sizes, adapter sequences were added by specifying the options fragadapter=AGATCGGAAGAGCACACGTCTGAACTCCAGTCACTTATAACCATCTCGTATGCCGTCTTCTGCTTGAAAAGGGG and fragadapter2=AGATCGGAAGAGCGTCGTGTAGGGAAAGAGTGTTCGATATCGTGGGG. Note that simulated read data were quality trimmed using fastp to ensure consistency with real sequencing data.

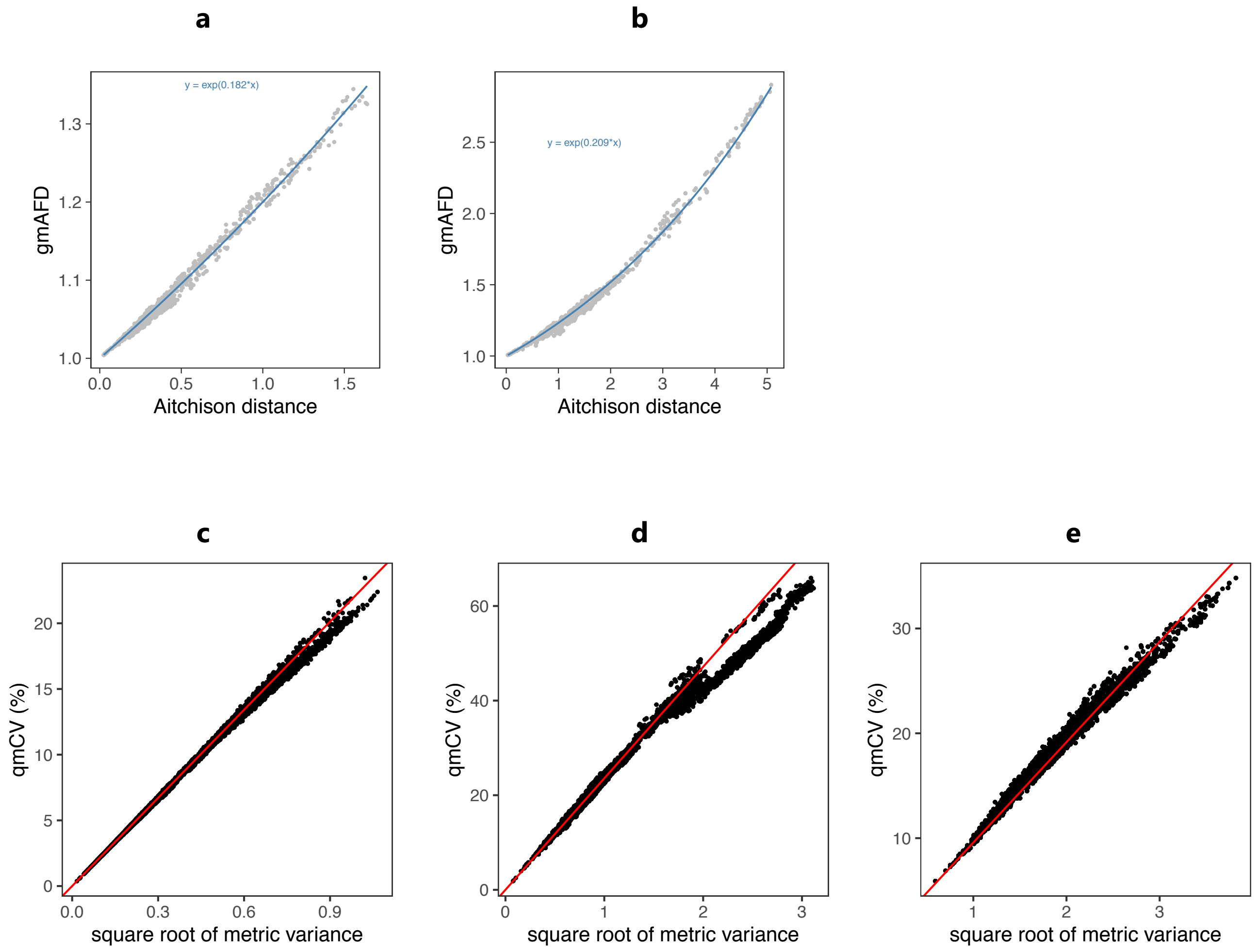

Supplementary Figure S27. (a,b) Relationship between the Aitchison distance and geometric mean of strain-wise absolute fold differences (gmAFD) for (a) the DNA mock community and (b) the cell mock community. Data points were generated based on 1,000 random pairs of measurements, using data from phase I (comparison of methods) of this study. The blue line represents the fitted exponential model, generating using the function nls in R’s stats package. (c-e) Relationship between the square root of the metric variance (*x*-axis) and quadratic mean of strain-wise coefficients of variation (qmCV) for (a) the DNA mock community, (b) the cell mock community and (c) fecal sample S01. The red line represents the proportionality constant of $D^{-1/2}$, where D represents the number of strains/species considered in the analysis. Data points were generated based on 10,000 random subsets of three measurements each, using data from phase I (comparison of methods) for the DNA and cell mock communities and data from phase II (evaluation of intermediate precision and interlaboratory reproducibility) for the fecal sample.

Supplementary Tables

Supplementary Table S1. Overview of the DNA and cell mock communities developed in this study.

species	strain	GenBank/Nucleotide accession number	GenBank assembly accession	genome size (bp)	genome GC content (%)	cell wall type (Gram type)	abundance in DNA mock community (%)	abundance in cell mock community (%)	reference genome assembly	Cultivation medium, atmosphere, temperature, time
Collinsella aerofaciens	JCM 10188[T]	CP048433<br>CP048434<br>CP048435	GCA_010509075.1	2,455,328	60.6	positive	5.0	5.3	Tourlousse *et al.* (2020a)	modified GAM broth + 1% glucose, anaerobic (N_2 atmosphere), 37 °C, 24 h
Blautia producta	JCM 1471[T]	CP048626	GCA_010669205.1	6,197,116	45.7	positive	5.0	4.2	Tourlousse *et al.* (2020b)	modified GAM broth + 1% glucose, anaerobic (N_2 atmosphere), 37 °C, 24 h
Megamonas funiformis	JCM 14723[T]	CP048627<br>CP048628	GCA_010669225.1	2,568,766	31.5	-	5.0	0.0 [a]	Tourlousse *et al.* (2020c)	modified GAM broth, anaerobic (N_2 atmosphere), 37 °C, 24 h
Flavonifractor plautii	JCM 32125[T]	CP048436	GCA_010508875.1	3,985,392	60.9	positive	5.0	2.8	Tourlousse *et al.* (2020d)	modified GAM broth + 1% glucose, anaerobic (N_2 atmosphere), 37 °C, 24 h
Escherichia coli	NBRC 3301	CP048439<br>CP048440	GCA_010509415.1	4,755,096	50.8	negative	5.0	1.7	This study [b]	medium 702 [c], aerobic (180 rpm), 30 °C, 24 h
Akkermansia muciniphila	JCM 30893	CP048438	GCA_010509235.1	2,878,261	55.6	negative	5.0	2.7	This study [b]	modified GAM broth + 33 mM NaOAc, anaerobic (N_2 atmosphere), 37 °C, 24 h
Faecalibacterium prausnitzii	JCM 31915	CP048437	GCA_010509575.1	3,102,523	56.4	positive	5.0	6.2	This study [b]	modified GAM broth + 33 mM NaOAc, anaerobic (N_2 atmosphere), 37 °C, 24 h
Staphylococcus epidermidis	NBRC 100911[T]	AP019721<br>AP019722	GCA_006742205.1	2,427,041	32.3	positive	5.0	5.3	This study [b]	medium 702 [c], aerobic (180 rpm), 30 °C, 24 h
Cutibacterium acnes subsp. *acnes*	NBRC 107605[T]	AP019723	GCA_006739385.1	2,494,738	60.0	positive	5.0	7.9	This study [b]	modified GAM broth + 1% glucose, anaerobic (N_2 atmosphere), 37 °C, 48 h
Bacteroides uniformis	NBRC 113350	AP019724<br>AP019725<br>AP019726<br>AP019727<br>AP019728	GCA_006742345.1	4,989,532	46.2	negative	5.0	2.8	This study [b]	modified GAM broth + 1% glucose, anaerobic (N_2 atmosphere), 37 °C, 24 h
Enterocloster clostridioforme	NBRC 113352	BJLB01000001<br>BJLB01000002	GCA_006538465.1	5,687,315	48.9	positive	5.0	3.1	This study [b]	modified GAM broth + 1% glucose, anaerobic (N_2 atmosphere), 37 °C, 24 h
Bacillus subtilis subsp. *subtilis*	NBRC 13719[T]	AP019714<br>AP019715	GCA_006741845.1	4,295,305	43.3	positive	5.0	3.0	This study [b]	medium 702, aerobic (180 rpm), 30 °C, 24 h
Streptococcus mutans	NBRC 13955[T]	AP019720	GCA_006739205.1	2,018,796	36.9	positive	5.0	6.2	This study [b]	medium 310 [c], anaerobic (N_2 atmosphere), 37 °C, 24 h
Lactobacillus delbrueckii subsp. *delbrueckii*	NBRC 3202[T]	AP019750	GCA_006740305.1	1,910,306	50.1	positive	5.0	11.9	This study [b]	medium 804 [c], anaerobic (N_2 atmosphere), 30 °C, 24 h
Pseudomonas putida	NBRC 14164[T]	AP013070	GCA_000412675.1	6,156,701	62.3	negative	5.0	3.5		medium 702 [c], aerobic (180 rpm), 30 °C, 24 h
Bifidobacterium longum subsp. *longum*	JCM 1217[T]	AP010888	GCA_000196555.1	2,385,164	60.3	positive	5.0	19.1		modified GAM broth + 1% glucose, anaerobic (N_2 atmosphere), 37 °C, 24 h
Agathobacter rectalis	JCM 17463[T]	CP001107	GCA_000020605.1	3,449,685	41.5	positive	5.0	2.7		modified GAM broth + 33 mM NaOAc, anaerobic (N_2 atmosphere), 37 °C, 24 h
Megasphaera elsdenii	JCM 1772[T]	CP027570	GCA_003010495.1	2,478,942	52.8	-	5.0	0.0 [a]		modified GAM broth, anaerobic (N_2 atmosphere), 37 °C, 24 h
Parabacteroides distasonis	JCM 5825[T]	CP000140	GCA_000012845.1	4,811,379	45.1	negative	5.0	2.1		modified GAM broth + 1% glucose, anaerobic (N_2 atmosphere), 37 °C, 24 h
Bifidobacterium longum subsp. *infantis*	JCM 1222[T]	AP010889	GCA_000269965.1	2,828,958	59.9	positive	5.0	9.6		modified GAM broth + 1% glucose, anaerobic (N_2 atmosphere), 37 °C, 24 h

(a) Not included in the cell mock community.

(b) See Supplementary Methods for details.

(c) medium 310: https://www.nite.go.jp/nbrc/catalogue/NBRCMediumDetailServlet?NO=310
medium 702: https://www.nite.go.jp/nbrc/catalogue/NBRCMediumDetailServlet?NO=702
medium 804: https://www.nite.go.jp/nbrc/catalogue/NBRCMediumDetailServlet?NO=804

Supplementary Table S2. Description of kits / protocols for sequencing library construction.

kit name	company	method of DNA fragmentation	number of reaction steps / purifications	Required time (h)	500 ng input DNA (PCR-free)	50 ng input DNA (PCR cycles) (fragmentation time, enzymatic)	1 ng input DNA (PCR cycles) (fragmentation time, enzymatic)	one-letter abbreviation
Accel NGS 2S Plus DNA Library Kit	Swift Biosciences	physical	5 / 5 (PCR -)<br>6 / 6 (PCR +)	6 (PCR -)<br>7 (PCR)	•	•<br>(4 cycles)	•<br>(9 cycles)	A
QIAseq FX DNA Library Kit	Qiagen	enzymatic	2 / 3 (PCR -)<br>3 / 4 (PCR +)	4 (PCR -)<br>6 (PCR +)	•<br>(9 min)	•<br>(8 cycles)<br>(12 min)	•<br>(12)<br>(8 min)[a]	B
TruSeq (Nano) DNA (PCR-Free) Library Prep Kit	Illumina	physical	4 / 4 (PCR -)<br>5 /5 (PCR -)	7 (PCR -)<br>8 (PCR +)	•	•<br>(8 cycles)	•<br>(8 cycles)	C
KAPA HTP Library Preparation Kit	Roche	physical	4 / 5 (PCR -)<br>5 / 6 (PCR)	8 (PCR -)<br>9 (PCR)	•	•<br>(5 cycles)	•<br>(15 cycles)	D
KAPA HyperPrep (PCR-free) Kit	Roche	physical	3 / 2	4.5	•	•<br>(4 cycles)	•<br>(14 cycles)	E
KAPA HyperPrep + KAPA Frag	Roche	enzymatic	4 / 3	5.5	•<br>(10 min)	•<br>(4 cycles)<br>(10 min)	•<br>(14 cycles)<br>(10 min)	F
KAPA HTP + KAPA Frag	Roche	enzymatic	5 / 6 (PCR +)	9 (PCR +)		•<br>(5 cycles)<br>(10 min)	•<br>(15 cycles)<br>(10 min)	G
NEBNext Ultra II DNA Library Prep Kit	New England Biolabs	physical	5 /4	6		•<br>(4 cycles)	•<br>(9 cycles)	H
NEBNext Ultra II FS DNA Library Prep Kit	New England Biolabs	enzymatic	4 / 3	6		•<br>(4 cycles)<br>(15 min)	•<br>(9 cycles)<br>(15 min)	I
Nextera DNA Flex Library Prep Kit	Illumina	enzymatic	2 / 2	4.5		•<br>(5 cycles)<br>(15 min)	•<br>(12 cycles)<br>(15 min)	J
SMARTer ThruPLEX DNA-Seq Kit	Takara Bio	physical	4 / 3	5		•<br>(6 cycles)	•<br>(11 cycles)	K

[a] With FX Enhancer.

Supplementary Table S3. Target values for achievable performance of measurement results, in terms of acceptable level of errors (that is, differences between measured values and the "ground truth" for mock communities).

sample type	geometric mean strain-wise absolute fold differences	maximum strain-wise absolute fold differences
DNA mock community	1.15	1.5
Cell mock community	1.55	3.1

Supplementary Table S4. Description of kits / protocols for DNA extraction.

kit name	company	approximate time required (h)[a]	bead beating time	bead beating instrument	bead beating format	one-letter abbreviation
Extrap Soil DNA Kit Plus ver.2	NIPPON STEEL Eco-Tech Corporation	2	1 × 40 s<br>1 × 60 s<br>2 × 60 s<br>3 × 60 s<br>4 × 60 s	FastPrep-24	tube	L
FastDNA SPIN Kit for Feces	MP Biomedicals	3	1 × 40 s	FastPrep-24	tube	M
ISOSPIN Fecal DNA	Nippon Gene	2	1 × 40 s<br>1 × 60 s<br>2 × 60 s<br>3 × 60 s<br>4 × 60 s	FastPrep-24	tube	N
MagAttract PowerMicrobiome RNA/DNA EP Kit	Qiagen	4	2 × 600 s	TissueLyser II	plate	O
in-house phenol/chloroform-based protocol	-	3	1 × 40 s	FastPrep-24	tube	P
QIAamp DNA Stool Mini Kit based IHMS protocol Q	Qiagen	6	8 × 60 s	FastPrep-24	tube	Q
MORA-EXTRACT	Kyokuto Pharmaceutical	2	1 × 40 s	FastPrep-24	tube	R
QIAmp PowerFecal Pro DNA Kit	Qiagen	3	1 × 40 s<br>1 × 60 s<br>2 × 60 s<br>3 × 60 s<br>4 × 60 s	FastPrep-24	tube	S
Quick DNA Fecal/Soil microbe Miniprep Kit	Zymo Research	3	1 × 40 s	FastPrep-24	tube	T

[a] Estimated based on processing of 8 samples.

Supplementary Table S5. Overview of materials generated in the interlaboratory study for assessing transferability and reproducibility of our recommended protocols for DNA extraction and sequencing library construction.

	Laboratory [a]	A	B	C		D		E		F	G	H	I	J
	Protocol [b]	**SOP**	**SOP**	**SOP**	**custom**	**SOP**	**custom**	**SOP**	**custom**	**SOP**	**SOP**	**SOP**	**SOP**	**SOP**
DNA mock community	sequencing library [c]	●	●								●	●		
	sequencing data	●	●								●	●		
cell mock community	extracted DNA [d]	●	●	●	●	●	●	●	●	●	●	●	●	●
fecal sample S01	extracted DNA [d]	●	●	●	●	●	●	●	●	●	●	●	●	●
	sequencing data	●	●								●	●		
fecal samples S02,S03,S06,S13	sequencing data	●	●								●	●		

(a) All laboratories performed duplicate analysis for each of the samples.

(a) 'SOP' indicates standard operating procedures as developed in the first phase of this study, namely protocols N and BL for DNA extraction and library construction, respectively. 'Custom' indicates in-house protocols for DNA extraction used by three industry-based laboratories.

(c) Sequencing libraries from each participant were shipped to the central laboratory (denoted as laboratory A) for sequencing (NextSeq 500 instrument).

(d) Extracted DNAs from each participant were shipped to the central laboratory (denoted as laboratory A) for sequencing library construction (protocol BL) and sequencing (NextSeq 500 instrument).

Supplementary Table S6. Proposed target values for precision of measurement results and methods, as repeatability, intermediate precision and interlaboratory reproducibility.

sample type	repeatability	intermediate precision	interlaboratory reproducibility
DNA mock community	4%, for PCR-free protocols or protocols with low PCR cycles, *e.g.* starting from >50 ng of input DNA<br><br>6%, for protocols with high PCR cycles, *e.g.* starting from 1 ng of input DNA	6%	6%, with centralized sequencing or for PCR-free protocols or protocols with low PCR cycles, *e.g.* starting from >50 ng of input DNA<br><br>17%, with de-centralized sequencing and for PCR-free methods or low PCR cycles, e.g. starting from >50 ng of input DNA
Cell mock community	6%	6%	6%, with centralized sequencing<br>33%, with de-centralized sequencing
Feces	30%	30%	51%

Note: Values represent rounded one-sided 95% confidence intervals of precision estimates generated in this study.

Supplementary Table S7. List of external MOSAIC Standards Challenge datasets analyzed in this study.

fastq ID	read pairs	mean read length (R1|R2)	comment	fastq ID	read pairs	mean read length (R1|R2)	comment
sub-25BJ9kAL1XYjrjQtTcNcf4ks_1	18,647,995	149|149	-	sub-3Pt1n82h4ZmTgayHZCwyiSoJ_1	1,801	150|150	not analyzed
sub-25BJ9kAL1XYjrjQtTcNcf4ks_2	19,362,584	149|149	-	sub-3Pt1n82h4ZmTgayHZCwyiSoJ_2	17,845,710	150|150	-
sub-25BJ9kAL1XYjrjQtTcNcf4ks_3	20,790,237	149|149	-	sub-3Pt1n82h4ZmTgayHZCwyiSoJ_3	11,516,946	150|150	-
sub-25BJ9kAL1XYjrjQtTcNcf4ks_4	23,376,218	149|149	-	sub-3Pt1n82h4ZmTgayHZCwyiSoJ_4	16,641,498	150|150	-
sub-25BJ9kAL1XYjrjQtTcNcf4ks_5	19,044,655	149|149	-	sub-3Pt1n82h4ZmTgayHZCwyiSoJ_5	10,191,233	150|150	-
sub-25BJ9kAL1XYjrjQtTcNcf4ks_6	17,407,175	149|149	-	sub-3Pt1n82h4ZmTgayHZCwyiSoJ_6	17,533,221	150|150	-
sub-25BJ9kAL1XYjrjQtTcNcf4ks_7	27,197,690	149|149	-	sub-3Pt1n82h4ZmTgayHZCwyiSoJ_7	21,738,321	150|150	-
sub-53CE2XVESxyhcENCFpbetVLL_1	23,217,124	151|151	-	sub-8D3UBmhQRXdxY8biKkbb499G_1	16,509,360	75|75	fastp --length_required 50
sub-53CE2XVESxyhcENCFpbetVLL_2	22,289,812	151|151	-	sub-8D3UBmhQRXdxY8biKkbb499G_2	27,411,246	75|75	fastp --length_required 50
sub-53CE2XVESxyhcENCFpbetVLL_3	16,872,339	151|151	-	sub-8D3UBmhQRXdxY8biKkbb499G_3	32,377,707	75|75	fastp --length_required 50
sub-53CE2XVESxyhcENCFpbetVLL_4	16,486,851	151|151	-	sub-8D3UBmhQRXdxY8biKkbb499G_4	28,170,870	75|75	fastp --length_required 50
sub-53CE2XVESxyhcENCFpbetVLL_5	20,919,218	151|151	-	sub-8D3UBmhQRXdxY8biKkbb499G_5	22,322,868	75|75	fastp --length_required 50
sub-53CE2XVESxyhcENCFpbetVLL_6	14,035,308	151|151	-	sub-8D3UBmhQRXdxY8biKkbb499G_6	23,681,207	75|75	fastp --length_required 50
sub-53CE2XVESxyhcENCFpbetVLL_7	13,778,753	151|151	-	sub-8D3UBmhQRXdxY8biKkbb499G_7	25,922,046	75|75	fastp --length_required 50
sub-B9u5tS4okKnLtFqmESW1yvUe_1	14,649,192	151|151	-				_1 dataset not available
sub-B9u5tS4okKnLtFqmESW1yvUe_2	18,378,022	151|151	-	sub-Eaw1Z1Zhc2iq8p27c1iLyTwW_2	23,646,590	74|74	fastp --length_required 50
sub-B9u5tS4okKnLtFqmESW1yvUe_3	24,968,616	151|151	-	sub-Eaw1Z1Zhc2iq8p27c1iLyTwW_3	23,321,651	74|74	fastp --length_required 50
sub-B9u5tS4okKnLtFqmESW1yvUe_4	23,067,744	151|151	-	sub-Eaw1Z1Zhc2iq8p27c1iLyTwW_4	23,153,881	74|74	fastp --length_required 50
sub-B9u5tS4okKnLtFqmESW1yvUe_5	32,681,379	151|151	-	sub-Eaw1Z1Zhc2iq8p27c1iLyTwW_5	22,838,621	74|74	fastp --length_required 50
sub-B9u5tS4okKnLtFqmESW1yvUe_6	16,533,450	151|151	-	sub-Eaw1Z1Zhc2iq8p27c1iLyTwW_6	21,385,670	74|74	fastp --length_required 50
sub-B9u5tS4okKnLtFqmESW1yvUe_7	16,885,775	151|151	-	sub-Eaw1Z1Zhc2iq8p27c1iLyTwW_7	25,854,203	74|74	fastp --length_required 50
sub-cD69TaSgs3kiPLcjVTqzfkoQ_1	54,641,438	150|150	-	sub-Fb2pkVAPdAR2383mVGL7xCU9_1	46,576,557	150|150	-
sub-cD69TaSgs3kiPLcjVTqzfkoQ_2	45,221,672	150|150	-	sub-Fb2pkVAPdAR2383mVGL7xCU9_2	44,060,375	150|150	-
sub-cD69TaSgs3kiPLcjVTqzfkoQ_3	48,039,740	150|150	-	sub-Fb2pkVAPdAR2383mVGL7xCU9_3	50,071,554	150|150	-
sub-cD69TaSgs3kiPLcjVTqzfkoQ_4	49,274,107	150|150	-	sub-Fb2pkVAPdAR2383mVGL7xCU9_4	44,860,282	150|150	-
sub-cD69TaSgs3kiPLcjVTqzfkoQ_5	48,438,841	150|150	-	sub-Fb2pkVAPdAR2383mVGL7xCU9_5	47,455,206	150|150	-
sub-cD69TaSgs3kiPLcjVTqzfkoQ_6	43,247,105	150|150	-	sub-Fb2pkVAPdAR2383mVGL7xCU9_6	52,305,143	150|150	-
sub-cD69TaSgs3kiPLcjVTqzfkoQ_7	51,196,340	150|150	-	sub-Fb2pkVAPdAR2383mVGL7xCU9_7	46,602,971	150|150	-
sub-E8p1NnXxrsEyXsS4dUZaPwvk_1	47,331,443	150|150	-	sub-guUmhAUVNdW8cSXg25RfoMtq_1	15,202,770	151|151	-
sub-E8p1NnXxrsEyXsS4dUZaPwvk_2	49,304,567	150|150	-	sub-guUmhAUVNdW8cSXg25RfoMtq_2	21,043,846	151|151	-
sub-E8p1NnXxrsEyXsS4dUZaPwvk_3	46,829,561	150|150	-	sub-guUmhAUVNdW8cSXg25RfoMtq_3	21,543,820	151|151	-
sub-E8p1NnXxrsEyXsS4dUZaPwvk_4	53,691,345	150|150	-	sub-guUmhAUVNdW8cSXg25RfoMtq_4	16,737,118	151|151	-
sub-E8p1NnXxrsEyXsS4dUZaPwvk_5	49,012,854	150|150	-	sub-guUmhAUVNdW8cSXg25RfoMtq_5	31,292,056	151|151	-
sub-E8p1NnXxrsEyXsS4dUZaPwvk_6	52,451,118	150|150	-	sub-guUmhAUVNdW8cSXg25RfoMtq_6	25,454,069	151|151	-
sub-E8p1NnXxrsEyXsS4dUZaPwvk_7	55,085,550	150|150	-	sub-guUmhAUVNdW8cSXg25RfoMtq_7	13,366,665	151|151	-
sub-J3qqKYPx97PByMTVJeJKAUV9_1	20,126,154	149|149	-	sub-UinqmAp16Wo8TjCAMdAsK9WT_1	15,431,039	150|150	-
sub-J3qqKYPx97PByMTVJeJKAUV9_2	19,485,360	149|149	-	sub-UinqmAp16Wo8TjCAMdAsK9WT_2	16,820,032	150|150	-
sub-J3qqKYPx97PByMTVJeJKAUV9_3	24,173,799	149|149	-	sub-UinqmAp16Wo8TjCAMdAsK9WT_3	12,329,890	150|150	-
sub-J3qqKYPx97PByMTVJeJKAUV9_4	27,590,776	149|149	-	sub-UinqmAp16Wo8TjCAMdAsK9WT_4	12,444,202	150|150	-
sub-J3qqKYPx97PByMTVJeJKAUV9_5	22,162,572	149|149	-	sub-UinqmAp16Wo8TjCAMdAsK9WT_5	10,221,193	150|150	-
sub-J3qqKYPx97PByMTVJeJKAUV9_6	24,946,811	149|149	-	sub-UinqmAp16Wo8TjCAMdAsK9WT_6	16,323,600	150|150	-
sub-J3qqKYPx97PByMTVJeJKAUV9_7	26,514,143	149|149	-	sub-UinqmAp16Wo8TjCAMdAsK9WT_7	1,343,310	150|150	-
sub-KAWuEwoNYZL7voR7ZSkfYF34_1	18,576,854	149|149	-	sub-UKjbYYzAc76fiG6xGqNSfMRH_1	20,021,639	147|147	-
sub-KAWuEwoNYZL7voR7ZSkfYF34_2	23,877,292	149|149	-	sub-UKjbYYzAc76fiG6xGqNSfMRH_2	24,694,745	147|147	-
sub-KAWuEwoNYZL7voR7ZSkfYF34_3	29,902,282	149|149	-	sub-UKjbYYzAc76fiG6xGqNSfMRH_3	28,769,247	148|148	-
sub-KAWuEwoNYZL7voR7ZSkfYF34_4	24,908,413	149|149	-	sub-UKjbYYzAc76fiG6xGqNSfMRH_4	25,791,880	148|148	-
sub-KAWuEwoNYZL7voR7ZSkfYF34_5	32,379,583	149|149	-	sub-UKjbYYzAc76fiG6xGqNSfMRH_5	21,097,119	147|147	-
sub-KAWuEwoNYZL7voR7ZSkfYF34_6	17,649,937	149|149	-	sub-UKjbYYzAc76fiG6xGqNSfMRH_6	23,064,908	148|147	-
sub-KAWuEwoNYZL7voR7ZSkfYF34_7	28,576,466	149|149	-	sub-UKjbYYzAc76fiG6xGqNSfMRH_7	21,242,395	148|148	-

Supplementary Table S8. Overview of sequencing data sets generated in this study.

Experiment description: evaluation of the homogeneity of the DNA and cell mock community preparations and precision (between-run variability) of sequencing.

SRA accession	library ID	BioSample accession	metafield 1	metafield 2	metafield 3	metafield 4	read pairs
SRR12996255	PRSRX0934A	SAMN15699795	aliquot 1	replicate 1	protocol C0	N/A	22,535,131
SRR12996254	MTZKD2438Z	SAMN15699795	aliquot 1	replicate 2	protocol C0	N/A	20,481,396
SRR12996253	USSAV4874U	SAMN15699795	aliquot 2	replicate 1	protocol C0	N/A	23,178,960
SRR12996252	GLRVN7807X	SAMN15699795	aliquot 2	replicate 2	protocol C0	N/A	25,019,800
SRR12996250	LEYWQ8265B	SAMN15699795	aliquot 3	replicate 1	protocol C0	N/A	25,988,700
SRR12996249	NYXYT6131Q	SAMN15699795	aliquot 3	replicate 2	protocol C0	N/A	18,825,222
SRR12996222	XMAWC3253E	SAMN15699795	resequencing library PRSRX0934A	repeat 2	protocol C0	N/A	7,161,812
SRR12996221	VUOPT1962L	SAMN15699795	resequencing library PRSRX0934A	repeat 3	protocol C0	N/A	6,859,602
SRR12996210	VDZDY6239P	SAMN15699795	resequencing library PRSRX0934A	repeat 4	protocol C0	N/A	6,766,326
SRR12995891	MEFKO7750R	SAMN15699798	aliquot 1	replicate 1	protocol C	protocol BL	6,775,752
SRR12995890	JLGFD2112D	SAMN15699798	aliquot 1	replicate 2	protocol C	protocol BL	6,321,733
SRR12995889	VFULV9519Z	SAMN15699798	aliquot 2	replicate 1	protocol C	protocol BL	5,895,910
SRR12995888	ZXKQO1496G	SAMN15699798	aliquot 2	replicate 2	protocol C	protocol BL	6,792,133
SRR12995887	LBSQP1197V	SAMN15699798	aliquot 3	replicate 1	protocol C	protocol BL	6,805,020
SRR12995886	TPJUP1830J	SAMN15699798	aliquot 3	replicate 2	protocol C	protocol BL	6,158,654

Supplementary Table S8. Overview of sequencing data sets generated in this study, *continued*.

Experiment description: phase I, comparison of protocols for sequencing library construction.

SRA accession	library ID	BioSample accession	metafield 1	metafield 2	metafield 3	read pairs
SRR12996245	YPASQ0509K	SAMN15699795	protocol A0	Accel NGS 2S Plus DNA Library Kit	500 ng (X0)	9,290,896
SRR12996244	XGNLP7038C	SAMN15699795	protocol A0	Accel NGS 2S Plus DNA Library Kit	500 ng (X0)	7,924,370
SRR12996243	JBUIX5568V	SAMN15699795	protocol A0	Accel NGS 2S Plus DNA Library Kit	500 ng (X0)	7,534,289
SRR12996216	MIABL7113W	SAMN15699795	protocol AH	Accel NGS 2S Plus DNA Library Kit	1 ng (XH)	8,998,250
SRR12996215	KAZRM8350B	SAMN15699795	protocol AH	Accel NGS 2S Plus DNA Library Kit	1 ng (XH)	8,700,895
SRR12996214	NYXZY9866Z	SAMN15699795	protocol AH	Accel NGS 2S Plus DNA Library Kit	1 ng (XH)	9,132,783
SRR12996213	OOXIK5486P	SAMN15699795	protocol AL	Accel NGS 2S Plus DNA Library Kit	50 ng (XL)	8,272,566
SRR12996212	GMTGR1407W	SAMN15699795	protocol AL	Accel NGS 2S Plus DNA Library Kit	50 ng (XL)	8,291,342
SRR12996211	WDJOT3418B	SAMN15699795	protocol AL	Accel NGS 2S Plus DNA Library Kit	50 ng (XL)	7,700,685
SRR12996177	ONOLI8203G	SAMN15699795	protocol B0	QIAseq FX DNA Library Kit	500 ng (X0)	5,820,034
SRR12996176	SSUOU3710N	SAMN15699795	protocol B0	QIAseq FX DNA Library Kit	500 ng (X0)	7,785,985
SRR12996175	EWWUF5893S	SAMN15699795	protocol B0	QIAseq FX DNA Library Kit	500 ng (X0)	9,694,472
SRR12996184	EKMKV2003M	SAMN15699795	protocol BH	QIAseq FX DNA Library Kit	1 ng (XH)	3,666,747
SRR12996182	IPQGA1889J	SAMN15699795	protocol BH	QIAseq FX DNA Library Kit	1 ng (XH)	5,648,433
SRR12996181	WPACE6920A	SAMN15699795	protocol BH	QIAseq FX DNA Library Kit	1 ng (XH)	5,281,491
SRR12996180	VZRWH7215I	SAMN15699795	protocol BL	QIAseq FX DNA Library Kit	50 ng (XL)	3,066,761
SRR12996179	IEHKK7266N	SAMN15699795	protocol BL	QIAseq FX DNA Library Kit	50 ng (XL)	3,187,392
SRR12996178	RINGI6673U	SAMN15699795	protocol BL	QIAseq FX DNA Library Kit	50 ng (XL)	4,126,250
SRR12996248	QMBWW8147T	SAMN15699795	protocol C0	TruSeq (Nano) DNA (PCR-Free) Library Prep Kit	500 ng (X0)	13,770,875
SRR12996247	CAHHZ8466D	SAMN15699795	protocol C0	TruSeq (Nano) DNA (PCR-Free) Library Prep Kit	500 ng (X0)	13,842,322
SRR12996246	HREBM5403X	SAMN15699795	protocol C0	TruSeq (Nano) DNA (PCR-Free) Library Prep Kit	500 ng (X0)	11,489,517
SRR12996238	LUFSH1258S	SAMN15699795	protocol CH	TruSeq (Nano) DNA (PCR-Free) Library Prep Kit	1 ng (XH)	7,873,064
SRR12996237	DVJIC2603M	SAMN15699795	protocol CH	TruSeq (Nano) DNA (PCR-Free) Library Prep Kit	1 ng (XH)	7,967,612
SRR12996236	BRFMX1775K	SAMN15699795	protocol CH	TruSeq (Nano) DNA (PCR-Free) Library Prep Kit	1 ng (XH)	7,918,419
SRR12996235	IAGJN2467B	SAMN15699795	protocol CL	TruSeq (Nano) DNA (PCR-Free) Library Prep Kit	50 ng (XL)	7,206,326
SRR12996234	PHKHG5314R	SAMN15699795	C protocol L	TruSeq (Nano) DNA (PCR-Free) Library Prep Kit	50 ng (XL)	6,064,300
SRR12996233	ZZFIW8217R	SAMN15699795	protocol CL	TruSeq (Nano) DNA (PCR-Free) Library Prep Kit	50 ng (XL)	7,286,053
SRR12996242	SLVME9773T	SAMN15699795	protocol D0	KAPA HTP Library Preparation Kit	500 ng (X0)	16,802,831
SRR12996241	JVECG4894E	SAMN15699795	protocol D0	KAPA HTP Library Preparation Kit	500 ng (X0)	18,117,803
SRR12996239	PDZEP7730Z	SAMN15699795	protocol D0	KAPA HTP Library Preparation Kit	500 ng (X0)	17,702,854
SRR12996209	NFQSL1672A	SAMN15699795	protocol DH	KAPA HTP Library Preparation Kit	1 ng (XH)	10,142,444
SRR12996208	MDEZV6571Z	SAMN15699795	protocol DH	KAPA HTP Library Preparation Kit	1 ng (XH)	9,886,743
SRR12996204	FZWNX0246H	SAMN15699795	protocol DH	KAPA HTP Library Preparation Kit	1 ng (XH)	10,187,551
SRR12996203	KXSAM6874D	SAMN15699795	protocol DL	KAPA HTP Library Preparation Kit	50 ng (XL)	9,442,022
SRR12996202	QEZSB6901C	SAMN15699795	protocol DL	KAPA HTP Library Preparation Kit	50 ng (XL)	9,501,456
SRR12996201	VETOD3091P	SAMN15699795	protocol DL	KAPA HTP Library Preparation Kit	50 ng (XL)	9,534,327
SRR12996200	AQLAS7207P	SAMN15699795	protocol E0	KAPA HyperPrep (PCR-free) Kit	500 ng (X0)	11,525,380
SRR12996199	PXSXN2192Y	SAMN15699795	protocol E0	KAPA HyperPrep (PCR-free) Kit	500 ng (X0)	13,320,142
SRR12996198	XWMSK1794C	SAMN15699795	protocol E0	KAPA HyperPrep (PCR-free) Kit	500 ng (X0)	13,007,529
SRR12996225	QLMFJ6572R	SAMN15699795	protocol EH	KAPA HyperPrep (PCR-free) Kit	1 ng (XH)	9,231,695
SRR12996224	IJUTS6740L	SAMN15699795	protocol EH	KAPA HyperPrep (PCR-free) Kit	1 ng (XH)	9,089,610
SRR12996223	DPVUO1574H	SAMN15699795	protocol EH	KAPA HyperPrep (PCR-free) Kit	1 ng (XH)	10,171,471
SRR12996220	FQPVZ3967C	SAMN15699795	protocol EL	KAPA HyperPrep (PCR-free) Kit	50 ng (XL)	9,523,644
SRR12996219	WEZOA1238R	SAMN15699795	protocol EL	KAPA HyperPrep (PCR-free) Kit	50 ng (XL)	9,286,968
SRR12996217	QPQOV1893L	SAMN15699795	protocol EL	KAPA HyperPrep (PCR-free) Kit	50 ng (XL)	10,168,821
SRR12996154	WZSND9816K	SAMN15699795	protocol F0	KAPA HyperPrep + KAPA Frag	500 ng (X0)	10,113,254
SRR12996153	YMZAY7106F	SAMN15699795	protocol F0	KAPA HyperPrep + KAPA Frag	500 ng (X0)	11,125,433
SRR12996152	OKZVY1505D	SAMN15699795	protocol F0	KAPA HyperPrep + KAPA Frag	500 ng (X0)	11,081,338
SRR12996160	CVWLS4883I	SAMN15699795	protocol FH	KAPA HyperPrep + KAPA Frag	1 ng (XH)	7,825,147
SRR12996159	WYGHI2354Y	SAMN15699795	protocol FH	KAPA HyperPrep + KAPA Frag	1 ng (XH)	7,176,897
SRR12996158	UHLIK8571P	SAMN15699795	protocol FH	KAPA HyperPrep + KAPA Frag	1 ng (XH)	8,122,704
SRR12996157	TXQVM2545E	SAMN15699795	protocol FL	KAPA HyperPrep + KAPA Frag	50 ng (XL)	7,347,457
SRR12996156	VYEBW9961P	SAMN15699795	protocol FL	KAPA HyperPrep + KAPA Frag	50 ng (XL)	7,477,101
SRR12996155	EAWGW9546V	SAMN15699795	protocol FL	KAPA HyperPrep + KAPA Frag	50 ng (XL)	7,068,777
SRR12996167	MFMHR0336X	SAMN15699795	protocol GH	KAPA HTP + KAPA Frag	1 ng (XH)	7,933,522
SRR12996166	JEJBM2114H	SAMN15699795	protocol GH	KAPA HTP + KAPA Frag	1 ng (XH)	7,928,794
SRR12996165	VTDEF2260K	SAMN15699795	protocol GH	KAPA HTP + KAPA Frag	1 ng (XH)	7,265,340
SRR12996164	UIYDC6252L	SAMN15699795	protocol GL	KAPA HTP + KAPA Frag	50 ng (XL)	8,619,514
SRR12996163	NKTID8053X	SAMN15699795	protocol GL	KAPA HTP + KAPA Frag	50 ng (XL)	8,301,525
SRR12996162	PXKIM6024D	SAMN15699795	protocol GL	KAPA HTP + KAPA Frag	50 ng (XL)	7,707,914
SRR12996232	OYWKV3727D	SAMN15699795	protocol HH	NEBNext Ultra II DNA Library Prep Kit	1 ng (XH)	8,961,646
SRR12996231	AENUE8978L	SAMN15699795	protocol HH	NEBNext Ultra II DNA Library Prep Kit	1 ng (XH)	8,124,875
SRR12996230	ZHILX6222J	SAMN15699795	protocol HH	NEBNext Ultra II DNA Library Prep Kit	1 ng (XH)	9,993,106
SRR12996228	XYWYK0128G	SAMN15699795	protocol HL	NEBNext Ultra II DNA Library Prep Kit	50 ng (XL)	8,945,585
SRR12996227	NLYBD3171B	SAMN15699795	protocol HL	NEBNext Ultra II DNA Library Prep Kit	50 ng (XL)	9,146,250
SRR12996226	NTCPN6519G	SAMN15699795	protocol HL	NEBNext Ultra II DNA Library Prep Kit	50 ng (XL)	9,401,620
SRR12996197	PIMPV2601E	SAMN15699795	protocol IH	NEBNext Ultra II FS DNA Library Prep Kit	1 ng (XH)	10,617,823
SRR12996196	YGHKA9860M	SAMN15699795	protocol IH	NEBNext Ultra II FS DNA Library Prep Kit	1 ng (XH)	9,269,961
SRR12996195	ZNASJ4343U	SAMN15699795	protocol IH	NEBNext Ultra II FS DNA Library Prep Kit	1 ng (XH)	11,854,124
SRR12996193	ADDST7456U	SAMN15699795	protocol IL	NEBNext Ultra II FS DNA Library Prep Kit	50 ng (XL)	9,800,826
SRR12996192	LPFJI8941A	SAMN15699795	protocol IL	NEBNext Ultra II FS DNA Library Prep Kit	50 ng (XL)	10,162,727
SRR12996191	WPVKD1814M	SAMN15699795	protocol IL	NEBNext Ultra II FS DNA Library Prep Kit	50 ng (XL)	10,607,431
SRR12996190	YZFKH6948N	SAMN15699795	protocol JH	Nextera DNA Flex Library Prep Kit	1 ng (XH)	3,007,823
SRR12996189	TUPDT1718K	SAMN15699795	protocol JH	Nextera DNA Flex Library Prep Kit	1 ng (XH)	2,553,249
SRR12996188	YVZPY7425X	SAMN15699795	protocol JH	Nextera DNA Flex Library Prep Kit	1 ng (XH)	2,966,765
SRR12996187	VGSEN4896N	SAMN15699795	protocol JL	Nextera DNA Flex Library Prep Kit	50 ng (XL)	8,671,481
SRR12996186	ZDLTW7915V	SAMN15699795	protocol JL	Nextera DNA Flex Library Prep Kit	50 ng (XL)	2,929,741
SRR12996185	TNHUX0178E	SAMN15699795	protocol JL	Nextera DNA Flex Library Prep Kit	50 ng (XL)	1,807,446
SRR12996174	FAQBJ8354O	SAMN15699795	protocol KH	SMARTer ThruPLEX DNA-Seq Kit	1 ng (XH)	9,300,863
SRR12996173	RNVRF2397Q	SAMN15699795	protocol KH	SMARTer ThruPLEX DNA-Seq Kit	1 ng (XH)	7,812,631
SRR12996171	UDEUH2203G	SAMN15699795	protocol KH	SMARTer ThruPLEX DNA-Seq Kit	1 ng (XH)	6,161,994
SRR12996170	VPLWO9012T	SAMN15699795	protocol KL	SMARTer ThruPLEX DNA-Seq Kit	50 ng (XL)	9,342,866
SRR12996169	OBSBP7633H	SAMN15699795	protocol KL	SMARTer ThruPLEX DNA-Seq Kit	50 ng (XL)	8,206,503

SRR12996168	MRXSU2419K	SAMN15699795	protocol KL	SMARTer ThruPLEX DNA-Seq Kit	50 ng (XL)	9,362,506

Supplementary Table S8. Overview of sequencing data sets generated in this study, *continued*.

Experiment description: phase I, comparison of protocols for DNA extraction.

SRA accession	library ID	BioSample accession	metafield 1	metafield 2	metafield 3	read pairs
SRR12996151	UMSRP0980H	SAMN15699798	protocol Q	QIAmp DNA Stool Mini Kit based IHMS protocol Q	8x60s	7,108,991
SRR12996149	YMTQS4157I	SAMN15699798	protocol Q	QIAmp DNA Stool Mini Kit based IHMS protocol Q	8x60s	4,014,264
SRR12996148	GZAFM6535A	SAMN15699798	protocol Q	QIAmp DNA Stool Mini Kit based IHMS protocol Q	8x60s	4,689,076
SRR12996147	ASQJZ0615T	SAMN15699798	protocol N	ISOSPIN Fecal DNA	1x40s	4,657,404
SRR12996146	ZFOZD8577K	SAMN15699798	protocol N	ISOSPIN Fecal DNA	1x40s	5,088,964
SRR12996145	ZJXRR7209C	SAMN15699798	protocol N	ISOSPIN Fecal DNA	1x40s	4,836,397
SRR12996144	XBVGF0483S	SAMN15699798	protocol M	FastDNA SPIN Kit for Feces	1x40s	5,954,069
SRR12996143	CFYYO4072W	SAMN15699798	protocol M	FastDNA SPIN Kit for Feces	1x40s	3,718,780
SRR12996142	YNSEC6940T	SAMN15699798	protocol M	FastDNA SPIN Kit for Feces	1x40s	7,210,305
SRR12996141	OUFTB0627P	SAMN15699798	protocol M	FastDNA SPIN Kit for Feces	3x40s	4,973,562
SRR12996140	AOSHY0342I	SAMN15699798	protocol M	FastDNA SPIN Kit for Feces	3x40s	5,121,420
SRR12996138	CPMBK0854Y	SAMN15699798	protocol M	FastDNA SPIN Kit for Feces	3x40s	4,655,756
SRR12996137	MEWDB9259P	SAMN15699798	protocol R	MORA-EXTRACT	1x40s	4,363,712
SRR12996136	NMEVA8208M	SAMN15699798	protocol R	MORA-EXTRACT	1x40s	5,040,088
SRR12996135	GZVVR0204P	SAMN15699798	protocol R	MORA-EXTRACT	1x40s	4,247,550
SRR12996134	TQRYL0596A	SAMN15699798	protocol L	Extrap soil DNA kit plus ver.2	1x40s	7,467,228
SRR12996133	AOKIO8210V	SAMN15699798	protocol L	Extrap soil DNA kit plus ver.2	1x40s	6,305,885
SRR12996132	YIRIW9357L	SAMN15699798	protocol L	Extrap soil DNA kit plus ver.2	1x40s	4,500,411
SRR12996131	SIEGL6439A	SAMN15699798	protocol P	in-house phenol/chloroform-based protocol	1x40s	5,665,735
SRR12996130	IJBVM4020D	SAMN15699798	protocol P	in-house phenol/chloroform-based protocol	1x40s	5,663,143
SRR12996129	CKDMD2679G	SAMN15699798	protocol P	in-house phenol/chloroform-based protocol	1x40s	4,673,206
SRR12996127	GEVLN1207B	SAMN15699798	protocol T	Quick DNA Fecal/Soil microbe Miniprep Kit	1x40s	4,201,699
SRR12996126	OVEML0140P	SAMN15699798	protocol T	Quick DNA Fecal/Soil microbe Miniprep Kit	1x40s	5,588,747
SRR12996125	ZJHJD0408J	SAMN15699798	protocol T	Quick DNA Fecal/Soil microbe Miniprep Kit	1x40s	11,280,325
SRR12996124	MZYVP4154F	SAMN15699798	protocol S	QIAmp PowerFecal Pro DNA kit	1x40s	4,383,334
SRR12996123	AODHX9034L	SAMN15699798	protocol S	QIAmp PowerFecal Pro DNA kit	1x40s	4,149,477
SRR12996122	NOJBL2122Q	SAMN15699798	protocol S	QIAmp PowerFecal Pro DNA kit	1x40s	4,292,232
SRR12996121	WLDSC4184M	SAMN15699798	protocol O	MagAttract PowerMicrobiome RNA/DNA EP Kit	2x600s	5,866,353
SRR12996120	YNUKA4069O	SAMN15699798	protocol O	MagAttract PowerMicrobiome RNA/DNA EP Kit	2x600s	9,664,487
SRR12996119	GQHXN1601M	SAMN15699798	protocol O	MagAttract PowerMicrobiome RNA/DNA EP Kit	2x600s	10,329,932
SRR12996081	GOXWR5015Q	SAMN15699798	protocol N	ISOSPIN Fecal DNA	1x60s	7,410,632
SRR12996080	GQAOE1636R	SAMN15699798	protocol N	ISOSPIN Fecal DNA	1x60s	6,386,218
SRR12996079	PFMFG5612F	SAMN15699798	protocol N	ISOSPIN Fecal DNA	2x60s	5,933,342
SRR12996078	MBMMI0395X	SAMN15699798	protocol N	ISOSPIN Fecal DNA	2x60s	6,072,355
SRR12996077	KBKQW6012J	SAMN15699798	protocol N	ISOSPIN Fecal DNA	3x60s	6,483,516
SRR12996076	MOBQJ2004F	SAMN15699798	protocol N	ISOSPIN Fecal DNA	3x60s	7,074,006
SRR12996075	XPCAG2476N	SAMN15699798	protocol N	ISOSPIN Fecal DNA	4x60s	6,147,536
SRR12996074	ZVZBB6475A	SAMN15699798	protocol N	ISOSPIN Fecal DNA	4x60s	7,042,274
SRR12996073	MCTLD3828W	SAMN15699798	protocol S	QIAmp PowerFecal Pro DNA kit	1x60s	7,799,519
SRR12996071	FBBPG5503Y	SAMN15699798	protocol S	QIAmp PowerFecal Pro DNA kit	1x60s	5,531,082
SRR12996070	ZFFJN1274Y	SAMN15699798	protocol S	QIAmp PowerFecal Pro DNA kit	2x60s	6,447,932
SRR12996069	ROWML0997A	SAMN15699798	protocol S	QIAmp PowerFecal Pro DNA kit	2x60s	7,520,382
SRR12996068	TBJBQ6296R	SAMN15699798	protocol S	QIAmp PowerFecal Pro DNA kit	3x60s	7,515,070
SRR12996067	XPEWO8425V	SAMN15699798	protocol S	QIAmp PowerFecal Pro DNA kit	3x60s	7,327,786
SRR12996066	DGCIH3470D	SAMN15699798	protocol S	QIAmp PowerFecal Pro DNA kit	4x60s	6,632,018
SRR12996065	GRNLA6285L	SAMN15699798	protocol S	QIAmp PowerFecal Pro DNA kit	4x60s	5,335,760
SRR12996064	EDNVV5248B	SAMN15699798	protocol L	Extrap soil DNA kit plus ver.2	1x60s	5,278,333
SRR12996063	OZQHT2906R	SAMN15699798	protocol L	Extrap soil DNA kit plus ver.2	1x60s	6,102,972
SRR12996062	DMQCW2387A	SAMN15699798	protocol L	Extrap soil DNA kit plus ver.2	2x60s	5,181,573
SRR12996060	BHFPF3392H	SAMN15699798	protocol L	Extrap soil DNA kit plus ver.2	2x60s	4,997,703
SRR12996059	BOYIA0312C	SAMN15699798	protocol L	Extrap soil DNA kit plus ver.2	3x60s	4,537,694
SRR12996058	VJJAX0426M	SAMN15699798	protocol L	Extrap soil DNA kit plus ver.2	3x60s	4,943,826
SRR12996057	EGRIC4067N	SAMN15699798	protocol L	Extrap soil DNA kit plus ver.2	4x60s	5,448,457
SRR12996056	RLUBS0231Y	SAMN15699798	protocol L	Extrap soil DNA kit plus ver.2	4x60s	6,033,541

Supplementary Table S8. Overview of sequencing data sets generated in this study, *continued*.

Experiment description: phase I, comparison of protocols for DNA extraction.

SRA accession	library ID	BioSample accession	metafield 1	metafield 2	metafield 3	read pairs [a]
SRR12996055	YWUVP8943Y	SAMN15699786	protocol N	ISOSPIN Fecal DNA	1x60s	5,957,688
SRR12996054	KFVKY0109G	SAMN15699786	protocol N	ISOSPIN Fecal DNA	1x60s	5,815,146
SRR12996053	DTSLA7164L	SAMN15699786	protocol N	ISOSPIN Fecal DNA	2x60s	4,533,686
SRR12996052	GMIFJ4899N	SAMN15699786	protocol N	ISOSPIN Fecal DNA	2x60s	5,348,611
SRR12996051	OEPQJ0302L	SAMN15699786	protocol N	ISOSPIN Fecal DNA	3x60s	4,374,135
SRR12996049	HDZGC1853W	SAMN15699786	protocol N	ISOSPIN Fecal DNA	3x60s	4,699,862
SRR12996048	WRNCL4128T	SAMN15699786	protocol N	ISOSPIN Fecal DNA	4x60s	4,805,527
SRR12996047	PJHGN8994W	SAMN15699786	protocol N	ISOSPIN Fecal DNA	4x60s	9,964,360
SRR12996046	VLPBF9005Z	SAMN15699786	protocol S	QIAmp PowerFecal Pro DNA kit	1x60s	4,538,217
SRR12996045	IZDPO9942G	SAMN15699786	protocol S	QIAmp PowerFecal Pro DNA kit	1x60s	4,505,115
SRR12996044	BJAPB8482P	SAMN15699786	protocol S	QIAmp PowerFecal Pro DNA kit	2x60s	4,032,227
SRR12996043	XRDUO9845R	SAMN15699786	protocol S	QIAmp PowerFecal Pro DNA kit	2x60s	3,624,647
SRR12996042	KFAIW9232M	SAMN15699786	protocol S	QIAmp PowerFecal Pro DNA kit	3x60s	4,429,560
SRR12996041	FLFAY2943S	SAMN15699786	protocol S	QIAmp PowerFecal Pro DNA kit	3x60s	4,647,732
SRR12996040	MTDLX0365K	SAMN15699786	protocol S	QIAmp PowerFecal Pro DNA kit	4x60s	4,699,968
SRR12996038	ARCDE2885O	SAMN15699786	protocol S	QIAmp PowerFecal Pro DNA kit	4x60s	5,496,497
SRR12996037	SKGZX1334H	SAMN15699786	protocol L	Extrap soil DNA kit plus ver.2	1x60s	4,013,247
SRR12996036	WIYLN4124D	SAMN15699786	protocol L	Extrap soil DNA kit plus ver.2	1x60s	6,633,152
SRR12996035	LWIEJ9832I	SAMN15699786	protocol L	Extrap soil DNA kit plus ver.2	2x60s	5,778,107
SRR12996034	LEYTL5128O	SAMN15699786	protocol L	Extrap soil DNA kit plus ver.2	2x60s	4,240,473
SRR12996033	JJPVQ5578G	SAMN15699786	protocol L	Extrap soil DNA kit plus ver.2	3x60s	5,513,047
SRR12996032	RXNQP0432B	SAMN15699786	protocol L	Extrap soil DNA kit plus ver.2	3x60s	4,438,872
SRR12996031	JIQTH3662E	SAMN15699786	protocol L	Extrap soil DNA kit plus ver.2	4x60s	4,212,387
SRR12996030	YXVZN6388Z	SAMN15699786	protocol L	Extrap soil DNA kit plus ver.2	4x60s	6,410,810
SRR12995994	YXGJW9531V	SAMN15699786	protocol Q	QIAmp DNA Stool Mini Kit based IHMS protocol Q	8x60s	4,200,618
SRR12995993	VIOIA6564K	SAMN15699786	protocol Q	QIAmp DNA Stool Mini Kit based IHMS protocol Q	8x60s	4,136,112

(a) Represents the number of reads after quality filtering using fastp and removal of human genomic reads using BMTagger.

Supplementary Table S8. Overview of sequencing data sets generated in this study, *continued*.

Experiment description: phase II, intermediate precision of SOP for sequencing library construction.

SRA accession	library ID	BioSample accession	metafield 1	metafield 2	metafield 3	read pairs
SRR12996118	BSTQN9003B	SAMN15699795	protocol B0	operatorO3	lotLA	6,082,751
SRR12996116	HTGTC2841E	SAMN15699795	protocol B0	operatorO3	lotLA	5,256,743
SRR12996115	KUNME6128A	SAMN15699795	protocol B0	operatorO3	lotLB	5,247,177
SRR12996114	QHYWA2245M	SAMN15699795	protocol B0	operatorO3	lotLB	5,180,690
SRR12996113	TQLYA9800H	SAMN15699795	protocol B0	operatorO4	lotLA	5,703,169
SRR12996112	BCYMZ6298D	SAMN15699795	protocol B0	operatorO4	lotLA	6,366,432
SRR12996111	EEHNW3508Z	SAMN15699795	protocol B0	operatorO5	lotLB	6,196,599
SRR12996110	QVLFL7006F	SAMN15699795	protocol B0	operatorO5	lotLB	3,455,293
SRR12996109	DGHTT7151W	SAMN15699795	protocol BH	operatorO3	lotLA	2,946,804
SRR12996108	NRYAA2046L	SAMN15699795	protocol BH	operatorO3	lotLA	4,273,518
SRR12996107	TNVCT9086M	SAMN15699795	protocol BH	operatorO3	lotLB	4,022,006
SRR12996105	GAXHJ7700V	SAMN15699795	protocol BH	operatorO3	lotLB	4,169,714
SRR12996104	SHPTG6381O	SAMN15699795	protocol BH	operatorO4	lotLA	4,261,992
SRR12996103	ZEXNA3782R	SAMN15699795	protocol BH	operatorO4	lotLA	3,781,585
SRR12996102	QTGZX4886M	SAMN15699795	protocol BH	operatorO5	lotLB	4,422,846
SRR12996101	CYHWH0365H	SAMN15699795	protocol BH	operatorO5	lotLB	4,690,790
SRR12996100	WLRRN4683J	SAMN15699795	protocol KL	operatorO3	lotLA	4,861,637
SRR12996099	ZTGQV9613B	SAMN15699795	protocol KL	operatorO3	lotLA	4,725,636
SRR12996098	GGMFN2261P	SAMN15699795	protocol KL	operatorO3	lotLB	4,971,535
SRR12996097	MPFUY5926M	SAMN15699795	protocol KL	operatorO3	lotLB	4,825,505
SRR12996096	UGIGX6437I	SAMN15699795	protocol KL	operatorO4	lotLA	4,531,911
SRR12996093	GFCGT0046P	SAMN15699795	protocol KL	operatorO4	lotLA	4,774,741
SRR12996092	TATDW9143D	SAMN15699795	protocol KL	operatorO5	lotLB	10,438,487
SRR12996091	LLSSC6214G	SAMN15699795	protocol KL	operatorO5	lotLB	13,617,295
SRR12996090	LAUVU6210Y	SAMN15699795	protocol KH	operatorO3	lotLA	4,868,437
SRR12996089	FKEVP9367J	SAMN15699795	protocol KH	operatorO3	lotLA	3,532,679
SRR12996088	NTFBT1792N	SAMN15699795	protocol KH	operatorO3	lotLB	4,684,706
SRR12996087	TOSLN9315H	SAMN15699795	protocol KH	operatorO3	lotLB	4,376,207
SRR12996086	SWZVA0299T	SAMN15699795	protocol KH	operatorO4	lotLA	5,077,472
SRR12996085	ZYDUO5250N	SAMN15699795	protocol KH	operatorO4	lotLA	5,214,915
SRR12996084	VOTUQ3740M	SAMN15699795	protocol KH	operatorO5	lotLB	13,358,608
SRR12996082	LPFOU8828M	SAMN15699795	protocol KH	operatorO5	lotLB	4,938,281
SRR12995885	EPLGQ4076C	SAMN15699795	protocol BL	operatorO2	lotLA	6,138,184
SRR12995883	RHPHR9481N	SAMN15699795	protocol BL	operatorO2	lotLA	6,649,620
SRR12995882	RHDFZ6719S	SAMN15699795	protocol BL	operatorO2	lotLA	6,749,177
SRR12995881	KALQB4368Y	SAMN15699795	protocol BL	operatorO2	lotLB	5,999,022
SRR12995880	ZSWVQ6180M	SAMN15699795	protocol BL	operatorO2	lotLB	5,971,961
SRR12995879	WGZOZ6158E	SAMN15699795	protocol BL	operatorO2	lotLB	6,548,407
SRR12995878	NTXUW5961P	SAMN15699795	protocol BL	operatorO1	lotLA	6,822,646
SRR12995877	DTBXH2248I	SAMN15699795	protocol BL	operatorO1	lotLA	6,510,923
SRR12995876	BRIBF9522J	SAMN15699795	protocol BL	operatorO1	lotLA	6,030,151
SRR12995875	FQDQL2727I	SAMN15699795	protocol BL	operatorO3	lotLB	5,585,902
SRR12995874	FLYVT2013F	SAMN15699795	protocol BL	operatorO3	lotLB	6,817,860
SRR12996330	QUTAP9744N	SAMN15699795	protocol BL	operatorO3	lotLB	6,184,547

Supplementary Table S8. Overview of sequencing data sets generated in this study, *continued*.

Experiment description: phase II, intermediate precision of SOP for DNA extraction.

SRA accession	library ID	BioSample accession	metafield 1	metafield 2	metafield 3	read pairs [a]
SRR12996029	AIGKO0991S	SAMN15699798	protocol N	operatorO2	lotLA	4,810,741
SRR12996027	BREPU1754Z	SAMN15699798	protocol N	operatorO2	lotLA	4,656,599
SRR12996026	MQRDV1049C	SAMN15699798	protocol N	operatorO2	lotLB	4,796,068
SRR12996025	DNCHK1414K	SAMN15699798	protocol N	operatorO2	lotLB	4,296,323
SRR12996024	JARDL6168V	SAMN15699798	protocol N	operatorO1	lotLA	5,336,502
SRR12996023	VSUKT5351G	SAMN15699798	protocol N	operatorO1	lotLA	4,611,111
SRR12996022	RCLHA3850I	SAMN15699798	protocol N	operatorO3	lotLB	4,218,789
SRR12996021	BJSOI3318J	SAMN15699798	protocol N	operatorO3	lotLB	4,684,218
SRR12996020	UJRRG9052S	SAMN15699786	protocol N	operatorO2	lotLA	4,545,345
SRR12996019	FHPLO7597Z	SAMN15699786	protocol N	operatorO2	lotLA	5,277,012
SRR12996018	LSXQY2856U	SAMN15699786	protocol N	operatorO2	lotLB	4,230,081
SRR12996016	DOTWR8396Z	SAMN15699786	protocol N	operatorO2	lotLB	4,120,061
SRR12996015	HCAXY4772M	SAMN15699786	protocol N	operatorO1	lotLA	4,730,268
SRR12996014	RLHXK7377U	SAMN15699786	protocol N	operatorO1	lotLA	4,273,669
SRR12996013	UNGHN7449K	SAMN15699786	protocol N	operatorO3	lotLB	4,151,683
SRR12996012	OAIFZ5345N	SAMN15699786	protocol N	operatorO3	lotLB	4,283,747

[a] Represents the number of reads after quality filtering using fastp and removal of human genomic reads using BMTagger.

Supplementary Table S8. Overview of sequencing data sets generated in this study, *continued*.

Experiment description: phase II, evaluation of interlaboratory reproducibility of DNA extraction, sequencing at central laboratory.

SRA accession	library ID	BioSample accession	metafield 1	metafield 2	read pairs [a]
SRR12995948	WABDO0924F	SAMN15699798	SOP, protocol N	participant B	10,311,559
SRR12995947	RWVZL0907I	SAMN15699798	SOP, protocol N	participant B	8,966,932
SRR12995944	TMJYK2442Z	SAMN15699798	SOP, protocol N	participant A	7,588,330
SRR12995943	VQBKV1974H	SAMN15699798	SOP, protocol N	participant A	7,129,379
SRR12995940	RQXEC8570G	SAMN15699798	SOP, protocol N	participant C	7,355,961
SRR12995938	DTJPB2970M	SAMN15699798	SOP, protocol N	participant C	8,644,593
SRR12995935	KEHKC1228G	SAMN15699798	SOP, protocol N	participant D	8,536,691
SRR12995934	ATAVE8334Z	SAMN15699798	SOP, protocol N	participant D	9,543,363
SRR12995931	UJVXV1823D	SAMN15699798	SOP, protocol N	participant E	10,270,585
SRR12995930	KNLLC2446A	SAMN15699798	SOP, protocol N	participant E	8,323,900
SRR12995926	ATYMI5294H	SAMN15699798	SOP, protocol N	participant F	6,465,232
SRR12995925	IHNRY1739N	SAMN15699798	SOP, protocol N	participant F	7,162,489
SRR12995922	EJBMI9924U	SAMN15699798	SOP, protocol N	participant G	7,408,969
SRR12995921	HIEJD9440W	SAMN15699798	SOP, protocol N	participant G	7,295,171
SRR12995909	VGSFV5166T	SAMN15699798	SOP, protocol N	participant H	7,991,366
SRR12995908	EWHJE9040S	SAMN15699798	SOP, protocol N	participant H	7,828,851
SRR12995904	HALOF4969M	SAMN15699798	SOP, protocol N	participant I	7,999,324
SRR12995903	YEDUY1294I	SAMN15699798	SOP, protocol N	participant I	7,897,182
SRR12995900	ZRETT9751I	SAMN15699798	SOP, protocol N	participant J	6,659,704
SRR12995899	FZQMY3335Z	SAMN15699798	SOP, protocol N	participant J	7,798,145
SRR12996329	ZYNNG0310D	SAMN15699798	custom protocol	participant X	8,683,955
SRR12996323	AKAVR7329K	SAMN15699798	custom protocol	participant Y	7,390,323
SRR12996316	JCMSQ7032W	SAMN15699798	custom protocol	participant Z	7,117,949
SRR12995946	ZKVGB2111H	SAMN15699786	SOP, protocol N	participant B	6,342,825
SRR12995945	OIWFA5304H	SAMN15699786	SOP, protocol N	participant B	4,861,231
SRR12995942	RGHFL7941S	SAMN15699786	SOP, protocol N	participant A	12,773,847
SRR12995941	WRCST3409W	SAMN15699786	SOP, protocol N	participant A	6,267,641
SRR12995937	TTXRG9536D	SAMN15699786	SOP, protocol N	participant C	6,130,330
SRR12995936	FYLUD8222Z	SAMN15699786	SOP, protocol N	participant C	7,866,506
SRR12995933	FZJFT5976J	SAMN15699786	SOP, protocol N	participant D	6,437,984
SRR12995932	WAWCF1743N	SAMN15699786	SOP, protocol N	participant D	5,569,805
SRR12995929	CYWOJ1520P	SAMN15699786	SOP, protocol N	participant E	8,260,909
SRR12995927	NVAFE4837E	SAMN15699786	SOP, protocol N	participant E	5,593,399
SRR12995924	MZLXM1673O	SAMN15699786	SOP, protocol N	participant F	6,484,020
SRR12995923	WVNNO5035A	SAMN15699786	SOP, protocol N	participant F	9,002,565
SRR12995920	UYQDB7695P	SAMN15699786	SOP, protocol N	participant G	5,413,289
SRR12995919	VLMUF8356J	SAMN15699786	SOP, protocol N	participant G	5,429,593
SRR12995907	RCFSM7922K	SAMN15699786	SOP, protocol N	participant H	7,885,270
SRR12995905	HLRDB2907E	SAMN15699786	SOP, protocol N	participant H	6,640,917
SRR12995902	GXDNI6943S	SAMN15699786	SOP, protocol N	participant I	7,066,269
SRR12995901	RYGZC0889D	SAMN15699786	SOP, protocol N	participant I	7,572,322
SRR12995898	MEHCW7085V	SAMN15699786	SOP, protocol N	participant J	6,200,558
SRR12995897	XDIJF7756P	SAMN15699786	SOP, protocol N	participant J	5,858,436
SRR12996328	ESQMQ1093L	SAMN15699786	custom protocol	participant X	6,196,835
SRR12996322	KVUCR1822O	SAMN15699786	custom protocol	participant Y	7,787,135
SRR12996315	VKKOS6176H	SAMN15699786	custom protocol	participant Z	6,124,431

(a) Represents the number of reads after quality filtering using fastp and removal of human genomic reads using BMTagger for human fecal samples.

Supplementary Table S8. Overview of sequencing data sets generated in this study, *continued*.

Experiment description: phase II, evaluation of interlaboratory reproducibility of library construction, centralized and de-centralized sequencing.

SRA accession	library ID	BioSample accession	comment	metafield 1	metafield 2	metafield 3	read pairs
SRR12995918	PMWWX7931G	SAMN15699795	sequencing at central laboratory	protocol BL	NextSeq	participant A	8,976,686
SRR12995916	XGFOA3403V	SAMN15699795	sequencing at central laboratory	protocol BL	NextSeq	participant A	9,141,367
SRR12995913	RXRZN2443W	SAMN15699795	sequencing at central laboratory	protocol BL	NextSeq	participant G	8,317,429
SRR12995912	JHAOB7730R	SAMN15699795	sequencing at central laboratory	protocol BL	NextSeq	participant G	7,600,333
SRR12995896	QJPSA6625N	SAMN15699795	sequencing at central laboratory	protocol BL	NextSeq	participant B	7,954,891
SRR12995894	EOGNZ8674Y	SAMN15699795	sequencing at central laboratory	protocol BL	NextSeq	participant B	8,563,310
SRR12995893	WZGYR2679O	SAMN15699795	sequencing at central laboratory	protocol BL	NextSeq	participant H	7,515,370
SRR12995892	RYGLM0197P	SAMN15699795	sequencing at central laboratory	protocol BL	NextSeq	participant H	8,362,936
SRR12996207	MXIMY6427O	SAMN15699795	sequencing at participant's laboratory	protocol BL	NextSeq	participant A	12,922,997
SRR12996206	YHTLQ6589U	SAMN15699795	sequencing at participant's laboratory	protocol BL	NextSeq	participant A	11,055,206
SRR12996295	WQBQG7285P	SAMN15699795	sequencing at participant's laboratory	protocol BL	NovaSeq	participant G	43,801,210
SRR12996293	WPRTX2513P	SAMN15699795	sequencing at participant's laboratory	protocol BL	NovaSeq	participant G	36,544,086
SRR12996287	HSKPA1111Y	SAMN15699795	sequencing at participant's laboratory	protocol BL	NextSeq	participant B	12,260,151
SRR12996276	KWZGX5541H	SAMN15699795	sequencing at participant's laboratory	protocol BL	NextSeq	participant B	11,511,547
SRR12995983	GWFSG5897X	SAMN15699795	sequencing at participant's laboratory	protocol BL	NextSeq	participant H	12,599,277
SRR12995972	QMIVB9612B	SAMN15699795	sequencing at participant's laboratory	protocol BL	NextSeq	participant H	11,320,943

Supplementary Table S8. Overview of sequencing data sets generated in this study, *continued*.

Experiment description: phase II, evaluation of interlaboratory reproducibility, externally generated sequencing data.

SRA accession	library ID	BioSample accession	metafield 1	metafield 2	metafield 3	read pairs [a]
SRR12996229	ANGBX9820C	SAMN15699786	SOP, protocol N + protocol BL	NextSeq	participant A	11,301,377
SRR12996218	ZRKZL8955B	SAMN15699786	SOP, protocol N + protocol BL	NextSeq	participant A	10,868,076
SRR12996205	JOSEO9921H	SAMN15699787	SOP, protocol N + protocol BL	NextSeq	participant A	10,110,273
SRR12996194	KFKDT9009E	SAMN15699787	SOP, protocol N + protocol BL	NextSeq	participant A	11,777,944
SRR12996183	ZXHWM1122G	SAMN15699788	SOP, protocol N + protocol BL	NextSeq	participant A	10,492,271
SRR12996172	CEEUT7387Z	SAMN15699788	SOP, protocol N + protocol BL	NextSeq	participant A	9,914,289
SRR12996161	UADNC2804K	SAMN15699790	SOP, protocol N + protocol BL	NextSeq	participant A	10,850,366
SRR12996150	KYQDO6191J	SAMN15699790	SOP, protocol N + protocol BL	NextSeq	participant A	12,555,300
SRR12996139	YYUDB1506I	SAMN15699792	SOP, protocol N + protocol BL	NextSeq	participant A	10,215,600
SRR12996128	AWJCU2905O	SAMN15699792	SOP, protocol N + protocol BL	NextSeq	participant A	9,811,892
SRR12995961	IREFK2660B	SAMN15699786	SOP, protocol N + protocol BL	NextSeq	participant H	10,858,218
SRR12995950	BJHNY0431U	SAMN15699786	SOP, protocol N + protocol BL	NextSeq	participant H	10,281,811
SRR12995939	IKEAP7919B	SAMN15699787	SOP, protocol N + protocol BL	NextSeq	participant H	10,444,678
SRR12995928	BDBNB3719L	SAMN15699787	SOP, protocol N + protocol BL	NextSeq	participant H	10,465,135
SRR12995917	XNTWA2725M	SAMN15699788	SOP, protocol N + protocol BL	NextSeq	participant H	11,578,595
SRR12995906	MFCBT5445R	SAMN15699788	SOP, protocol N + protocol BL	NextSeq	participant H	10,933,311
SRR12995895	QPFGR0377T	SAMN15699790	SOP, protocol N + protocol B0	NextSeq	participant H	10,704,919
SRR12995884	VTWUF8642A	SAMN15699790	SOP, protocol N + protocol BL	NextSeq	participant H	11,034,716
SRR12995872	BNGFT5521W	SAMN15699792	SOP, protocol N + protocol BL	NextSeq	participant H	10,545,001
SRR12996320	ZZDCI3118C	SAMN15699792	SOP, protocol N + protocol BL	NextSeq	participant H	10,761,253
SRR12996266	BIQAZ9448F	SAMN15699786	SOP, protocol N + protocol BL	NextSeq	participant B	9,113,166
SRR12996265	DSZQE7617U	SAMN15699786	SOP, protocol N + protocol BL	NextSeq	participant B	11,568,775
SRR12996264	QKGYU2770E	SAMN15699787	SOP, protocol N + protocol BL	NextSeq	participant B	10,619,824
SRR12996263	UZJDU9681M	SAMN15699787	SOP, protocol N + protocol BL	NextSeq	participant B	10,658,483
SRR12996261	EPPAU1110G	SAMN15699788	SOP, protocol N + protocol BL	NextSeq	participant B	5,743,969
SRR12996260	VEXTL8309O	SAMN15699788	SOP, protocol N + protocol BL	NextSeq	participant B	8,651,921
SRR12996259	PKWYB6370Y	SAMN15699790	SOP, protocol N + protocol BL	NextSeq	participant B	11,605,019
SRR12996258	YZZVL8708E	SAMN15699790	SOP, protocol N + protocol BL	NextSeq	participant B	10,565,429
SRR12996257	FECWJ6257P	SAMN15699792	SOP, protocol N + protocol BL	NextSeq	participant B	10,741,726
SRR12996256	BPELE2373Q	SAMN15699792	SOP, protocol N + protocol BL	NextSeq	participant B	9,812,153
SRR12996286	BOCVG1170S	SAMN15699786	SOP, protocol N + protocol BL	NovaSeq	participant G	35,066,238
SRR12996284	YCTZX5304X	SAMN15699786	SOP, protocol N + protocol BL	NovaSeq	participant G	38,715,143
SRR12996282	XSCNG2308K	SAMN15699787	SOP, protocol N + protocol BL	NovaSeq	participant G	32,246,802
SRR12996280	SFALK7272X	SAMN15699787	SOP, protocol N + protocol BL	NovaSeq	participant G	40,156,839
SRR12996278	AHQJU9427M	SAMN15699788	SOP, protocol N + protocol BL	NovaSeq	participant G	36,059,125
SRR12996275	PRNIF2521D	SAMN15699788	SOP, protocol N + protocol BL	NovaSeq	participant G	39,312,556
SRR12996273	QSQWY8216Z	SAMN15699790	SOP, protocol N + protocol BL	NovaSeq	participant G	36,084,757
SRR12996271	GFIPN6743M	SAMN15699790	SOP, protocol N + protocol BL	NovaSeq	participant G	39,992,317
SRR12996269	INBPJ2435S	SAMN15699792	SOP, protocol N + protocol BL	NovaSeq	participant G	39,243,168
SRR12996267	CUCBE9450U	SAMN15699792	SOP, protocol N + protocol BL	NovaSeq	participant G	40,892,764

(a) Represents the number of reads after quality filtering using fastp and removal of human genomic reads using BMTagger for human fecal samples.

Supplementary references

Satterthwaite FE. 1946. An approximate distribution of estimates of variance components. Biometrics Bulletin. 2(6):110–114.

Pawlowsky-Glahn V, Egozcue JJ, Tolosano-Delgado R. 2007. Lecture notes on compositional data analysis, http://hdl.handle.net/10256/297.

Aitchison J. The statistical analysis of compositional data. Chapman and Hall, London, UK, 1986.

van den Boogaart KG, Tolosana-Delgado R, Bren M. 2020. compositions: Compositional Data Analysis. R package version 2.0-0. https://CRAN.R-project.org/package=compositions

Welch BL. 1947. The generalisation of student's problems when several different population variances are involved. Biometrika. 34(1-2):28-35.

Wick RR, Judd LM, Gorrie CL, Holt, KE. 2017. Unicycler: Resolving bacterial genome assemblies from short and long sequencing reads. PLoS Comput Biol 13(6):e1005595.

Koren S, Walenz BP, Berlin K, Miller JR, Bergman NH, Phillippy AM. 2017. Canu: scalable and accurate long-read assembly via adaptive k-mer weighting and repeat separation. Genome Res 27(5):722-736.

Parks DH, Imelfort M, Skennerton CT, Hugenholtz P, Tyson GW. 2015. CheckM: assessing the quality of microbial genomes recovered from isolates, single cells, and metagenomes. Genome Res 25(7):1043-1055.

Tourlousse DM, Sakamoto M, Miura T, Narita K, Ohashi A, Uchino Y, Yamazoe A, Kameyama K, Terauchi J, Ohkuma M, Kawasaki H, Sekiguchi Y. 2020a. Complete Genome Sequence of Collinsella aerofaciens JCM 10188T. Microbiol Resour Announc 9(16):e00134-20.

Tourlousse DM, Sakamoto M, Miura T, Narita K, Ohashi A, Uchino Y, Yamazoe A, Kameyama K, Terauchi J, Ohkuma M, Kawasaki H, Sekiguchi Y. 2020b. Complete Genome Sequence of Blautia producta JCM 1471T. Microbiol Resour Announc. 9(17):e00141-20.

Tourlousse DM, Sakamoto M, Miura T, Narita K, Ohashi A, Uchino Y, Yamazoe A, Kameyama K, Terauchi J, Ohkuma M, Kawasaki H, Sekiguchi Y. 2020c. Complete Genome Sequence of Megamonas funiformis JCM 14723T. Microbiol Resour Announc 9(16):e00142-20.

Tourlousse DM, Sakamoto M, Miura T, Narita K, Ohashi A, Uchino Y, Yamazoe A, Kameyama K, Terauchi J, Ohkuma M, Kawasaki H, Sekiguchi Y. 2020d. Complete Genome Sequence of Flavonifractor plautii JCM 32125T. Microbiol Resour Announc. 9(17):e00135-20.
